# Evolution in arid environments: the role of betalains, succulence, and medullary bundles

**DOI:** 10.1111/nph.71521

**Published:** 2026-09-03

**Authors:** Tom Carruthers, Samuel F. Brockington, Hiroshi A. Maeda, Gregory W. Stull, Nathanael Walker‐Hale, Ya Yang, Stephen A. Smith

**Affiliations:** ^1^ Department of Ecology and Evolutionary Biology University of Michigan Ann Arbor MI 48109 USA; ^2^ Department of Plant Sciences University of Cambridge Downing Street Cambridge CB2 3EA UK; ^3^ Department of Botany University of Wisconsin‐Madison 132 Birge Hall, 430 Lincoln Drive Madison WI 53706 USA; ^4^ Department of Botany, National Museum of Natural History Smithsonian Institution Washington DC 20560 USA; ^5^ Department of Biosciences Durham University Stockton Road Durham DH1 3LE UK; ^6^ Department of Plant and Microbial Biology University of Minnesota‐Twin Cities 714 Biological, Sciences Center, 1445 Gortner Avenue St. Paul MN 55108‐1095 USA

**Keywords:** betalains, Caryophyllales, drought, fossils, phylogenetics, succulence

## Abstract

- The flowering plant clade Caryophyllales includes many succulent species that grow in drought conditions. Many Caryophyllales species also have betalain pigmentation and medullary bundles, both of which have been linked to stress tolerance, albeit with less precise functional characterisation. We investigated the relationship between the evolution of these traits and drought tolerance in Caryophyllales.
- We estimated divergence times for Caryophyllales using transcriptomic data; a 50‐character morphological matrix based on a comprehensive review of Caryophyllales fossils; a species‐level molecular dataset incorporating > 4400 species; and a tip‐dating approach. This enabled us to analyse the evolution of drought tolerance, succulence, betalain pigmentation, and medullary bundles.
- We demonstrate that betalain‐pigmented lineages primarily experience drought conditions, with betalains appearing to evolve repeatedly in response to drought during the early evolution of Caryophyllales. We also show that the evolution of succulence and medullary bundles is favoured in betalain‐pigmented lineages. However, medullary bundles, unlike succulence, do not show a clear relationship with drought conditions.
- The evolution of betalains is likely to have enabled Caryophyllales to adapt to and survive stressful conditions, including drought. This may have increased the adaptive advantages of succulence, as its extensive costs are likely offset by its benefits in drought conditions.

## Graphical abstract

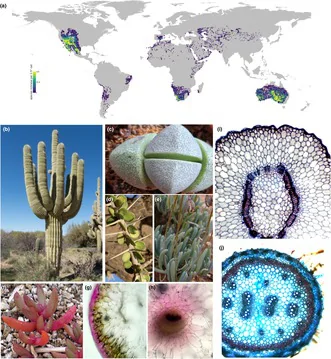

## Introduction

Since colonising land, plants have contended with water limitation. The degree of water limitation varies widely among flowering plants because they inhabit nearly all terrestrial ecosystems. Differences in water availability shape trait evolution, species distributions, and the timing and tempo of diversification (Ogburn & Edwards, 2013; Folk *et al*., 2019).

Extreme water shortage can have several interacting causes. The most obvious is very low or highly seasonal rainfall. For example, arid climates, which extend north and south from *c*. 10° north and south of the equator, receive < 20% of the annual rainfall of regions nearer the equator, and this rainfall is often highly seasonal (Houze *et al*., 2015). Rainfall is not the only cause of a water shortage, however. High temperatures, often overlapping with low rainfall (Cook *et al*., 2014), and soil composition, also dramatically influence water availability (e.g. Lichter‐Marck & Baldwin, 2023). Soil composition can underpin highly localised areas with extreme water limitation for plants, such as sandy coastal areas or rocky outcrops.

Very limited water availability poses challenges because plants require water to maintain turgor pressure for growth and structural integrity, and to achieve appropriate solute concentrations for key metabolic processes (Hsiao, 1973; Nogués & Baker, 2000; Ali *et al*., 2022). Insufficient water can therefore cause reduced growth, structural damage, accumulation of harmful metabolic byproducts (e.g. H_2_O_2_ and associated oxidative stress), and reduced metabolic efficiency (Marchesini *et al*., 2014; Jain & Gould, 2015; Wang *et al*., 2018). Several traits have evolved that enable flowering plants to overcome these challenges. These include: succulent water‐storing stems or leaves (Arakaki *et al*., 2011); enzymes or metabolites, including pigments and antioxidants, that prevent accumulation of harmful metabolic byproducts (Demidchik, 2015; Jain & Gould, 2015); the ability to accumulate solutes that lower cellular water potential and enable water uptake from dry environments (Hsiao *et al*., 1976); photosynthetic adaptations that improve water use efficiency (Sage, 2001); or vascular modifications enabling greater control of water and nutrient transport (Mauseth, 1993).

Caryophyllales is a major clade of flowering plants comprising *c*. 12 000 species (APG IV, 2016). They are distributed throughout the southern and northern hemispheres in habitats ranging from tropical rainforests to tundra and deserts (Yang *et al*., 2017). Importantly, Caryophyllales are speciose in arid climates across Africa, the Americas, Australia, and Asia (Fig. 1, Supporting Information Fig. S1; Table S1).

**Fig. 1:**
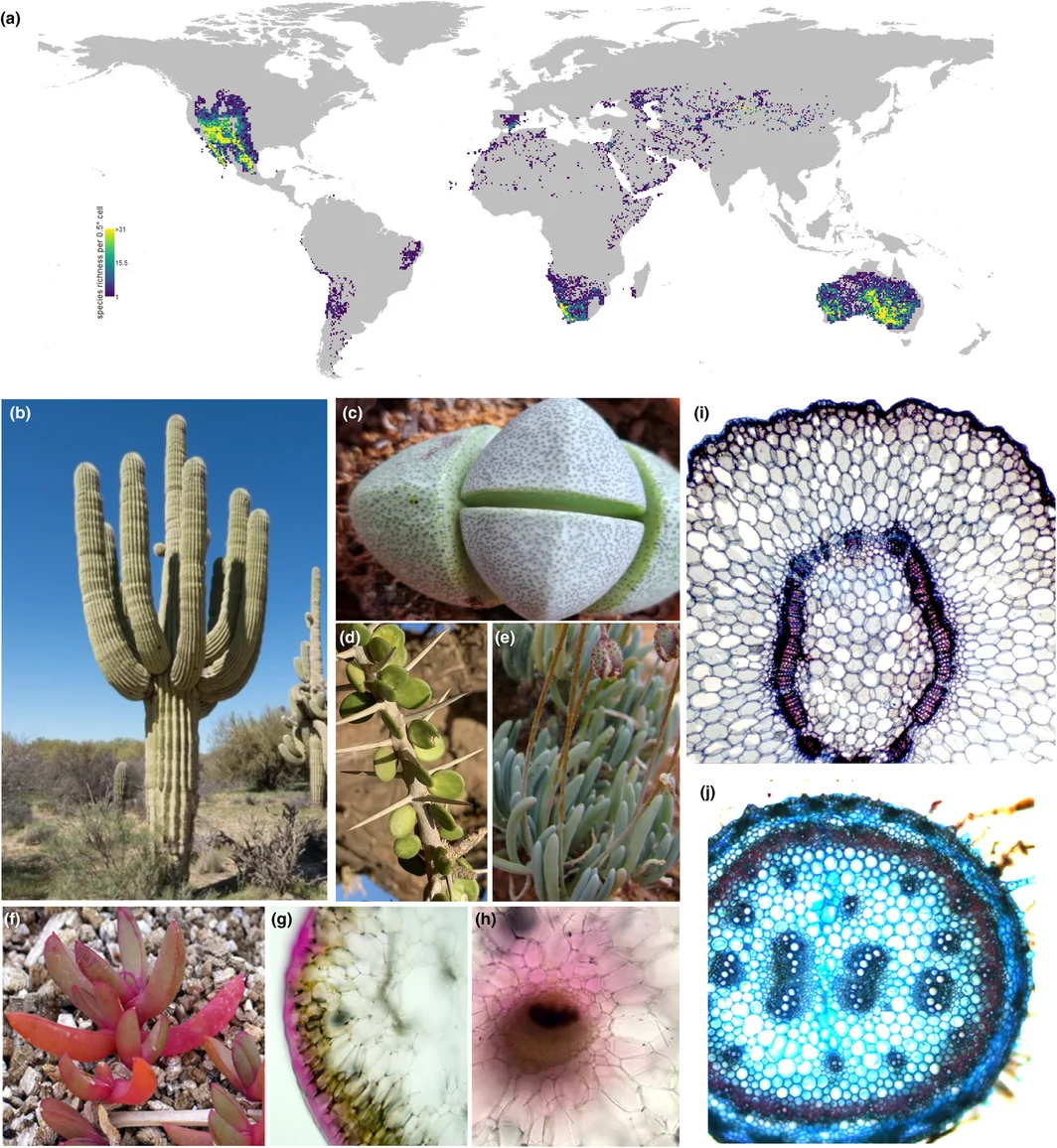
Overview of the study system. (a) Distribution of Caryophyllales species diversity in arid environments (i.e. areas defined as arid in the Köppen climate classification). (b–h) Examples of succulence in families throughout Caryophyllales. (b) *Carnegiea gigantea* (Cactaceae, photograph: Steve Jones), (c) *Lithops naureeniae* (Aizoaceae, photograph: Tom Carruthers), (d) *Alluaudia comosa* (Didiereaceae, photograph: Bert Harris), (e) *Kewa salsoloides* (Kewaceae, photograph: Lizelle Erasmus). (f–h) Betalain pigmentation in *Disphyma australe* (Aizoaceae). (f) Visibly affecting the external appearance of the plant, (g) accumulating in the epidermal layer and (h) in vascular parenchyma cells. Images from Jain *et al*. (2015, used with permission). (i, j) Medullary bundles. (i) Typical eustele ring in *Portulaca hamelimoides* (Portulacaceae) (vascular tissue is dyed dark) compared to (j) medullary bundles scattered through the pith in *Mirabilis albida* (Nyctaginaceae). Images from Cunha Neto *et al*. (2024, used with permission).

Many Caryophyllales possess traits associated with drought tolerance. The most obvious is succulence, which is prevalent in families including Cactaceae, Aizoaceae, Nyctaginaceae, and Amaranthaceae (Walker *et al*., 2018; Yang *et al*., 2018; Vásquez‐Cruz *et al*., 2025; Fig. 1b–e). Aside from succulence, Caryophyllales are unique among flowering plants in that several lineages have independently evolved betalain pigmentation. In these lineages, betalain pigmentation replaces anthocyanins, which are found throughout the rest of flowering plants, including in the Caryophyllales lineages that do not possess betalains (Bischoff, 1876; Mabry, 1980; Sheehan *et al*., 2020; Walker‐Hale *et al*., 2025; Fig. 1f–h). Apart from canonical roles in pollinator and disperser attraction, the ecological function of betalains is unclear, but they accumulate in various above ground tissues in response to abiotic stressors such as high light (Vogt *et al*., 1999; Ibdah *et al*., 2002) and drought (Marchesini *et al*., 2014), and have been shown to confer stress tolerance via photoprotection under salt stress (Jain *et al*., 2015; Zhou *et al*., 2021), a mechanism that could also be significant during drought. These benefits are likely to be similar to those conferred by anthocyanins (Steyn *et al*., 2002; Jain & Gould, 2015). However, additional potential benefits of betalains relative to anthocyanins may include their association with vacuolar accumulation of Na^+^, enabling greater control of water potential gradients at times of drought or salt stress (Wang *et al*., 2007); and greater stability across a range of pHs, which may vary when plants are under abiotic stress (Strack *et al*., 2003). Together, these characteristics of betalains suggest there may be a link between betalains and the prevalence of drought‐tolerant species throughout Caryophyllales.

Caryophyllales are also noteworthy in being the only eudicot clade in which several lineages have ‘medullary bundles’, where vascular bundles occur throughout the pith rather than being constrained to the eustele ring around the edge of the pith (De Buhr, 1977; Cunha Neto *et al*., 2024; Fig. 1i,j). Medullary bundles facilitate increased water and nutrient transport throughout a plant's lifespan, potentially leading to higher seed production. They are also implicated in the growth of storage organs such as tubers or succulent stems (Mauseth, 1993; Cunha Neto *et al*., 2024). These characteristics suggest links between the evolution of medullary bundles and drought tolerance.

Due to its significant species diversity in arid climates world‐wide and the distinctive traits potentially linked to drought tolerance in many species, Caryophyllales may offer significant insights into the evolution of drought tolerance in flowering plants. We therefore address the following questions: (1) Is the evolution of drought tolerance associated with the origin of evolutionary novelties in Caryophyllales (i.e. betalains, succulence, medullary bundles)? (2) If several of these traits are associated with adaptation to drought, to what extent is the evolution of these traits correlated?

To address these questions, we analysed transcriptomic, GenBank, and morphological data comprising > 4400 species and estimated a new dated phylogenetic tree for Caryophyllales. The morphological matrix enabled a ‘tip‐dating’ approach that accounted for uncertainty in the phylogenetic placement of extant species and fossils, as well as uncertainty in the ages of fossils. Species in the dated phylogenetic tree were scored for drought tolerance, betalain pigmentation, succulence, and medullary bundles. Overall, the dated phylogenetic tree and trait data provide the necessary foundation to investigate the evolution of drought tolerance and key evolutionary novelties in Caryophyllales whilst accounting for phylogenetic and age‐related uncertainty.

## Materials and Methods

### Sequence data

Orthologs derived from the phylotranscriptomic analysis of Walker *et al*. (2018) were used. Orthologous loci were retained given two criteria: (1) the ortholog tree has at least 200 tips and (2) at least 20% of bipartitions are congruent between the ortholog tree and either the RAxML (Stamatakis, 2014) or ASTRAL (Mirarab *et al*., 2014) species tree estimated by Walker *et al*. (2018). Ortholog trees that were more congruent with the RAxML or ASTRAL trees were prioritised because topological conflict leads to errors in downstream divergence time analyses (Smith *et al*., 2018; Carruthers *et al*., 2022). Of the original dataset, 26 and 33 loci met these criteria for the RAxML and ASTRAL species trees, respectively. These two sets of loci were used in subsequent analyses.

### Palaeontological and morphological data for phylogenetic and dating analyses

We extensively surveyed the fossil literature for Caryophyllales to identify the oldest fossils attributed to major lineages across the order. From the large set of compiled fossil occurrences and taxa, we selected 14 fossil taxa (Table 1) for use in subsequent dating analyses that met the following criteria: (1) They had been subjected to careful systematic study and confidently placed at the family level (stem or crown); (2) the fossils corresponding to the taxon were sufficiently well preserved to score traits (see subsequently) for the given organ(s) represented; (3) they represented the oldest fossil evidence available for a given lineage also meeting criteria (1) and (2).

**Table 1:** Summary of fossils used in phylogenetic analyses.

Name	Family	Organ	Stratum	Age (Ma)	References
*Magnaperiporites spinosus*	Nyctaginaceae	Pollen	Eocene (Ypresian)	56–47.8	Gonzalez‐Guzman (1967); Beueher (1975); Muller (1981)
*Lymingtonia* sp.	Nyctaginaceae	Pollen	Eocene (Ypresian)	56–47.8	Mildenhall (1980); Muller (1981)
*Caryophylloflora paleogenica*	Caryophyllaceae	Inflorescence	Eocene (Bartonian/Priabonian)	41.2–33.9	Jordan & Macphail (2003)
*Periporopollenites polyoratus*	Caryophyllaceae	Pollen	Cretaceous (Campanian)	83.6–72.1	Stover & Partridge (1973); Raine (1984)
*Caryophyllidites* spp.	Caryophyllaceae	Pollen	Eocene (Bartonian)	38.8–37.7	Hoorn *et al*. (2012); Grímsson *et al*. (2020)
*Polygonocarpum curtisii*	Polygonaceae	Fruits	Palaeocene (Thanetian)	59.2–56	Manchester & O'Leary (2010)
*Polygonocarpum fimbriata*	Polygonaceae	Fruits	Oligocene	33.9–23.03	Weyland (1937); Manchester & O'Leary (2010)
*Polygonocarpum johnsonii*	Polygonaceae	Fruits	Cretaceous (Maastrichtian)	72.1–66	Manchester & O'Leary (2010)
*Persicarioipollis* spp.	Polygonaceae	Pollen	Palaeocene (Selandian)	61.6–59.2	Krutzseh (1962, 1970); Muller (1981); Song (2000); Song *et al*. (2004)
*Retitricolpites multibaculates*	Nyctaginaceae	Pollen	Cretaceous (Campanian)	83.6–72.1	Takahashi (1997)
*Chenopodipollis* sp.	Amaranthaceae/Chenopodiacea	Pollen	Cretaceous (Coniacian, Santonian)	89.8–83.6	Song *et al*. (1981, 2004); Gao *et al*. (1999)
*Aldrovanda praevesiculosa*	Droseraceae	Seeds	Oligocene	33.9–23.03	Kirchheimer (1941); Palamarev (1970); Friis (1985)
*Aldrovanda* sp.	Droseraceae	Pollen	Palaeocene	66.0–56.0	Krutzseh (1970)
*Aldrovanda unica*	Droseraceae	Pollen	Eocene	56–33.9	Kondratiev (1973)

For the 14 fossils and all extant species in the transcriptomic dataset, we scored 50 phenotypic traits. This would allow us to include the fossils as tips in the phylogenetic tree and employ a ‘tip‐dating’ approach (see next section). The traits and their corresponding states were developed to include: (1) well‐documented traits unique to Caryophyllales or lineages within; (2) major traits of known ecological significance in the order; and (3) traits that maximise the inclusion of phenotypic data available in the fossil record. Traits were scored from the literature. In the case of extant species, we obtained trait data from floras (e.g. Flora of North America Editorial Committee, 1993), taxonomic treatments of broad (e.g. Kub*itzki et al*., 2013) and narrow (e.g. Bogle, 1974) focus, and papers focused on describing the morphology or anatomy of particular organ types (e.g. Nowicke, 1975). In the case of fossil species, trait data were gathered from the paleobotanical literature (Table 1). The scoring scheme for the trait data is available in Table S2, which shows the complete list of traits, their states, and the scored value for each taxon (when relevant).

### Phylogenetic analysis of transcriptomic and morphological data

The two sets of loci were each concatenated with the 50 morphological characters, generating two matrices comprising either 26 or 33 loci from the transcriptomic data, plus the 50 morphological characters. For each matrix, the transcriptomic data were partitioned by locus, and the morphological data were partitioned according to the number of character states for each character. For example, all binary traits were in one partition, all traits with three states were in another partition, and so on (Mulvey *et al*., 2025). This resulted in eight morphological partitions.

These partitioned matrices were analysed in raxml v.8.2 (Stamatakis, 2014). A GTR + F model was used for each molecular partition, and an Mk model for each morphological partition (this was strongly favoured over a GTR model when comparing AICs). The CAT approximation for rate heterogeneity was used across all partitions. Constraint trees were used that were based on either the RAxML or ASTRAL topology from Walker *et al*. (2018), depending on the topology used to select the loci in the matrix. Constraint trees also constrained fossil tips to remain within their respective families (this included allowing fossils to branch from the stem lineage of their family). These constraint trees are justified on the basis of being underpinned by strong genomic evidence, and by the fact that the fossils included have been carefully studied and confidently placed at the family level. A maximum‐likelihood (ML) tree and 500 bootstrap (BS) trees were estimated from each matrix.

### Divergence time estimation in the phylogenetic tree estimated from transcriptomic and morphological data

To estimate divergence times, the two ML trees and each set of 500 BS trees were analysed using penalised likelihood implemented in *HMS Friday* (https://git.sr.ht/~hms/friday). *HMS Friday* is an extension of *treePL* (Smith & O'Meara, 2012) that allows for non‐extant tips (as required for tip‐dating). *r8s* (Sanderson, 2002) allowed tip dates, but *treePL* never had this functionality. In both ML trees, the *Persicariopolis* (Polygonaceae) and *Lymingtonia* (Nyctaginaceae) pollen fossils had tip‐branch lengths more than 20 times the average tip length in each tree. They were subsequently pruned from each ML tree and all BS trees. Any tip that was over 20 times the average tip length in any BS tree was also pruned. For each ML tree, dates for fossil tips were specified as the mid‐point of the stratigraphic range from which each fossil was sampled. The root node age constraint was 106.765 Ma, which corresponds to the mid‐point of the confidence interval for the Caryophyllales crown node estimated by Magallón *et al*. (2015). This value is somewhat younger than age estimates for the Caryophyllales crown node in more recent studies (e.g. Ramírez‐Barahona *et al*., 2020; Zuntini *et al*., 2024), because in Magallón *et al*. (2015) age estimates are constrained to be closer to the ages of known fossils. This does not necessarily mean the Magallón *et al*. (2015) estimates are less reliable (Smith & Beaulieu, 2024). For each BS tree, dates for fossil tips were determined by sampling uniformly between the minimum and maximum age of the stratigraphic range from which the fossil was sampled, and the root node constraint was determined by sampling uniformly from the confidence interval for the Caryophyllales crown node from Magallón *et al*. (2015). The optimal smoothing value was determined by performing cross‐validation with each ML tree. The initial search for the optimal smoothing value trialled numbers of increasing orders of magnitude from 0.01 to 1000. One hundred had the lowest *X* ^2^ and 10 the second lowest for each ML tree. A further search was then undertaken on values between 10 and 100 (at intervals of 10). Following this second search, 30 was the optimal smoothing value for both ML trees, and was used when dating all ML and BS trees. Confidence intervals for node ages were calculated as the interval between the 2.5th and 97.5th percentile of age estimates for that node across all bootstraps.

In order to assess how the tip‐calibration approach set out here compares to node calibration, an additional divergence time analysis was performed in *treePL* (Smith & O'Meara, 2012) where fossils were instead used to define minimum‐age constraints for node calibrations. Specifically, the oldest fossil that could confidently be assigned to a given clade was used as a minimum‐age constraint for the stem node of the clade (Table S3; Methods S1). Placing the minimum‐age constraint at the stem node of the clade is more conservative than placing the constraint at the crown node of the clade. It is justified on the basis that if the minimum‐age constraint were placed at the crown node, the true crown node age could in theory be younger than the minimum constraint, assuming that synapomorphies for a clade evolve along the stem branch of the clade. The root node was calibrated in the same manner as for the tip‐calibration analysis. This additional analysis was repeated on the ML tree and all bootstrap trees based on the RAxML topology of Walker *et al*. (2018). A smoothing value of 30 was used.

### Species‐level sequence data

Species‐level data for phylogenetic inference were taken from Smith *et al*. (2018). The matrix contains *ITS*, *matK*, *ndhF*, *phyC, rbcL, trnH‐psbA*, and *trnL‐trnF*. The original alignment containing 5018 taxa was used. However, additional filtering was performed: species belonging to families in the transcriptomic data were removed if > 97.5% of their aligned sequence was gaps; for species belonging to families not sampled in the transcriptomic data, the entire family was removed if all samples for that family had over 90% gaps. This led to the removal of Barbeuiaceae. Species names were then reconciled with Plants of the World Online (POWO) in January 2025, resulting in an alignment with 4341 unique taxa. Overall, this dataset provides a comprehensive foundation for analysing broad‐scale patterns of trait evolution across Caryophyllales.

### Phylogenetic inference of species‐level data

The concatenated species‐level matrix was partitioned by locus and analysed in raxml v.8.2. A GTR + CAT+F model was used for each partition. The two transcriptomic trees with morphologically placed fossils (constrained to either the RAxML or ASTRAL topology of Walker *et al*., 2018) were used as constraint trees in two separate runs. Clades with a bootstrap support value below 100 were collapsed in the constraint trees. An ML tree and 100 BS trees were estimated for analyses using each constraint tree. Overall, this approach enables estimation of species‐level phylogenetic trees whilst utilising information about relationships from the transcriptomic data, and accounting for topological and branch‐length uncertainty.

### Divergence time estimation in species‐level dataset

Divergence times were estimated on the ML and BS trees. A single tip, *Coelanthum semiquinquefolium* (Hook.) Druce, had a length over 10 times the mean length for tips in both ML trees and was therefore removed from the ML trees and all BS trees before divergence time estimation. Time constraints were based on ages estimated in the transcriptome trees (with morphologically placed fossils). Specifically, for the ML species‐level trees, any node that overlapped with a node in the relevant ML dated transcriptome tree (i.e. either based on the RAxML or ASTRAL topology of Walker *et al*., 2018) was constrained to the age of the node in the dated ML transcriptome tree. Meanwhile, for the BS species‐level trees, any node that overlapped with a node in a randomly sampled dated BS transcriptome tree was constrained to the age of the node in the dated BS transcriptome tree. Divergence times were estimated in *treePL* using a smoothing value of 30 in the ML trees and all BS trees. Alternative smoothing values had a minimal impact on age estimates. This provided a basis for estimating divergence times in the species‐level dataset which were underpinned by ages estimated from the transcriptomic data and a novel tip‐dating approach.

As with the transcriptomic divergence time analysis, an additional analysis was conducted in which the fossil dataset was used directly as minimum‐age constraints for node calibrations on species‐level trees. This additional analysis used the ML tree and all bootstrap trees based on the RAxML topology of Walker *et al*. (2018) and was conducted in *treePL* with a smoothing value of 30.

### Assembly of specimen and climate data

Coordinates for species of Caryophyllales were downloaded from GBIF in October 2024. These were filtered to include only those derived from preserved herbarium specimens. Coordinates corresponding to country capitals, geographic centroids of countries, biodiversity institutions, the sea, and those with equal longitude and latitudes were also removed (Zizka *et al*., 2019). Subsequently, specimen names were reconciled with POWO in October 2024. The final filtered dataset included 1774 521 specimens, corresponding to 10 676 species (Table S4). In total, 4270 of these species overlapped with species in species‐level phylogenetic trees. We also downloaded the Köppen‐Geiger projection from Beck *et al*. (2023).

### Categorisation of drought‐tolerant species

Species were classified based on the proportion of specimens that fell inside arid regions on the Köppen‐Geiger projection (Table S1). In the main analysis, species were classified as drought tolerant if at least 75% of specimens had coordinates within arid areas (*hot desert, cold desert, hot steppe, cold steppe*). Broadly, these are areas in which annual rainfall is less than potential evapotranspiration. In a further analysis, species were classed as drought tolerant if 75% of specimens had coordinates in hot arid areas (*hot desert*, *hot steppe*). These areas combine low rainfall with very high temperatures and intense ultraviolet radiation. In a further analysis, species were classed as drought‐tolerant if 75% of specimens had coordinates inside hot arid areas or *tropical savannah. Tropical savannahs* are inherently drought‐exposed, albeit more seasonally compared to desert/steppe, and extend contiguously from areas classified as hot desert and hot steppe. Notably, the areas included within tropical savannah in the Köppen classification incorporate seasonally dry tropical forests and their constituent elements such as the succulent biome (Ringelberg *et al*., 2020). We also generated three further classifications where species were classified as drought‐tolerant if 25% of specimens had coordinates in the three areas defined above. The strict threshold (75%) is more likely to capture species specialised to drought conditions, whilst the relaxed threshold (25%) will also include species capable of inhabiting drought conditions but not necessarily specialised to them. Overall, these categorisations are designed to capture drought tolerance relating to broad‐scale climatically determined arid areas. There are other ways that arid areas could be classified. However, by focussing on the widely used Köppen classification, we use a framework that is shared with many other studies. A further point that should be highlighted about this classification is that it will overlook more local‐scale causes of extreme water limitation, such as sandy/rocky soils in coastal or mountainous areas.

### Assembly of data for traits potentially associated with drought tolerance

For succulence, an initial classification was performed using POWO, with growth‐form descriptions downloaded in October 2024. This left considerable ambiguity, with many plants classified as shrubs that were also highly succulent. This dataset was augmented by referring to regional floras and images in online repositories (Table S5). The final dataset included 4259 species in the species‐level phylogenetic trees. Scoring succulence is incredibly complex, and to some degree subjective. The succulence classification presented here is not intended to be more authoritative than other classifications for individual Caryophyllales families (e.g. Thompson *et al*., 2024) or the entire order (Vásquez‐Cruz *et al*., 2025). Regardless, the succulence classification presented here is very similar to the Caryophyllales succulence dataset of Vásquez‐Cruz *et al*. (2025), although Vásquez‐Cruz *et al*. (2025) include both a ‘relaxed’ (which additionally includes plants with partially fleshy leaves as succulent) and ‘strict’ definition. Analyses in this study are repeated using the dataset assembled here and the two datasets from Vásquez‐Cruz *et al*. (2025).

For betalain pigmentation, the genus‐level dataset of Sheehan *et al*. (2020) was used (Table S6). As with that study, betalain pigmentation status was assumed to be consistent within genera (there are no recorded cases where it is not consistent). This resulted in betalain pigmentation data for 2926 species in the species‐level phylogenetic trees. For medullary bundles, the dataset assembled by Cunha Neto *et al*. (2024) was used. This resulted in medullary bundle data for 591 species in the species‐level phylogenetic trees (Table S7).

### Reconstruction of drought tolerance and traits potentially associated with drought tolerance

Ancestral states for each trait (succulence, betalain pigmentation, and medullary bundles) and drought tolerance were reconstructed on the species‐level trees. Tips without trait or drought tolerance data were treated as unknown. AIC values for an equal‐rates (ER) and all‐rates‐different (ARD) model were compared in each case on both ML species‐level trees (Tables S8, S9). For betalains, a model in which transitions from present to absent were excluded was tested alongside the ER and ARD models, reflecting the hypothesis (supported by in‐depth metabolic and gene function analyses in Sheehan *et al*., 2020) that betalains have been repeatedly gained but not lost in Caryophyllales. AIC values for each model were generated with the *corHMM* function from the corhmm R package (Beaulieu *et al*., 2013). Marginal likelihood node states were estimated across all bootstrap trees using the *corHMM* function and the best‐fitting model.

Analyses of correlated evolution between traits and drought tolerance categories also used the *corHMM* function from the corhmm R package. This package implements models that test for dependency between traits with respect to transition rates whilst effectively accounting for false correlations and can also account for heterogeneity in transition rates between different lineages (Boyko & Beaulieu, 2023). For these analyses, species with unknown states were removed owing to the analytical complexity of incorporating unknown states when analysing correlated evolution in large phylogenetic trees. Specifically, in provisional analyses, incorporation of species with unknown states significantly increased computational time and led to unstable results.

For analyses of correlated evolution (further explanation in Methods S2), a set of simple models was first tested for each pair of traits/drought tolerance. This included independent models, where the transition rate into and out of a state for one character is independent of the state of the other character (Fig. 2a), and a range of different dependent models. This included dependent models where: all transitions (i.e. transitions into and out of each state for each character) are dependent on the state of the other character (Fig. 2b); transitions for only one of the characters are dependent on the state of the state of the other character (but not vice versa) (Fig. 2c); only the rate of transitions in one direction is dependent on the state of the other character (Fig. 2d).

**Fig. 2:**
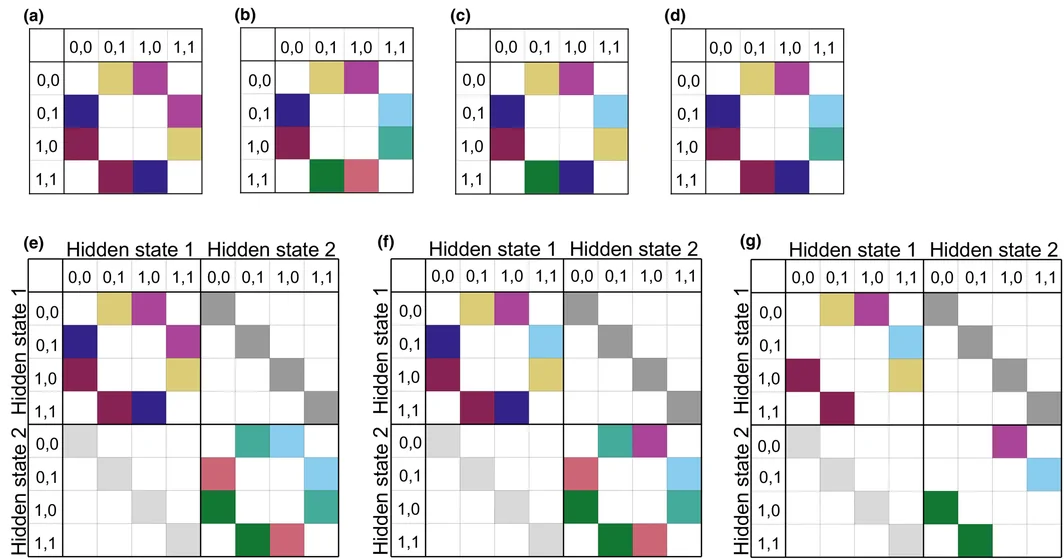
Transition matrices summarising different models of correlated evolution. Numbers labelling columns and rows (i.e. 1,0) correspond to the state of each character (e.g. 1,0 is state 1 for character 1 and state 0 for character 2). The same colour for a given transition indicates where transitions have the same rate. Grey squares indicate transitions between different hidden states, with different shades indicating how the rate from hidden state 1 to hidden state 2 differs from the rate for hidden state 2 to hidden state 1. (a) An independent model with no hidden states. Note how transitions have the same rate regardless of the state of the other character; for example, both transitions for character 1 into state 1 are pink. (b) A dependent model where the rate for every transition is dependent on the state of the other character; for example, transitions for character 1 into state 1 differ depending on the state of character 2. (c) Only transition rates for character 1 are dependent on the state of the other character. (d) Transitions from 0 to 1 are dependent on the state of the other character, but transitions from 1 to 0 are not. (e) A model with two hidden states where all rate heterogeneity for the transition rates is distributed according to the hidden states. (f) A model with two hidden states where the transition rate from 0 to 1 for character 1 is dependent on character 2, but rate heterogeneity for other transitions is instead distributed according to the hidden states. (g) An example of a model tested with betalain pigmentation where shifts to betalain pigmentation (transitions to state 1 for character 2, yellow) are only permitted for one hidden state.

A series of more complex models was then tested which included hidden states for an unobserved character (Boyko & Beaulieu, 2023). This included independent models where transition rates for observed characters (i.e. betalains, succulence, medullary bundles, and drought tolerance) depended on the ‘hidden state’ of the unobserved character (Fig. 2f). Alternatively, a range of different dependent models were trialled where one or several of the transition rates for observed characters depended on the state of the other observed character, whilst at the same time, other transition rates could depend on the unobserved character. Up to three hidden states were tested (Methods S2). Dual transitions (e.g. a transition from state 0 to state 1 for both traits simultaneously) were excluded. This is necessary for testing dependent models (i.e. to compare whether the rate of transition from state 0 to state 1 is dependent on the state of another trait) relative to independent models, including independent models with rate heterogeneity distributed according to a hidden state (Boyko & Beaulieu, 2023).

All origins of betalain pigmentation likely occurred on a small number of early branches within Caryophyllales (Sheehan *et al*., 2020). When analysing betalain pigmentation with hidden states, models were included where shifts to betalain pigmentation were only permitted for one hidden state (Fig. 2g). This reflects the hypothesis that the multiple origins of betalain pigmentation on early branches are underpinned by a common evolutionary process and avoids attempting to fit models with different rates of shifts to betalain pigmentation where no such shifts exist.

## Results

### Divergence time estimates

In our main tip‐dating analyses, the Caryophyllales crown node age is estimated in the Early Cretaceous (Albian, *c*. 100–110 Ma) (Figs 3, S2). Numerous short branches are estimated within Caryophyllales in the second half of the Cretaceous (100.5–66 Ma) such that the crown node ages of Aizoaceae, Caryophyllaceae, Droseraceae, Molluginaceae, Petiveriaceae, Polygonaceae, Plumbaginaceae, and a clade comprising Amaranthaceae and Chenopodiaceae are all estimated during this timeframe (Figs 3, S2). Crown node age estimates for most other families span the Palaeogene (66.0–23.03 Ma), with the closely related Cactaceae, Portulacaceae, Anacampserotaceae, Talinaceae, and Basellaceae having crown node age estimates in the mid‐Eocene and throughout the Oligocene (41.2–23.03 Ma). The youngest family crown node age estimates are for Nepenthaceae and Frankeniaceae in the Miocene (*c*. 10–15 Ma) (Figs 3, S2). Uncertainty in age estimates for different families varied considerably, and was especially high when the family crown node was closely related to a fossil tip (Figs 3, S2), such as the crown node of Droseraceae in particular, but also Caryophyllaceae, Chenopodiaceae/Amarantheaceae, Polygonaceae, and Nyctaginaceae. Aside from divergences between major lineages such as families (as summarised in Fig. 3), extant within‐family diversity is estimated to have evolved especially recently (< 5 Ma) in Cactaceae, Aizoaceae, and some genera in Caryophyllaceae (e.g. *Dianthus*) (Figs 4, S3, S4). By contrast, extant diversity within other large families, such as Amaranthaceae or Polygonaceae, is estimated to be much older (Figs 4, S3, S4).

**Fig. 3:**
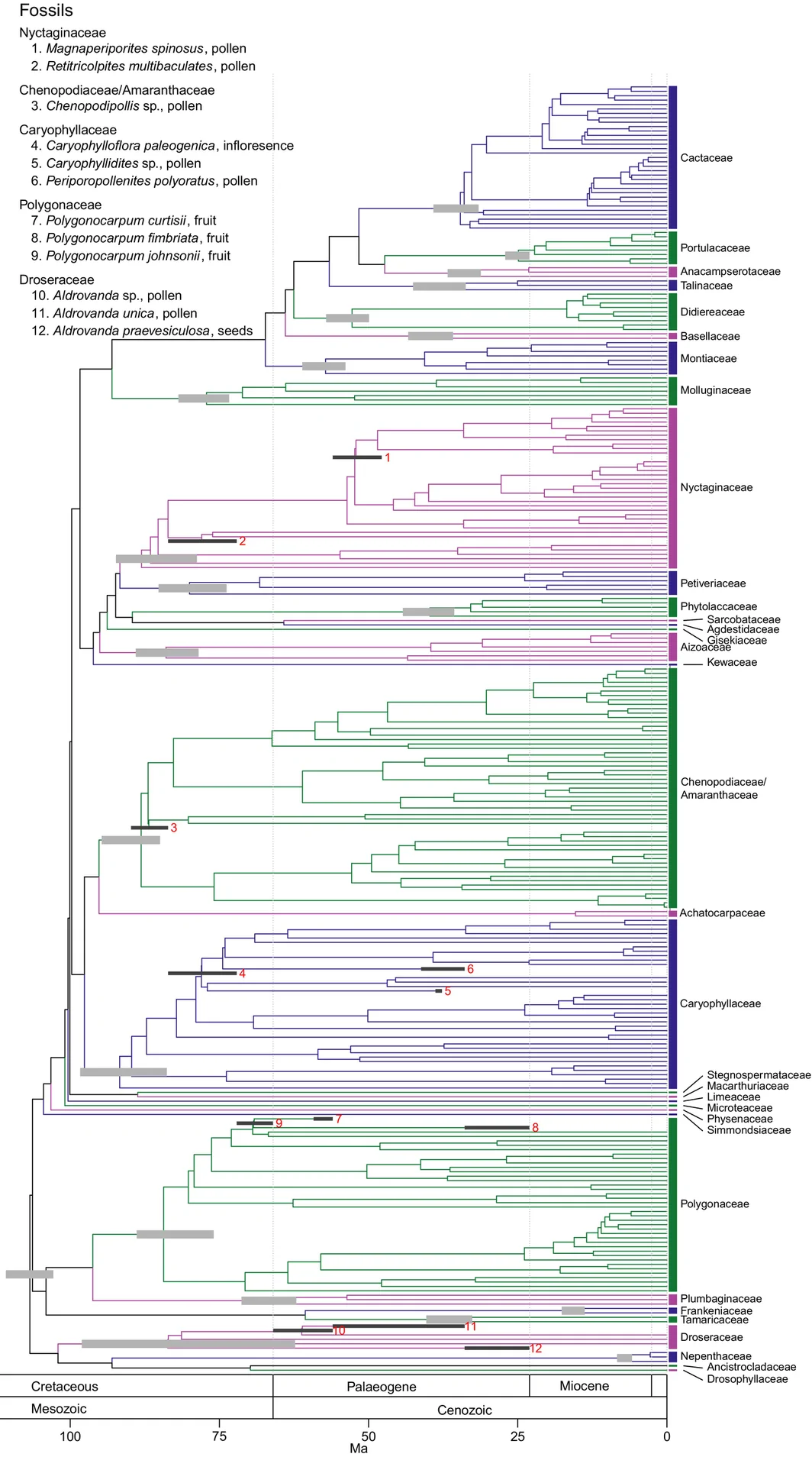
Time‐calibrated phylogenetic tree for Caryophyllales estimated from transcriptome and morphological data and constrained to the RAxML topology of Walker *et al*. (2018). Wide grey error bars represent the 95% confidence intervals for age estimates across bootstrap replicates for families. Red numbers correspond to fossil tips, with the identity of the fossil referred to at the top left. Narrow black error bars represent uncertainty in the age of each fossil. Branch colours are used to highlight different families.

**Fig. 4:**
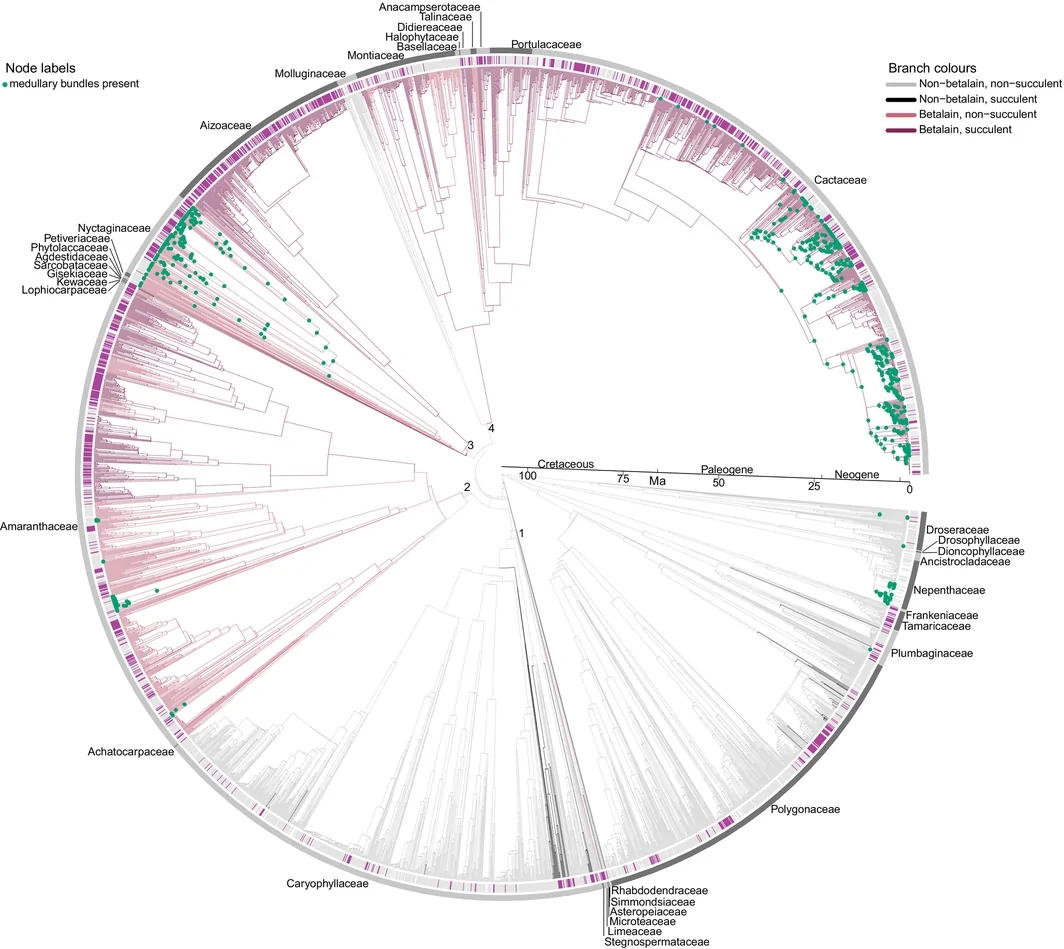
Species‐level dated phylogenetic tree estimated from data from Smith *et al*. (2018), and constrained (both topologically and temporally) to the dated transcriptome phylogenetic tree (which itself was constrained to the RAxML topology of Walker *et al*., 2018). Branch colours refer to betalain and succulence status (see key), and node labels show nodes reconstructed as possessing medullary bundles (dark green circles). Node numbers refer to the four independent gains of betalain pigmentation. Red segments in the inner ring circling the tree refer to drought‐tolerant species according to the main definition of drought tolerance.

These divergence time estimates deviate from previous studies (Magallón *et al*., 2015; Smith *et al*., 2018; Ramírez‐Barahona *et al*., 2020; Table S10). First, they have a higher level of associated uncertainty, especially compared to previous age estimates from penalised likelihood analyses (the only computationally tractable approach to divergence time estimation in a dataset of this scale). This is likely to be because our tip‐dating approach explicitly accounts for uncertainty in both the phylogenetic relationships and ages of fossils. This point is corroborated by the fact that when we use our fossil dataset as node calibrations, uncertainty in age estimates is markedly reduced (Fig. S5). Further, when we performed additional tip‐dating analyses where either Droseraceae, Caryophyllaceae, Chenopodiaceae/Amarantheaceae, Polygonaceae, and Nyctaginaceae fossils were removed (i.e. we sequentially removed fossil tips for families with very high levels of uncertainty) uncertainty in the crown node age estimate of the relevant family reduced markedly (Fig. S6). Nonetheless, we consider the relatively high uncertainty in age estimates to accurately reflect uncertainty in the fossil record. Thus, for clades with no close fossil relatives, such as Cactaceae, uncertainty is likely to be underestimated.

Compared to previous studies, the dated phylogenetic trees presented here also have somewhat shorter branches – and thus older divergence time estimates – at deeper branching positions (Fig. S5; Table S10). This is also likely to result from the novel tip‐dating approach used here. Unlike node calibration, tip‐dating explicitly accounts for the fact that fossils may belong to more nested clades. Given that the age of the fossil calibrates the dated tree, this will implicitly lead to older age estimates. Alongside this, older age estimates may also result from the older Nyctaginaceae fossils used in this study. For example, *Retitricolpites multibaculates* is from Campanian sediments (72.1–83.6 Ma, Fig. 3; Table 1), whereas the oldest Nyctaginaceae calibration used in Smith *et al*. (2018) is late Eocene (33.9–41.2 Ma).

Overall, this new dated phylogenetic framework provides the necessary context to analyse the timing and tempo of the evolution of drought tolerance and trait evolution in Caryophyllales whilst accounting for uncertainty in relationships and divergence times.

### The origin of drought‐tolerant species in Caryophyllales

For the main drought‐tolerance classification (i.e. species for which > 75% of specimens were in arid areas according to the Köppen climate classification), 1308 of the 3941 species for which an aridity status could be assessed were categorised as drought‐tolerant. For the different categorisations used, this figure varied from 546 (75% of specimens in hot arid environments) to 2156 (≥ 25% of specimens in arid environments). In all categorisations, these drought‐tolerant species are concentrated in Amaranthaceae, Nyctaginaceae, Aizoaceae, and Cactaceae (Figs 4, S7–S11), although there are species in arid conditions throughout Caryophyllales.

Reconstruction of drought tolerance suggests multiple Caryophyllales lineages evolved drought tolerance in the Cretaceous and Palaeogene, although the apparent frequency of shifts between drought tolerance and not drought tolerance leaves some uncertainty in this result (Figs S12–S17). It is also notable that where drought tolerance is classed as the ability to inhabit arid climates (i.e. ≥ 25% of specimens in arid climates), rather than being *specialised* to arid climates (≥ 75% of specimens in arid climates), transitions to drought tolerance are more often reconstructed in the Cretaceous (Figs S12–S17). As an extreme example of this, when drought tolerance is classed as 25% of specimens in any Köppen arid climate, the common ancestor of Caryophyllales is reconstructed as drought tolerant (Fig. S13). A further caveat of these reconstructions is that they cannot preclude earlier drought‐tolerant lineages in Caryophyllales that are now extinct. Alongside relatively ancient transitions to drought tolerance, much of the diversity of extant species within arid conditions, especially in Cactaceae and Aizoaceae, is estimated to have originated relatively recently (Figs 4, S7–S11).

### The evolution of betalains, succulence, and medullary bundles, and their relationship with drought tolerance

Four independent origins of betalain pigmentation are estimated within Caryophyllales, all during the Cretaceous (Figs 4, S18–S23). This result is recovered in analyses with different tree topologies and evolutionary models and is consistent with Sheehan *et al*. (2020). Drought‐tolerant species are disproportionately betalain pigmented (Figs 4, S7–S11). Using the main classification of drought tolerance, 75% of drought‐tolerant species are betalain pigmented despite only 55% of species in the dataset being betalain pigmented. Further, when the definition for drought tolerance is limited to hot arid environments, this value rises to 85%. Concordantly, there is strong support for a model in which early lineages within betalain‐pigmented clades and their immediately ancestral lineages have a far higher rate of both shifts to drought tolerance and shifts to betalain pigmentation (Figs 5a, S24–S27; Tables S11–S18). By contrast, non‐betalain‐pigmented clades have a far lower rate of shifts to drought tolerance (Figs 5a, S24–S27; Tables S11–S18).

**Fig. 5:**
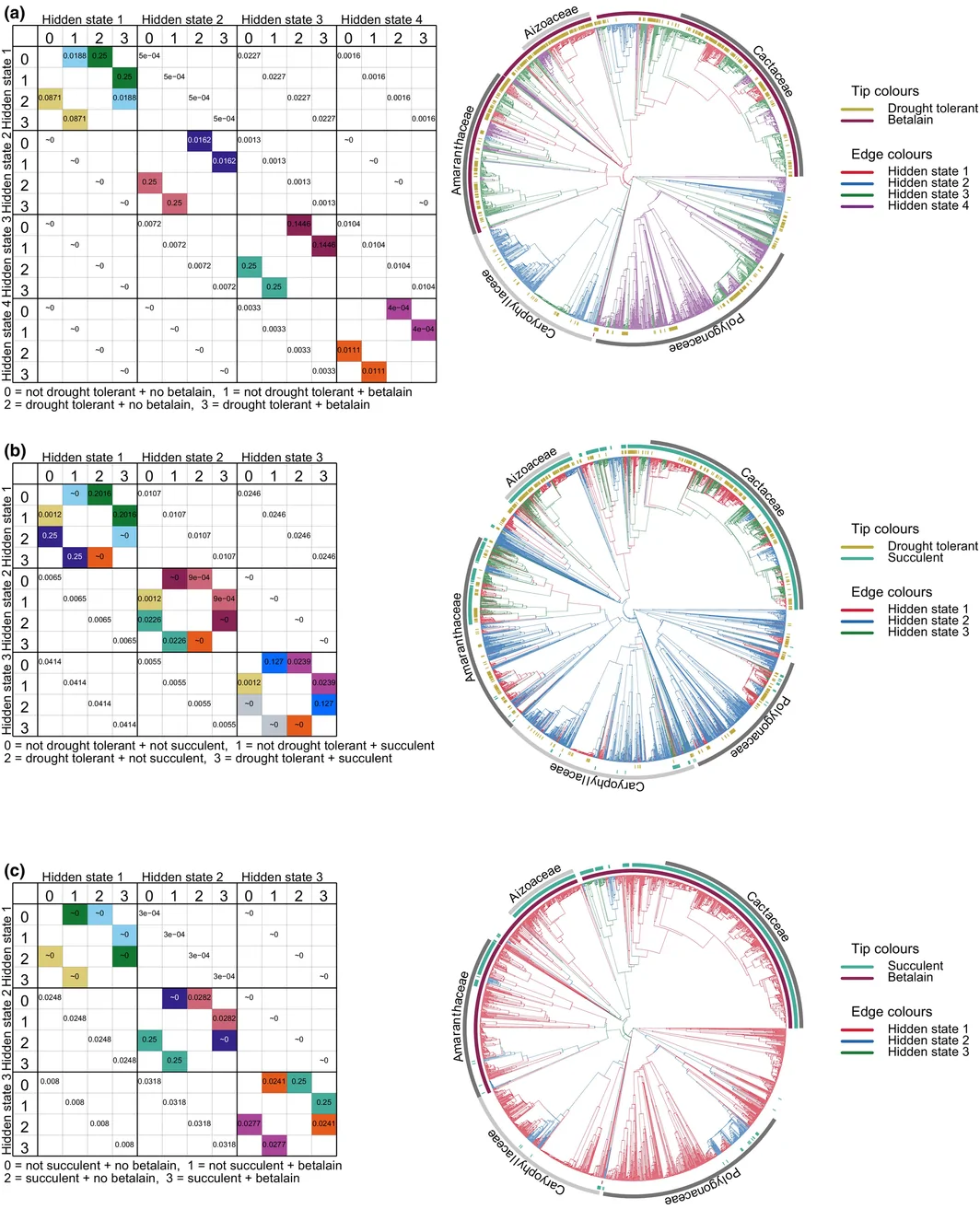
Summary of best‐fitting models used to test for correlated evolution between betalains, succulence, and drought tolerance. Transition rate matrices are shown for each combination alongside a reconstruction of how different parts of the dated phylogenetic tree evolved under each hidden state. (a) Drought tolerance and betalain pigmentation. The immediate ancestors of betalain‐pigmented lineages, and some betalain‐pigmented lineages, evolve under hidden state 1 (red branches), which has high rates of shifting into drought tolerance and shifts to betalain pigmentation. (b) Drought tolerance and succulence. All three hidden states have far higher rates of transition away from succulence when they are not drought tolerant, and there are higher rates of transition to succulence for numerous lineages in core Caryophyllales (green branches). (c) Succulence and betalains. Succulence is gained at a far higher rate on early lineages. These are the same lineages in which betalain pigmentation is being gained.

Succulence is estimated to have evolved repeatedly in the Cretaceous and the Palaeogene, and is especially prevalent in Cactacaceae, Aizoaceae, and Amaranthaceae (Figs 4, S28–S31). This timeframe is broadly consistent with reconstructions for the evolution of succulence in Thompson *et al*. (2023), although the dates presented in this study are generally older. As with betalain pigmentation, drought‐tolerant species are (unsurprisingly) predominantly succulent. For the main definition of drought tolerance, 66% of drought‐tolerant species are succulent compared to only 45% of species across the whole dataset being succulent. These patterns were broadly consistent regardless of the definition of succulence or drought tolerance used (Figs S28–S31). They are also corroborated by support for a model with the main definition of drought tolerance where the rate of loss of succulence is lower for drought‐tolerant lineages (Figs 5b, S32). For the other definitions of drought tolerance, models that lead to a positive relationship between succulence and drought tolerance were supported (Tables S19–S29).

Medullary bundles are estimated to have evolved repeatedly in the Palaeogene and the Neogene in Cactaceae, Amaranthaceae, Nyctaginaceae, and Droseraceae (Figs 4, S33, S34). This result is consistent with Cunha Neto *et al*. (2024). Unlike betalains and succulence, this trait shows no clear relationship with drought tolerance. The proportion of drought‐tolerant species with medullary bundles (*c*. 28%) is similar to the proportion with medullary bundles across the entire of Caryophyllales. There was also no support for any model implying a positive relationship between medullary bundles and drought tolerance (Fig. S35; Tables S30–S40). Further, where species were classed as drought tolerant if 75% of specimens had coordinates in hot arid areas or hot arid and tropical savannah areas, there was support for a models with higher rates of shifting away from drought tolerance if medullary bundles were present (Tables S34, S38).

### Interactions between the evolution of betalains, succulence, and medullary bundles

Most succulent species (97%) are betalain‐pigmented (Figs 4, S7–S11), with only a small number of succulent non‐betalain‐pigmented species scattered throughout Caryophyllaceae, Limeaceae, and Polygonaceae. This relationship is corroborated by support for a model where early lineages in Carypohyllales with high rates of shifting to betalain pigmentation also have high rates of shifting to succulence (Figs 5c, S36; Table S41).

Betalain pigmentation also appears to be associated with medullary bundles (although the quantity of data underlying this comparison is considerably less). 87.5% of species with medullary bundles are betalain pigmented, and there was strong support for a model in which the rate of shifting away from possessing medullary bundles was far lower for betalain‐pigmented lineages (Fig. S37; Table S42). As with other models involving betalain pigmentation, the best‐fitting model incorporated rapid rates of shifting to betalain pigmentation on early lineages within Caryophyllales (Fig. S37).

Despite succulence and medullary bundles being positively associated with betalain pigmentation, there was evidence of a negative relationship between medullary bundles and succulence. The only succulent clade with medullary bundles was Cactoideae (within Cactaceae, Fig. 4), and there was support for a model where high rates of shifting to succulence were associated with comparatively low rates of shifting to medullary bundles (Fig. S38; Table S43). Therefore, although the evolution of betalains is positively associated with the evolution of succulence and medullary bundles, these two evolutionary novelties typically appear to be mutually exclusive.

## Discussion

All four origins of betalain pigmentation occurred early within Caryophyllales. There was strong support for a model with high rates of shifting to drought tolerance and betalain pigmentation on these lineages (Fig. 5a). By contrast, the rate of shifting to drought tolerance is far lower on non‐betalain‐pigmented lineages (Fig. 5a). Drought‐tolerant lineages in Caryophyllales therefore tend to be betalain pigmented (Fig. 4). Further, almost all succulent lineages are betalain pigmented and often occur in arid environments. There was strong support for models in which transitions away from succulence were significantly lower in drought‐tolerant lineages and transitions to succulence were significantly higher on lineages in which betalain pigmentation evolves (Fig. 5b,c). Taken together, these results suggest that betalains are associated with survival in arid environments. This, in turn, may increase the adaptive advantages of succulence (a trait with many associated costs that preclude it in non‐drought environments; Von Willert *et al*., 1990; Males, 2017) and reduce the likelihood of evolutionary transitions away from this growth form. The origin of betalains (especially origins 3 and 4, Fig. 4) and succulence may also have been facilitated by the several whole‐genome duplications that occurred in early lineages within Caryophyllales (Yang *et al*., 2018).

The evolution of medullary bundles shows no correlation with drought tolerance. However, medullary bundles appear to be associated with betalain pigmentation, with the rate of shifts away from medullary bundles being lower for betalain‐pigmented lineages (Fig. S37). This suggests betalains may facilitate survival in a range of stressful conditions (beyond just drought, for example salinity), which increases the adaptive advantages of traits that are not strictly linked to drought such as medullary bundles. In this vein, it is noteworthy that betalains are likely to have originated before lineages became specialised to drought conditions (at least on extant lineages, Figs S12–S23), that is, the origin of betalains may not have been directly associated with drought tolerance but did subsequently facilitate drought tolerance.

There was also some evidence of a negative relationship between medullary bundles and succulence. The only succulent lineages that possess medullary bundles are in Cactoideae (Fig. 4), and there was partial support for a model where high rates of shifting to succulence were associated with comparatively low rates of shifting to medullary bundles (Fig. S38; Table S43). This implies that the cost associated with these traits may preclude them from co‐evolving. Overall, these findings highlight fundamental links between key evolutionary novelties in Caryophyllales and species diversity in arid environments.

### The changing face of arid environments through time

Despite the overall relationship between betalains and arid environments, the nature and extent of arid environments has shifted markedly over the > 100 Myr timescale of this study. For example, the origin of betalains and associated shifts into arid environments in the Cretaceous significantly predate the expansion of many arid habitats from the late Oligocene (Keeley & Rundel, 2005; Bobe, 2006; Jaramillo, 2023). The results presented here should therefore only be interpreted in broad terms, that is, when did Caryophyllales lineages evolve drought tolerance as a general trait, rather than when did they evolve to inhabit a specific drought‐prone habitat? Such an approach is justifiable given that large environmentally determined arid environments have undoubtedly existed from at least the mid‐Cretaceous (Hallam, 1984; Hay & Floegel, 2012) and the results we present appear to be very robust to how arid areas are defined.

### Betalains and their functional importance in arid environments

The link between arid environments and betalain pigmentation is consistent with studies showing that, like with anthocyanins, betalain production is induced by abiotic stress (Vogt *et al*., 1999; Ibdah *et al*., 2002) and confers stress tolerance (Jain *et al*., 2015; Zhou *et al*., 2021). Betalains may confer additional benefits under stress beyond those of anthocyanins given their greater stability under a range of pHs (Strack *et al*., 2003), and their association with the vacuolar accumulation of Na^+^ (Wang *et al*., 2007). Given the putative role of betalains for conferring tolerance to a range of stressors, the origin of betalains may have acted as a mechanism by which Caryophyllales could survive in a diverse set of stressful conditions, including drought. Nonetheless, the exact mechanisms by which betalains promote stress tolerance and the conditions under which betalain biosynthesis increases are somewhat unclear (e.g. Jain & Gould, 2015), and are unlikely to always correspond in a predictable way to environmental cues. Our work should therefore prompt further assessment of the ecophysiological link between betalains and abiotic stress tolerance in more experimental systems, to further determine how generalisable this relationship is.

The fact that betalains are by no means unique in promoting survival in arid environments or stressful conditions more generally should also be highlighted. Families such as Kewaceae and Molluginaceae, and several species in non‐core Caryophyllales families such as Polygonaceae or Droseraceae, are anthocyanin rather than betalain pigmented but occur in arid environments. Lineages can therefore adapt to arid environments without betalains, with anthocyanins potentially facilitating survival in stressful conditions (Jain & Gould, 2015) and enabling the evolution of novel traits (as is hypothesised in this study with betalains). For example, a group of species within the anthocyanin‐pigmented *Drosera* (Droseraceae) have adapted to arid conditions by evolving tubers from which the plant regrows after drought (Dixon & Pate, 1978; Conran, 2008).

The variety of traits that can promote survival in arid conditions may also explain why dependent models for the evolution of drought tolerance and betalain pigmentation are not supported here. With many factors potentially promoting drought tolerance (i.e. anthocyanins, succulence, and tubers), models with multiple hidden states appear to be better able to capture rate heterogeneity with regard to the gain and loss of drought tolerance. Regardless, the lack of support for a dependent model does not negate the fact that in our analyses, by far the highest rates of gaining drought tolerance was in lineages that are also gaining betalain pigmentation (Fig. 5a). By contrast, other hidden states had either very high rates of losing drought tolerance combined with low or moderate rates of gaining drought tolerance (distributed throughout the phylogenetic tree) or low rates for gaining and losing drought tolerance (especially prevalent in Polygonaceae).

The absence of anthocyanins in betalain‐pigmented lineages raises the further question of whether the ancestors of betalain‐pigmented lineages had any pigmentation (i.e. whether both anthocyanins and betalains were absent). Such a situation would not only be significant for stress tolerance, but also for pollination, given that many floral pigments are derived from anthocyanin or betalain (although we cannot rule out floral pigment regulation being separated from vegetative tissue). It has been hypothesised, therefore, that betalains originally evolved in an unpigmented ancestor and facilitated pollination (Ehrendorfer, 1976), with the regulation of betalain pigmentation in response to stress evolving subsequently. A clearer understanding of when the genes underlying the regulation of betalain pigmentation evolved would be necessary to further test this.

Together, these points highlight that the role of betalains is complex and multifaceted, and may overlap with those of other compounds such as anthocyanins. A greater understanding of the functional importance of betalains may help to address these questions.

### Succulence evolves in betalain‐pigmented lineages and appears to exclude medullary bundles

The vast majority of succulent lineages within Caryophyllales are also betalain pigmented (Fig. 4), and there was strong support for models suggesting the evolution of these two traits is linked (Fig. 5c). This is the first study to establish a relationship between these two characteristic features of Caryophyllales. Unlike betalains, succulence has been previously associated with several other traits, such as CAM photosynthesis and cell size (Winter *et al*., 1983; Males, 2017; Heyduk, 2021). This study contributes to the understanding of complex variables associated with succulence, highlighting the close link between betalain pigmentation and the evolution of diverse succulent lineages within Caryophyllales.

Both betalain biosynthesis and leaf and stem succulence involve biophysical changes to the plant vacuole (Wang *et al*., 2007; Males, 2017; Heyduk, 2021). For succulence, these changes include expansion of the plant vacuole, whilst for betalains, changes to the vacuole involve the accumulation of solutes and the accumulation of betalains themselves. The close association between succulence and betalains is, therefore, not necessarily unexpected, as biophysical adaptations may enable one trait to potentially lower the cost of evolving the other. It is perhaps also the case that by facilitating the inhabitation of arid environments, betalains enable Caryophyllales species to inhabit conditions where the significant costs associated with succulence are outweighed by its benefits. These costs of succulence include increased carbon requirements, construction of water‐storing hydrenchyma cells, and reduced photosynthetic efficiency (Von Willert *et al*., 1990; Males, 2017).

Medullary bundles are also associated with betalain pigmentation. Given that medullary bundles show no clear association with drought conditions, this finding suggests that betalains may facilitate survival in stressful conditions aside from drought, such as high salinity, temperature extremes, or exposure to herbivory. By facilitating survival in any one of these extreme conditions, the considerable costs of evolutionary novelties such as medullary bundles or succulence may be outweighed by the benefits they confer.

Medullary bundles also show some evidence of a negative association with succulence. The only lineages with medullary bundles and succulence are within Cactoideae (Fig. 4), which contains many large, columnar species with a narrow, upright growth habit. This observation, combined with the known association of medullary bundles with storage tissue and water transport (Mauseth, 1993; Cunha Neto *et al*., 2024), implies medullary bundles have played a role in the evolution of these especially large water‐storing stems. Given that outside Cactoideae medullary bundles and succulence never overlap (Fig. 4), and the support for a model where high rates of shifting to succulence were associated with comparatively low rates of shifting to medullary bundles, it may be the case that the costs associated with evolving medullary bundles preclude the evolution of succulence, and that this cost is only overcome in very rare circumstances for example columnar cacti with their unique requirements for drought tolerance and water and nutrient transport. Instead of being associated with any one particular trait, associations between medullary bundles and other traits may be diverse and context‐specific. For example, several carnivorous species in Nepenthaceae and Droseraceae possess medullary bundles, implying they may play a role in the novel modes of nutrient transport utilised by these species. Nevertheless, interpretation of any result pertaining to medullary bundles in particular should be treated with caution given the fairly limited taxon sampling for this trait.

### Conclusion

From their origins as organisms living solely in water, the ability of some land plants to inhabit the most arid conditions on Earth represents one of the most profound evolutionary changes. Here, we characterise the role of evolutionary novelties within Caryophyllales in enabling adaptation to arid environments. We highlight that multiple origins of betalain pigmentation may have been particularly important, facilitating the survival of early Caryophyllales lineages in arid conditions and increasing the adaptive advantages of succulence. This is the first study to draw such links between these evolutionary novelties and the extensive diversity of Caryophyllales in arid environments. Clearly, further analyses are needed that more explicitly account for the varied ecological characteristics of different arid ecosystems, breaking down the broad categorisations used here. Further, a greater understanding of the functional importance of the evolutionary novelties in Caryophyllales, especially betalains, is required for a more fine‐grained understanding of their role in drought tolerance in particular, and stress tolerance more generally. Aside from trait evolution and drought tolerance, this study also generates a new dated phylogenetic tree for Caryophyllales using a novel tip‐dating approach that more effectively incorporates uncertainty compared to previous studies. Despite the benefits of this approach in Caryophyllales, this approach may not be feasible in many groups with poorer fossil records, and is only at its most effective within a few specific clades within this study (i.e. some clades in this study have far fewer fossils than others, with clades with few fossils having less uncertainty). Further, this method will still be undermined by unaccounted for rate variation. These limitations are important given the fundamental dependence of evolutionary analyses on accurate divergence time estimates.

## Competing interests

None declared.

## Author contributions

TC, SAS, and NW‐H designed the study. GWS assembled fossil data. TC performed analyses. TC wrote the manuscript. SAS, NW‐H, GWS, SFB, HAM, YY, and TC reviewed and contributed to the manuscript.

## Disclaimer

The New Phytologist Foundation remains neutral with regard to jurisdictional claims in maps and in any institutional affiliations.

## Supporting information


**Fig. S1** Distribution of drought‐tolerant species.
**Fig. S2** Dated phylogenetic tree constrained to ASTRAL topology.
**Fig. S3** Dated species‐level phylogenetic tree constrained to the RAxML topology.
**Fig. S4** Dated species‐level phylogenetic tree constrained to the ASTRAL topology.
**Fig. S5** Lineages through time plots for dated phylogenetic trees.
**Fig. S6** Comparison of 95% CI widths for families when either all fossils are included or fossils for the relevant family are excluded.
**Fig. S7** Species‐level phylogenetic tree with key traits reconstructed and drought tolerance defined where at least 25% of specimens are in arid climates.
**Fig. S8** Species‐level phylogenetic tree with key traits reconstructed and drought tolerance defined where at least 75% of specimens are in hot arid climates.
**Fig. S9** Species‐level phylogenetic tree with key traits reconstructed and drought tolerance defined where at least 25% of specimens are in hot arid climates.
**Fig. S10** Species‐level phylogenetic tree with key traits reconstructed and drought tolerance defined where at least 75% of specimens are in hot arid climates or hot savannah.
**Fig. S11** Species‐level phylogenetic tree with key traits reconstructed and drought tolerance defined where at least 25% of specimens are in hot arid climates or hot savannah.
**Fig. S12** Reconstruction of drought tolerance with the best‐fitting model (ARD). Drought tolerance defined where at least 75% of specimens are in arid climates.
**Fig. S13** Reconstruction of drought tolerance with the best‐fitting model (ER). Drought tolerance defined where at least 25% of specimens are in arid climates.
**Fig. S14** Reconstruction of drought tolerance with the best‐fitting model (ARD). Drought tolerance defined where at least 75% of specimens are in hot arid climates.
**Fig. S15** Reconstruction of drought tolerance with the best‐fitting model (ARD). Drought tolerance defined where at least 25% of specimens are in hot arid climates.
**Fig. S16** Reconstruction of drought tolerance with the best‐fitting model (ARD). Drought tolerance defined where at least 75% of specimens are in hot arid climates or hot savannah.
**Fig. S17** Reconstruction of drought tolerance with the best‐fitting model (ARD). Drought tolerance defined where at least 25% of specimens are in hot arid climates or hot savannah.
**Fig. S18** Reconstruction of betalain pigmentation with an irreversible model on the species‐level phylogenetic tree constrained to the RAxML topology.
**Fig. S19** Reconstruction of betalain pigmentation with an irreversible model on the species‐level phylogenetic tree constrained to the ASTRAL topology.
**Fig. S20** Reconstruction of betalain pigmentation with an ER model on the species‐level phylogenetic tree constrained to the RAxML topology.
**Fig. S21** Reconstruction of betalain pigmentation with an ER model on the species‐level phylogenetic tree constrained to the ASTRAL topology.
**Fig. S22** Reconstruction of betalain pigmentation with an ARD model on the species‐level phylogenetic tree constrained to the RAxML topology.
**Fig. S23** Reconstruction of betalain pigmentation with an ARD model on the species‐level phylogenetic tree constrained to the ASTRAL topology.
**Fig. S24** Best‐fitting model for evolution of betalain pigmentation and drought tolerance (defined as at least 75% of specimens in arid climates).
**Fig. S25** Best‐fitting model for evolution of betalain pigmentation and drought tolerance (defined as at least 25% of specimens in arid climates).
**Fig. S26** Best‐fitting model for evolution of betalain pigmentation and drought tolerance (defined as at least 75% of specimens in hot arid climates).
**Fig. S27** Best‐fitting model for evolution of betalain pigmentation and drought tolerance (defined as at least 75% of specimens in hot arid climates or hot savannah).
**Fig. S28** Reconstruction of succulence using the best‐fitting model (ARD) on the species‐level phylogenetic tree constrained to the RAxML topology.
**Fig. S29** Reconstruction of succulence using the best‐fitting model (ARD) on the species‐level phylogenetic tree constrained to the ASTRAL topology.
**Fig. S30** Reconstruction of succulence using the best‐fitting model (ER) on the species‐level phylogenetic tree constrained to the RAxML topology. This uses the relaxed definition of succulence from Vásquez‐Cruz *et al*. (2025).
**Fig. S31** Reconstruction of succulence using the best‐fitting model (ER) on the species‐level phylogenetic tree constrained to the RAxML topology. This uses the strict definition of succulence from Vásquez‐Cruz *et al*. (2025).
**Fig. S32** Best‐fitting model for the evolution of succulence and drought tolerance (defined as at least 75% of specimens in arid climates).
**Fig. S33** Reconstruction of medullary bundles using the best‐fitting model (ARD) on the species‐level phylogenetic tree constrained to the RAxML topology.
**Fig. S34** Reconstruction of medullary bundles using the best‐fitting model (ARD) on the species‐level phylogenetic tree constrained to the ASTRAL topology.
**Fig. S35** Best‐fitting model for the evolution of medullary bundles and drought tolerance (defined as at least 75% of specimens in arid climates).
**Fig. S36** Best‐fitting model for the evolution of succulence and betalains.
**Fig. S37** Best‐fitting model for the evolution of medullary bundles and betalains.
**Fig. S38** Best‐fitting model for the evolution of medullary bundles and succulence.
**Methods S1** Additional justification of node calibrations.
**Methods S2** Additional justification of independent and dependent trait evolution models.

**Table S1** Caryophyllales species matched to Köppen climate classification.

**Table S2** Morphological trait data used in phylogenetic and tip‐dating analyses.
**Table S3** Summary of node calibrations.

**Table S4** Full specimen dataset.

**Table S5** Succulence dataset.

**Table S6** Betalain dataset.

**Table S7** Medullary bundle dataset.
**Table S8** Model comparison for single trait ancestral state reconstructions on trees constrained to RAxML topology.
**Table S9** Model comparison for single trait ancestral state reconstructions on trees constrained to ASTRAL topology.
**Table S10** Comparison of family stem age estimates.
**Table S11** Models for betalain pigmentation and drought tolerance (75% of specimens in arid areas).
**Table S12** Models for betalain pigmentation and drought tolerance (25% of specimens in arid areas).
**Table S13** Models for betalain pigmentation and drought tolerance (75% of specimens in hot arid areas).
**Table S14** Models for betalain pigmentation and drought tolerance (25% of specimens in hot arid areas).
**Table S15** Rate estimates for betalain pigmentation and drought tolerance (25% of specimens in hot arid areas).
**Table S16** Models for betalain pigmentation and drought tolerance (75% of specimens in hot arid areas and tropical savannah).
**Table S17** Models for betalain pigmentation and drought tolerance (25% of specimens in hot arid areas and tropical savannah).
**Table S18** Rate estimates for betalain pigmentation and drought tolerance (25% of specimens in hot arid areas and tropical savannah).
**Table S19** Models for succulence and drought tolerance (75% of specimens in arid areas).
**Table S20** Models for succulence and drought tolerance (25% of specimens in arid areas).
**Table S21** Rate estimates for succulence and drought tolerance (25% of specimens in arid areas).
**Table S22** Models for succulence and drought tolerance (75% of specimens in hot arid areas).
**Table S23** Rate estimates for succulence and drought tolerance (75% of specimens in hot arid areas).
**Table S24** Models for succulence and drought tolerance (25% of specimens in hot arid areas).
**Table S25** Rate estimates for succulence and drought tolerance (25% of specimens in hot arid areas).
**Table S26** Models for succulence and drought tolerance (75% of specimens in hot arid areas or tropical savannah).
**Table S27** Rate estimates for succulence and drought tolerance (75% of specimens in hot arid areas or tropical savannah).
**Table S28** Models for succulence and drought tolerance (25% of specimens in hot arid areas or tropical savannah).
**Table S29** Rate estimates for succulence and drought tolerance (25% of specimens in hot arid areas or tropical savannah).
**Table S30** Models for medullary bundles and drought tolerance (75% of specimens in arid areas).
**Table S31** Models for medullary bundles and drought tolerance (25% of specimens in arid areas).
**Table S32** Rate estimates for medullary bundles and drought tolerance (25% of specimens in arid areas).
**Table S33** Models for medullary bundles and drought tolerance (75% of specimens in hot arid areas).
**Table S34** Rate estimates for medullary bundles and drought tolerance (75% of specimens in hot arid areas).
**Table S35** Models for medullary bundles and drought tolerance (25% of specimens in hot arid areas).
**Table S36** Rate estimates for medullary bundles and drought tolerance (25% specimens in hot arid areas).
**Table S37** Models for medullary bundles and drought tolerance (75% of specimens in hot arid areas or tropical savannah).
**Table S38** Rate estimates for medullary bundles and drought tolerance (75% of specimens in hot arid areas or tropical savannah).
**Table S39** Models for medullary bundles and drought tolerance (25% of specimens in hot arid areas or tropical savannah).
**Table S40** Rate estimates for medullary bundles and drought tolerance (25% of specimens in hot arid areas or tropical savannah).
**Table S41** Models for succulence and betalains.
**Table S42** Models for medullary bundles and betalains.
**Table S43** Models for medullary bundles and succulence.
Please note: Wiley is not responsible for the content or functionality of any Supporting Information supplied by the authors. Any queries (other than missing material) should be directed to the *New Phytologist* Central Office.

## Data Availability

Code and analysis outputs and Tables [Link], [Link], [Link], [Link] are available on Zenodo doi: 10.5281/zenodo.20415595.
