# Supplementary material for: Evolution in arid environments: the role of betalains, succulence, and medullary bundles
Source: New Phytol. 2026 Sep 3;252(3):1250–68. doi: 10.1111/nph.71521 (PMC13628552; doi:10.1111/nph.71521)
Supplement: Supplementary file 1 [file NPH-252-1250-s006.pdf]

New Phytologist Supporting Information

Article title: Evolution in arid environments: the role of betalains, succulence, and medullary bundles

Authors: Tom Carruthers, Samuel F Brockington, Hiroshi A Maeda, Gregory W Stull, Nathanael Walker-Hale, Ya Yang, Stephen A Smith

Article acceptance date: 28 July 2026

Method S1: For node calibration analyses, we took the oldest fossil assigned to each family (or in the case of *Magnaperiporites spinosus* the Nyctagineae tribe) in the constraint tree for the tip-dating analysis. For the species level analysis, an additional maximum constraint was used at the Cactaceae crown node. This is a secondary calibration from Arakaki et al (2011), and is also used as a maximum constraint by Smith et al. (2018). Arakaki et al. (2011) generate this constraint from an age estimate for Cactaceae that was obtained in a broader analysis spanning seed plants. Additional analysis were performed where this constraint was excluded. In this case, crown age estimates for Cactaceae spanned into the Cretaceous.

Method S2: If there was no evidence of dependency between observed traits with two hidden states, a full set of models with three hidden states was not tested. Instead, a single additional model was tested where rates varied independently according to the hidden states.

For betalain pigmentation and arid environments, a notable feature of several models was that transitions into the hidden state with high rates of shifting to betalain pigmentation were relatively high. This seems biologically unrealistic. Shifts to betalain pigmentation occur exclusively on early lineages in Caryophyllales. It is therefore unlikely that shifts to a hidden state with a higher rate of transition to betalain pigmentation occur at a similar rate to shifts into the hidden state with lower/0 rates. This result is likely caused by heterogeneity in the rate of transitions into arid environments, and the fact that the hidden state with higher transition rates to betalain pigmentation has far higher rates of transition to arid environments. Higher rates can therefore only be accessed by switching to the hidden state with high transition rates to betalain pigmentation. Therefore, a further model was tested in which there were four hidden states and where shifts into betalain pigmentation were only permitted for one hidden state. This model often had a better AIC score and had very low transition rates into the hidden state in which transitions to betalain pigmentation are permitted. Overall, this model has very high rates of transition into the arid environments when in the hidden state that permits transitions to betalain pigmentation. This hidden state is primarily reconstructed on deep branches in the phylogenetic tree (Fig.5).

Table S1. Caryophyllales species with specimens matched to Köppen climate classification.

Table S2. Morphological trait data used in phylogenetic and tip dating analyses.

Table S3. Summary of temporal constraints used in node calibration analysis.

Name	Family	Organ	Stratum	Node	Constraint age (Ma)	Reference
Magnaperiporites spinosus	Nyctaginaceae	Pollen	Eocene (Ypresian)	Stem Nyctagineae	47.8 (Min)	González-Guzmán 1967;Beucher 1975; Muller 1981
Retitricolpites multibaculates	Nyctaginaceae	Pollen	Cretaceous (Campanian)	Stem Nyctaginaceae	72.1 (Min)	Takahashi 1997
Polygonocarpum johnsonii	Polygonaceae	Fruits	Cretaceous (Maastrichtian)	Stem Polygonaceae	66 (Min)	Manchester and O'Leary 2010
Chenopodipollis sp.	Amaranthaceae/Chenopodiacea	Pollen	Cretaceous (Coniacian, Santonian)	Stem Chenopodiaceae/Amaranthaceae	83.6 (Min)	Song et al. 1981, 2004; Gao et al. 1999;
Aldrovanda sp.	Droseraceae	Pollen	Palaeocene	Stem Droseraceae	56.0 (Min)	Krutzsch 1970
Secondary	-	-	-	Crown Cactaceae	35 (Max)*	Arakaki et al. (2011)
Secondary	-	-	-	Root node	102.53-111 (point calibration, sampling uniformly in this range)	Magallon et al. (2015)

*only used in species level analysis

Table S4. Full specimen dataset.

Table S5. Succulence dataset.

Table S6. Betalain dataset.

Table S7. Medullary bundle dataset.

Table S8. Model comparison for simple single trait ancestral state reconstructions on trees constrained to the RAxML topology of Walker et al. (2018).

Trait/Drought Category	Model	AIC	Log Likelihood
Betalain pigmentation	ER	65.26549	-31.63274
Betalain pigmentation	ARD	66.20234	-31.10117
Betalain pigmentation	Irreversible	66.50005	-31.25002
Succulence	ER	896.753	-447.3765
Succulence	ARD	891.9911	-443.9956
Succulence (relaxed, from Vásquez-Cruz et al. 2025)	ER	474.123	-236.0615
Succulence (relaxed, from Vásquez-Cruz et al. 2025)	ARD	475.5782	-235.7891
Succulence (strict, from Vásquez-Cruz et al. 2025)	ER	290.7904	-144.3952
Succulence (strict, from Vásquez-Cruz et al. 2025)	ARD	287.8027	-141.9014
Medullary bundles	ER	365.8642	-181.9321

Medullary bundles	ARD	347.3751	-171.6876
Arid 75%	ER	4202.612	-2100.306
Arid 75%	ARD	4039.089	-2017.545
Arid 25%	ER	4600.173	-2299.087
Arid 25%	ARD	4601.725	-2298.863
Hot Arid 75%	ER	3288.946	-1643.473
Hot Arid 75%	ARD	2767.557	-1381.779
Hot Arid 25%	ER	4292.178	-2145.089
Hot Arid 25%	ARD	4047.212	-2021.606
Hot Arid and Savannah 75%	ER	4039.965	-2018.982
Hot Arid and Savannah 75%	ARD	3614.675	-1805.337
Hot Arid and Savannah 25%	ER	4554.926	-2276.463
Hot Arid and Savannah 25%	ARD	4477.546	-2236.773

Table S9. Model comparison for simple single trait ancestral state reconstructions on trees constrained to the ASTRAL topology of Walker et al. (2018).

Trait/Drought Category	Model	AIC	Log Likelihood
Betalain pigmentation	ER	65.86816	-31.93408
Betalain pigmentation	ARD	66.24432	-31.12216
Betalain pigmentation	Irreversible	66.35284	-31.17642
Succulence	ER	905.8867	-451.9433
Succulence	ARD	900.979	-448.4895
Medullary bundles	ER	361.3388	-179.6694
Medullary bundles	ARD	344.531	-170.2655
Arid 75%	ER	4232.921	-2115.461
Arid 75%	ARD	4058.407	-2027.204
Arid 25%	ER	4603.677	-2300.839
Arid 25%	ARD	4605.365	-2300.682
Hot Arid 75%	ER	3286.299	-1642.149
Hot Arid 75%	ARD	2726.356	-1361.178
Hot Arid 25%	ER	4316.217	-2157.109
Hot Arid 25%	ARD	4048.952	-2022.476
Hot Arid and Savannah 75%	ER	4053.622	-2025.811
Hot Arid and Savannah 75%	ARD	3598.226	-1797.113
Hot Arid and Savannah 25%	ER	4566.704	-2282.352
Hot Arid and Savannah 25%	ARD	4469.841	-2232.92

Table S10. Comparison of family stem age estimates in this study compared to previous studies.

Family	This study	Magallon et al. 2015 BEAST	Megallon et al. 2015 treePL	Ramírez-Barahona et al. (2020)	Smith et al (2018)
Droseraceae	83.7 (94.1-105.8)	76.83 (53.46-92.33)	89.54 (85.06-92.97)	101.5 (76.6-120.9)	90.6 (63.1,92.3)
Nepenthaceae	83.7 (94.1-105.8)	76.83 (53.46-92.33)	89.54 (85.06-92.97)	90.1 (65.0-114.0)	87.2 (76.6,93.8)
Frankeniaceae	60.6 (56.2-64.8)	53.83 (28.98-74.74)	61.06 (56.67-66.38)	69.4 (42.5-97.5)	74.2 (56.9,84.7)
Tamaricaceae	60.6 (56.2-64.8)	53.83 (28.98-74.74)	61.06 (56.67-66.38)	69.4 (42.5-97.5)	84.2 (65.4,109.5)
Plumbaginaceae	96.2 (91.7-99.9)	67.9 (65.63-78.21)	66.81 (65.5-71.8)	82.1 (66.4-99.6)	102.8 (88.2,105.4)
Polygonaceae	96.2 (91.7-99.9)	67.9 (65.63-78.21)	66.81 (65.5-71.8)	82.1 (66.4-99.6)	102.8 (88.2,105.4)
Caryophyllaceae	97.6 (94.4-102.2)	70.11 (50.34-88.56)	81.27 (77.91-84.67)	96.9 (83.0-106.6)	88.7 (88.6,88.6)
Amaranthaceae	97.6 (94.4-102.2)	70.11 (50.34-88.56)	81.27 (77.91-84.67)	85.7 (74.3-98.6)	81.8 (81.8,81.8)
Aizoaceae	95.0 (90.1-98.9)	74.88 (72.5-77.87)	73.5 (72.17-74.71)	84.7 (78.6-90.9)	75.4 (58.4,79.4)
Nyctaginaceae	91.7 (84.8-95.7)	63.5 (48.74-70.83)	66.62 (64.05-68.91)	55.0 (37.3-71.4)	72 (63.1,77.9)
Molluginaceae	77.2 (89.3-97.2)	73.11 (54.22-84.99)	77.77 (75.62-80.99)	78.1 (63.6-93.4)	98.7 (94.3,104.7)
Montiaceae	67.3 (63.9-71.2)	42.8 (27.49-53.06)	60.87 (54.43-67.61)	58.5 (44.6-77.1)	53.6 (52.1,53.1)
Didiereaceae	62.5 (59.3-66.7)	36.78 (22.82-58.18)	58.12 (51.07-65.74)	40.2 (22.5-55.6)	50.6 (48.5,52.9)
Cactaceae	51.6 (48.7-55.2)	28.8 (15.08-48.15)	47.47 (40.82-54.99)	32.5 (21.4-42.5)	46.6 (44.4,48.1)

Table S11: Models tested for betalain pigmentation and drought tolerance (75% of specimens in arid areas)

Model	0 hidden states		1 hidden state		2 hidden state		3 hidden states	
	AICs	LogLikelihood	AICs	LogLikelihood	AICs	LogLikelihood	AICs	LogLikelihood
Fully dependent	2650.273	-1319.14	-	-				
Dependent Arid Zone forward and reverse	2657.536	-1323.77	2633.425	-1308.71				
Dependent Arid Zone forward and reverse betalains zero	-	-	2630.811	-1308.41				
Dependent Arid Zone forward	2666.702	-1329.35	2551.199	-1267.6				
Dependent Arid Zone forward belalains zero	-	-	2562.089	-1274.05				
Dependent Arid Zone reverse	2737.944	-1364.97	2514.039	-1249.02				
Dependent Arid Zone reverse belalains zero	-	-	2511.533	-1248.77				
Dependent except Arid Zone forward	2737.232	-1363.62	2515.276	-1249.64				
Dependent except Arid Zone forward betalains zero	-	-	2512.614	-1248.31				
Dependent except Arid Zone reverse	2661.893	-1325.95	2551.172	-1267.59				
Dependent except Arid Zone reverse betalains zero	-	-	2559.361	-1271.68				
Dependent betalain forward	2760.29	-1376.15	2493.365	-1238.68				
Dependent betalain forward betalains zero	-	-	2490.876	-1237.44				
Independent	2764.522	-1379.26	2492.589	-1238.3				
Independent betalain zero"	-	-	2490.096	-1238.05	2454.631	-1214.32	**2448.859**	**-1203.43**

*Rate estimates from best fitting model shown in Figure S24.

Table S12: Models tested for betalain pigmentation and drought tolerance (25% of specimens in arid areas)

Model	0 hidden states		1 hidden state		2 hidden state		3 hidden states	
	AICs	LogLikelihood	AICs	LogLikelihood	AICs	LogLikelihood	AICs	LogLikelihood
Fully dependent	3200.566	-1594.28	-	-				
Dependent Arid Zone forward and reverse	3201.911	-1595.96	3177.826	-1580.91				
Dependent Arid Zone forward and reverse betalains zero			3175.212	-1580.61				
Dependent Arid Zone forward	3201.527	-1596.76	2979.78	-1481.89				
Dependent Arid Zone forward belalains zero			2988.895	-1487.45				
Dependent Arid Zone reverse	3255.548	-1623.77	2994.926	-1489.46				
Dependent Arid Zone reverse belalains zero			2992.505	-1489.25				
Dependent except Arid Zone forward	3256.349	-1623.17	2997.585	-1490.79				
Dependent except Arid Zone forward betalains zero			2994.679	-1489.34				
Dependent except Arid Zone reverse	3200.622	-1595.31	2982.795	-1483.4				
Dependent except Arid Zone reverse betalains zero			2989.838	-1486.92				
Dependent betalain forward	3290.154	-1641.08	2929.278	-1456.64				
Dependent betalain forward betalains zero			2931.406	-1457.7				
Independent	3291.264	-1642.63	2935.731	-1459.87				
Independent betalain zero			2930.437	-1458.22	2920.416	-1447.21	**2913.047**	**-1435.52**

*Rate estimates from best fitting model shown in Figure S25.

Table S13: Models tested for betalain pigmentation and drought tolerance (75% of specimens in hot arid areas)

model	0 hidden states		1 hidden state		2 hidden states		3 hidden states	
	AICs	LogLikelihood	AICs	LogLikelihood	AICs	LogLikelihood	AICs	LogLikelihood
Fully dependent	1621.708	-804.854						
Dependent Arid Zone forward and reverse	1640.239	-815.12	1612.991	-798.496				
Dependent Arid Zone forward and reverse betalains zero	-	-	1610.378	-798.189				
Dependent Arid Zone forward	1638.415	-815.207	1614.117	-799.059				
Dependent Arid Zone forward belalains zero	-	-	1609.874	-797.937				
Dependent Arid Zone reverse	1685.56	-838.78	1568.87	-776.435				
Dependent Arid Zone reverse belalains zero	-	-	1566.253	-776.126				
Dependent except Arid Zone forward	1681.057	-835.529	1573.9	-778.95				
Dependent except Arid Zone forward betalains zero	-	-	1567.918	-775.959				
Dependent except Arid Zone reverse	1685.56	-838.78	1616.05	-800.025				
Dependent except Arid Zone reverse betalains zero	-	-	1604.171	-794.086				
Dependent betalain forward	1700.995	-846.497	1573.9	-778.95				
Dependent betalain forward betalains zero	-	-	1567.917	-775.959				
Independent	1707.221	-850.611	1568.87	-776.435				
Independent betalain zero	-	-	1566.253	-776.126	**1564.081**	**-769.041**	1565.439	-761.719

*Rate estimates from best fitting model shown in Figure S26.

Table S14: Models tested for betalain pigmentation and drought tolerance (25% of specimens in hot arid areas)

Model	0 hidden states		1 hidden states		2 hidden states		3 hidden states	
	AICs	LogLikelihood	AICs	LogLikelihood	AICs	LogLikelihood	AICs	LogLikelihood
Fully depepdent	2590.339	-1289.17	-	-	-	-	-	-
Independent	2712.095	-1353.05	2436.881	-1210.44	**2395.481**	**-1182.74**	-	-
Independent betalain zero	-	-	2425.946	-1205.97	2406.636	-1190.32	-	-
Depepdent Arid Zone forward and reverse	2595.464	-1292.73	2571.349	-1277.67	2580.487	-1277.24	-	-
Depepdent Arid Zone forward and reverse betalain zero	-	-	2568.729	-1277.36	-	-	-	-
Depepdent Arid Zone forward	2594.232	-1293.12	2491.075	-1237.54	2486.171	-1229.09	-	-
Depepdent Arid Zone forward betalain zero	-	-	2508.878	-1247.44	2498.618	-1237.31	-	-
Depepdent Arid Zone reverse	2655.318	-1323.66	2448.842	-1216.42	2438.523	-1205.26	-	-
Depepdent Arid Zone reverse betalain zero	-	-	2446.321	-1216.16	2434.022	-1205.01	-	-
Depepdent betalains forward	2708.564	-1350.28	2421.588	-1202.79	2407.353	-1189.68	-	-
Depepdent betalains forward betalain zero	-	-	2426.454	-1205.23	2407.577	-1189.79	-	-
Depepdent except Arid Zone forward	2655.33	-1322.67	2450.998	-1217.5	2438.208	-1206.1	-	-
Depepdent except Arid Zone forward betalain zero	-	-	2447.861	-1215.93	2435.968	-1204.98	-	-
Depepdent except Arid Zone reverse	2589.8	-1289.9	2492.783	-1238.39	2477.405	-1225.7	-	-
Depepdent except Arid Zone reverse betalain zero	-	-	2507.127	-1245.56	2498.053	-1236.03		

Table S15: Rate estimates for best fitting model for betalain pigmentation and drought tolerance (25% of specimens in hot arid areas). 0 = not dry + not betalain, 1 = not dry + betalain, 2 = dry + not betalain, 3 = dry + betalain)

		Hidden state 1				Hidden state 2				Hidden state 3			
	State	0	1	2	3	0	1	2	3	0	1	2	3
Hidden state 1	0	NA	0.002307	0.25	NA	0.01227	NA	NA	NA	0.014255	NA	NA	NA
	1	NA	NA	NA	0.25	NA	0.01227	NA	NA	NA	0.014255	NA	NA
	2	0.25	NA	NA	0.002307	NA	NA	0.01227	NA	NA	NA	0.014255	NA
	3	NA	0.25	NA	NA	NA	NA	NA	0.01227	NA	NA	NA	0.014255
Hidden state 2	0	0.030506	NA	NA	NA	NA	NA	0.026703	NA	0.002861	NA	NA	NA
	1	NA	0.030506	NA	NA	NA	NA	NA	0.026703	NA	0.002861	NA	NA
	2	NA	NA	0.030506	NA	0.25	NA	NA	NA	NA	NA	0.002861	NA
	3	NA	NA	NA	0.030506	NA	0.25	NA	NA	NA	NA	NA	0.002861
Hidden state 3	0	2.50E-05	NA	NA	NA	0.008063	NA	NA	NA	NA	NA	0.000284	NA
	1	NA	2.50E-05	NA	NA	NA	0.008063	NA	NA	NA	NA	NA	0.000284
	2	NA	NA	2.50E-05	NA	NA	NA	0.008063	NA	0.00486	NA	NA	NA
	3	NA	NA	NA	2.50E-05	NA	NA	NA	0.008063	NA	0.00486	NA	NA

Table S16: Models tested for betalain pigmentation and drought tolerance (75% of specimens in hot arid areas and tropical savannah)

Model	0 hidden states		1 hidden state		2 hidden states		3 hidden states	
	AICs	LogLikelihood	AICs	LogLikelihood	AICs	LogLikelihood	AICs	LogLikelihood
Fully dependent	2219.877	-1103.94	-	-	-	-		
Dependent Arid Zone forward and reverse	2230.356	-1110.18	2206.288	-1095.14	2215.689	-1094.84		
Dependent Arid Zone forward and reverse betalains zero	-	-	2203.674	-1094.84	-	-		
Dependent Arid Zone forward	2249.411	-1120.71	2168.853	-1076.43	2164.922	-1068.46		
Dependent Arid Zone forward belalains zero	-	-	2180.245	-1083.12	2186.639	-1081.32		
Dependent Arid Zone reverse	2320.621	-1156.31	2120.554	-1052.28	2124.749	-1048.37		
Dependent Arid Zone reverse belalains zero	-	-	2118.057	-1052.03	2108.503	-1042.25		
Dependent except Arid Zone forward	2319.183	-1154.59	2122.886	-1053.44	2124.813	-1049.41		
Dependent except Arid Zone forward betalains zero	-	-	2119.829	-1051.92	2118.511	-1046.26		
Dependent except Arid Zone reverse	2247.063	-1118.53	2167.487	-1075.74	2165.161	-1069.58		
Dependent except Arid Zone reverse betalains zero	-	-	2172.792	-1078.4	2178.859	-1076.43		
Dependent betalain forward	2347.362	-1169.68	2122.886	-1053.44	2110.886	-1041.44		
Dependent betalain forward betalains zero	-	-	2119.828	-1051.91	2105.217	-1038.61		
Independent	2349.463	-1171.73	2134.355	-1059.18	2103.269	-1036.63		
Independent betalain zero	-	-	2124.825	-1055.41	**2103.112**	**-1038.56**	2107.866	-1032.93

*Rate estimates from best fitting model shown in Figure S27.

Table S17: Models tested for betalain pigmentation and drought tolerance (25% of specimens in hot arid areas and tropical savannah)

model	0 hidden states		1 hidden state		2 hidden states		3 hidden states	
	AICs	LogLikelihood	AICs	LogLikelihood	AICs	LogLikelihood	AICs	LogLikelihood
Fully dependent	2945.234	-1466.62	-	-				
Dependent Arid Zone forward and reverse	2948.737	-1469.37	2924.642	-1454.32	2934.647	-1454.32		
Dependent Arid Zone forward and reverse betalains zero	-	-	2922.029	-1454.01	-	-		
Dependent Arid Zone forward	2952.714	-1472.36	2790.371	-1387.19	2769.997	-1371		
Dependent Arid Zone forward belalains zero	-	-	2818.722	-1402.36	2797.969	-1386.98		
Dependent Arid Zone reverse	3031.901	-1511.95	2729.213	-1356.61	2706.792	-1339.4		
Dependent Arid Zone reverse belalains zero	-	-	2726.788	-1356.39	2702.262	-1339.13		
Dependent except Arid Zone forward	3032.772	-1511.39	2730.583	-1357.29	2710.097	-1342.05		
Dependent except Arid Zone forward betalains zero	-	-	2728.472	-1356.24	2704.084	-1339.04		
Dependent except Arid Zone reverse	2950.476	-1470.24	2791.648	-1387.82	2772.923	-1373.46		
Dependent except Arid Zone reverse betalains zero	-	-	2817.849	-1400.92	2769.891	-1371.95		
Dependent betalain forward	3088.141	-1540.07	2698.571	-1341.29	2685.392	-1328.7		
Dependent betalain forward betalains zero	-	-	2700.411	-1342.21	2664.247	-1318.12		
Independent	3088.141	-1540.07	2708.54	-1346.27	2685.344	-1327.67		
Independent betalain zero	-	-	2699.542	-1342.77	**2662.702**	**-1318.35**		

Table S18: Rate estimates for best fitting model for betalain pigmentation and drought tolerance (25% of specimens in hot arid areas and tropical savannah). 0 = not dry + not betalain, 1 = not dry + betalain, 2 = dry + not betalain, 3 = dry + betalain.

		Hidden state 1				Hidden state 2				Hidden state 3			
	State	0	1	2	3	0	1	2	3	0	1	2	3
Hidden state 1	0	NA	0.011611	0.25	NA	0.007473	NA	NA	NA	0.009827	NA	NA	NA
	1	NA	NA	NA	0.25	NA	0.007473	NA	NA	NA	0.009827	NA	NA
	2	0.096134	NA	NA	0.011611	NA	NA	0.007473	NA	NA	NA	0.009827	NA
	3	NA	0.096134	NA	NA	NA	NA	NA	0.007473	NA	NA	NA	0.009827
Hidden state 2	0	2.50E-05	NA	NA	NA	NA	NA	0.170237	NA	0.01348	NA	NA	NA
	1	NA	2.50E-05	NA	NA	NA	NA	NA	0.170237	NA	0.01348	NA	NA
	2	NA	NA	2.50E-05	NA	0.25	NA	NA	NA	NA	NA	0.01348	NA
	3	NA	NA	NA	2.50E-05	NA	0.25	NA	NA	NA	NA	NA	0.01348
Hidden state 3	0	2.50E-05	NA	NA	NA	0.010158	NA	NA	NA	NA	NA	2.50E-05	NA
	1	NA	2.50E-05	NA	NA	NA	0.010158	NA	NA	NA	NA	NA	2.50E-05
	2	NA	NA	2.50E-05	NA	NA	NA	0.010158	NA	0.001335	NA	NA	NA
	3	NA	NA	NA	2.50E-05	NA	NA	NA	0.010158	NA	0.001335	NA	NA

Table S19: Models tested for succulence and drought tolerance (75% of specimens in arid areas).

names	0 hidden states		1 hidden states		2 Hidden states	
	AICs	LogLikelihood	AICs	LogLikelihood	AICs	LogLikelihood
Independent	4821.901	-2406.95	4356.071	-2168.04	4279.473	-2121.74
Dependent Arid Zone forward and reverse	4628.511	-2308.26	4506.132	-2243.07	4509.391	-2238.7
Dependent Arid Zone forward	4627.055	-2308.53	4419.024	-2199.51	4350.387	-2158.19
Dependent Arid Zone reverse	4741.382	-2365.69	4414.288	-2197.14	4323.31	-2144.66
Dependent Succulence forward and reverse	4802.806	-2395.4	4366.052	-2173.03	4344.797	-2156.4
Dependent Succulence forward	4805.16	-2397.58	4365.259	-2172.63	4305.331	-2135.67
Dependent Succulence reverse	4818.365	-2404.18	4354.962	-2167.48	**4275.272**	**-2120.64**
Dependent except Succulence forward	4629.826	-2307.91	4562.733	-2271.37	4541.363	-2255.68
Dependent except Succulence reverse	4622.051	-2304.03	4556.044	-2268.02	4494.214	-2232.11
Dependent except Arid Zone forward	4734.881	-2360.44	4429.181	-2204.59	4356.605	-2163.3
Dependent except Arid Zone reverse	4621.698	-2303.85	4453.71	-2216.85	4423.628	-2196.81
Dependent Arid Zone forward Succulence reverse	4628.241	-2308.12	4425.06	-2202.53	4405.844	-2186.92
Dependent Arid Zone reverse Succulence forward	4734.11	-2361.06	4427.375	-2203.69	4378.857	-2173.43
Dependent both forward	4620.425	-2304.21	4456.649	-2218.32	4405.478	-2186.74
Dependent both reverse	4742.073	-2365.04	4406.785	-2193.39	4334.224	-2151.11
Fully dependent	4623.427	-2303.71	-	-	-	-

*Rate estimates from best fitting model shown in Figure S32.

Table S20: Models tested for succulence and drought tolerance (25% of specimens in arid areas).

models	0 hidden states		1 hidden states		2 hidden stated	
	AICs	LogLikelihood	AICs	LogLikelihood	AICs	LogLikelihood
Independent	5365.864	-2678.93	4776.061	-2378.03	**4668.925**	**-2316.46**
Dependent Arid Zone forward and reverse	5192.141	-2590.07	5070.088	-2525.04	5073.893	-2520.95
Dependent Arid Zone forward	5197.263	-2593.63	4859.821	-2419.91	4781.071	-2373.54
Dependent Arid Zone reverse	5267.241	-2628.62	4829.742	-2404.87	4743.688	-2354.84
Dependent Succulence forward and reverse	5342.206	-2665.1	4757.017	-2368.51	4707.933	-2337.97
Dependent Succulence forward	5340.275	-2665.14	4756.851	-2368.43	4714.507	-2340.25
Dependent Succulence reverse	5367.795	-2678.9	4778.008	-2379	4679.042	-2322.52
Dependent except Succulence forward	5190.597	-2588.3	5135.507	-2557.75	5114.066	-2542.03
Dependent except Succulence reverse	5177.184	-2581.59	5095.598	-2537.8	5105.602	-2537.8
Dependent except Arid Zone forward	5197.263	-2593.63	4841.659	-2410.83	4813.825	-2391.91
Dependent except Arid Zone reverse	5267.241	-2628.62	4855.808	-2417.9	4825.701	-2397.85
Dependent Arid Zone forward Succulence reverse	5196.351	-2592.18	4858.315	-2419.16	4826.828	-2397.41
Dependent Arid Zone reverse Succulence forward	5255.287	-2621.64	4843.142	-2411.57	4794.094	-2381.05
Dependent both forward	5180.437	-2584.22	4856.878	-2418.44	4823.218	-2395.61
Dependent both reverse	5266.884	-2627.44	4828.683	-2404.34	4787.573	-2377.79
Fuly dependent	5175.764	-2579.88	-	-	-	-

Table S21: Rate estimates for best fitting model for succulent and drought tolerance (25% of specimens in arid areas). 0 = not dry + not succulent, 1 = not dry + succulent, 2 = dry + not succulent, 3 = dry + succulent.

		Hidden state 1				Hidden state 2				Hidden state 3			
	State	0	1	2	3	0	1	2	3	0	1	2	3
Hidden state 1	0	NA	0.000766	0.25	NA	0.023533	NA	NA	NA	0.005225	NA	NA	NA
	1	2.50E-05	NA	NA	0.25	NA	0.023533	NA	NA	NA	0.005225	NA	NA
	2	0.25	NA	NA	0.000766	NA	NA	0.023533	NA	NA	NA	0.005225	NA
	3	NA	0.25	2.50E-05	NA	NA	NA	NA	0.023533	NA	NA	NA	0.005225
Hidden state 2	0	0.017591	NA	NA	NA	NA	2.50E-05	2.50E-05	NA	2.50E-05	NA	NA	NA
	1	NA	0.017591	NA	NA	2.50E-05	NA	NA	2.50E-05	NA	2.50E-05	NA	NA
	2	NA	NA	0.017591	NA	0.003759	NA	NA	2.50E-05	NA	NA	2.50E-05	NA
	3	NA	NA	NA	0.017591	NA	0.003759	2.50E-05	NA	NA	NA	NA	2.50E-05
Hidden state 3	0	0.001996	NA	NA	NA	0.029153	NA	NA	NA	NA	0.046355	0.20393	NA
	1	NA	0.001996	NA	NA	NA	0.029153	NA	NA	0.038646	NA	NA	0.20393
	2	NA	NA	0.001996	NA	NA	NA	0.029153	NA	0.041778	NA	NA	0.046355
	3	NA	NA	NA	0.001996	NA	NA	NA	0.029153	NA	0.041778	0.038646	NA

Table S22: Models tested for succulence and drought tolerance (75% of specimens in hot arid areas).

names	0 hidden states		1 hidden state		2 hidden states	
	AICs	LogLikelihood	AICs	LogLikelihood	AICs	LogLikelihood
Independent	3567.732	-1779.87	3392.909	-1686.45	3251.397	-1607.7
Dependent Arid Zone forward and reverse	3470.008	-1729	3345.025	-1662.51	3322.87	-1645.44
Dependent Arid Zone forward	3477.475	-1733.74	3332.855	-1656.43	3285.898	-1625.95
Dependent Arid Zone reverse	3534.241	-1762.12	3355.489	-1667.74	3246.467	-1606.23
Dependent Succulence forward and reverse	3560.998	-1774.5	3373.685	-1676.84	3313.032	-1640.52
Dependent Succulence forward	3564.426	-1777.21	3350.809	-1665.4	3307.585	-1636.79
Dependent Succulence reverse	3564.251	-1777.13	3359.658	-1669.83	3288.212	-1627.11
Dependent except Succulence forward	3471.174	-1728.59	3401.065	-1690.53	3379.294	-1674.65
Dependent except Succulence reverse	3470.756	-1728.38	3388.519	-1684.26	3347.625	-1658.81
Dependent except Arid Zone forward	3531.488	-1758.74	3351.89	-1665.94	3311.128	-1640.56
Dependent except Arid Zone reverse	3478.579	-1732.29	3431.54	-1705.77	3392.629	-1681.31
Dependent Arid Zone forward Succulence reverse	3478.664	-1733.33	3383.413	-1681.71	3323.569	-1645.78
Dependent Arid Zone reverse Succulence forward	3532.576	-1760.29	3340.515	-1660.26	**3235.84**	**-1601.92**
Dependent both forward	3477.5	-1732.75	3366.368	-1673.18	3332.474	-1650.24
Dependent both reverse	3533.181	-1760.59	3354.222	-1667.11	3286.563	-1627.28

Table S23: Rate estimates for best fitting model for succulent and drought tolerance (75% of specimens in hot arid areas). 0 = not dry + not succulent, 1 = not dry + succulent, 2 = dry + not succulent, 3 = dry + succulent.

		Hidden state 1				Hidden state 2				Hidden state 3			
	State	0	1	2	3	0	1	2	3	0	1	2	3
Hidden state 1	0	NA	0.001213	0.25	NA	0.01348	NA	NA	NA	0.011014	NA	NA	NA
	1	0.00139	NA	NA	0.25	NA	0.01348	NA	NA	NA	0.011014	NA	NA
	2	0.25	NA	NA	0.007181	NA	NA	0.01348	NA	NA	NA	0.011014	NA
	3	NA	0.243783	0.00139	NA	NA	NA	NA	0.01348	NA	NA	NA	0.011014
Hidden state 2	0	0.005378	NA	NA	NA	NA	0.001213	0.028482	NA	2.50E-05	NA	NA	NA
	1	NA	0.005378	NA	NA	2.50E-05	NA	NA	0.028482	NA	2.50E-05	NA	NA
	2	NA	NA	0.005378	NA	0.25	NA	NA	0.007181	NA	NA	2.50E-05	NA
	3	NA	NA	NA	0.005378	NA	0.243783	2.50E-05	NA	NA	NA	NA	2.50E-05
Hidden state 3	0	0.000254	NA	NA	NA	4.84E-05	NA	NA	NA	NA	0.001213	0.004269	NA
	1	NA	0.000254	NA	NA	NA	4.84E-05	NA	NA	0.110814	NA	NA	0.004269
	2	NA	NA	0.000254	NA	NA	NA	4.84E-05	NA	0.25	NA	NA	0.007181
	3	NA	NA	NA	0.000254	NA	NA	NA	4.84E-05	NA	0.243783	0.110814	NA

Table S24: Models tested for succulence and drought tolerance (25% of specimens in hot arid areas)

names	1 rate		2 rates		3 rates	
	AICs	LogLikelihood	AICs	LogLikelihood	AICs	LogLikelihood
Independent	4826.687	-2409.34	4351.775	-2165.89	**4263.865**	**-2113.93**
Dependent Arid Zone forward and reverse	4658.665	-2323.33	4537.917	-2258.96	4549.918	-2258.96
Dependent Arid Zone forward	4673.966	-2331.98	4454.162	-2217.08	4355.69	-2160.85
Dependent Arid Zone reverse	4797.378	-2393.69	4433.079	-2206.54	4321.356	-2143.68
Dependent Succulence forward and reverse	4808.769	-2398.38	4359.715	-2169.86	4321.663	-2144.83
Dependent Succulence forward	4816.538	-2403.27	4367.188	-2173.59	4282.935	-2124.47
Dependent Succulence reverse	4792.76	-2390.38	4344.83	-2162.42	4308.806	-2137.4
Dependent except Succulence forward	4659.695	-2322.85	4591.913	-2285.96	4573.415	-2271.71
Dependent except Succulence reverse	4653.765	-2319.88	4571.122	-2275.56	4529.824	-2249.91
Dependent except Arid Zone forward	4787.732	-2386.87	4429.171	-2204.59	4376.718	-2173.36
Dependent except Arid Zone reverse	4671.623	-2328.81	4489.471	-2234.74	4449.185	-2209.59
Dependent Arid Zone forward Succulence reverse	4675.629	-2331.81	4469.239	-2224.62	4394.536	-2181.27
Dependent Arid Zone reverse Succulence forward	4792.289	-2390.14	4430.457	-2205.23	4339.634	-2153.82
Dependent both forward	4670.003	-2329	4480.272	-2230.14	4405.951	-2186.98
Dependent both reverse	4792.76	-2390.38	4425.523	-2202.76	4353.394	-2160.7
Dependent	4654.709	-2319.35	-	-	-	-

Table S25: Rate estimates for best fitting model for succulent and drought tolerance (25% of specimens in hot arid areas). 0 = not dry + not succulent, 1 = not dry + succulent, 2 = dry + not succulent, 3 = dry + succulent.

		Hidden state 1				Hidden state 2				Hidden state 3			
	State	0	1	2	3	0	1	2	3	0	1	2	3
Hidden state 1	0	NA	2.50E-05	0.211825	NA	0.027363	NA	NA	NA	2.50E-05	NA	NA	NA
	1	2.50E-05	NA	NA	0.211825	NA	0.027363	NA	NA	NA	2.50E-05	NA	NA
	2	0.25	NA	NA	2.50E-05	NA	NA	0.027363	NA	NA	NA	2.50E-05	NA
	3	NA	0.25	2.50E-05	NA	NA	NA	NA	0.027363	NA	NA	NA	2.50E-05
Hidden state 2	0	0.007093	NA	NA	NA	NA	2.50E-05	2.50E-05	NA	0.00348	NA	NA	NA
	1	NA	0.007093	NA	NA	2.50E-05	NA	NA	2.50E-05	NA	0.00348	NA	NA
	2	NA	NA	0.007093	NA	0.00723	NA	NA	2.50E-05	NA	NA	0.00348	NA
	3	NA	NA	NA	0.007093	NA	0.00723	2.50E-05	NA	NA	NA	NA	0.00348
Hidden state 3	0	0.024553	NA	NA	NA	0.010454	NA	NA	NA	NA	0.043995	0.048518	NA
	1	NA	0.024553	NA	NA	NA	0.010454	NA	NA	0.041338	NA	NA	0.048518
	2	NA	NA	0.024553	NA	NA	NA	0.010454	NA	0.084708	NA	NA	0.043995
	3	NA	NA	NA	0.024553	NA	NA	NA	0.010454	NA	0.084708	0.041338	NA

Table S26: Models tested for succulence and drought tolerance (75% of specimens in hot arid areas or tropical savannah)

	1 rate		2 rates		3 rates	
names	AICs	LogLikelihood	AICs	LogLikelihood	AICs	LogLikelihood
Independent	4410.175	-2201.09	4035.792	-2007.9	**3946.741**	**-1955.37**
Dependent Arid Zone forward and reverse	4279.659	-2133.83	4154.892	-2067.45	4155.276	-2061.64
Dependent Arid Zone forward	4307.43	-2148.72	4133.845	-2056.92	4078.943	-2022.47
Dependent Arid Zone reverse	4370.455	-2180.23	4055.521	-2017.76	3963.658	-1964.83
Dependent Succulence forward and reverse	4404.348	-2196.17	4067.273	-2023.64	4039.434	-2003.72
Dependent Succulence forward	4411.863	-2200.93	4036.956	-2008.48	3953.187	-1959.59
Dependent Succulence reverse	4402.593	-2196.3	4068.38	-2024.19	4005.182	-1985.59
Dependent except Succulence forward	4278.372	-2132.19	4203.134	-2091.57	4180.909	-2075.46
Dependent except Succulence reverse	4277.656	-2131.83	4196.528	-2088.26	4162.975	-2066.49
Dependent except Arid Zone forward	4367.04	-2176.52	4063.089	-2021.54	4044.518	-2007.26
Dependent except Arid Zone reverse	4307.923	-2146.96	4165.924	-2072.96	4146.341	-2058.17
Dependent Arid Zone forward Succulence reverse	4306.351	-2147.18	4165.359	-2072.68	4090.384	-2029.19
Dependent Arid Zone reverse Succulence forward	4372.376	-2180.19	4048.443	-2014.22	3973.399	-1970.7
Dependent both forward	4309.522	-2148.76	4144.917	-2062.46	4094.108	-2031.05
Dependent both reverse	4365.129	-2176.56	4064.102	-2022.05	4011.258	-1989.63
Dependent	4276.502	-2130.25	-	-	-	-

Table S27: Rate estimates for best fitting model for succulent and drought tolerance (75% of specimens in hot arid areas or tropical savannah). 0 = not dry + not succulent, 1 = not dry + succulent, 2 = dry + not succulent, 3 = dry + succulent.

		Hidden state 1				Hidden state 2				Hidden state 3			
	State	0	1	2	3	0	1	2	3	0	1	2	3
Hidden state 1	0	NA	2.50E-05	0.181143	NA	0.006923	NA	NA	NA	0.000464	NA	NA	NA
	1	2.50E-05	NA	NA	0.181143	NA	0.006923	NA	NA	NA	0.000464	NA	NA
	2	0.239303	NA	NA	2.50E-05	NA	NA	0.006923	NA	NA	NA	0.000464	NA
	3	NA	0.239303	2.50E-05	NA	NA	NA	NA	0.006923	NA	NA	NA	0.000464
Hidden state 2	0	8.02E-05	NA	NA	NA	NA	2.50E-05	0.010367	NA	0.00321	NA	NA	NA
	1	NA	8.02E-05	NA	NA	2.50E-05	NA	NA	0.010367	NA	0.00321	NA	NA
	2	NA	NA	8.02E-05	NA	0.25	NA	NA	2.50E-05	NA	NA	0.00321	NA
	3	NA	NA	NA	8.02E-05	NA	0.25	2.50E-05	NA	NA	NA	NA	0.00321
Hidden state 3	0	0.014659	NA	NA	NA	0.011907	NA	NA	NA	NA	0.019717	0.005446	NA
	1	NA	0.014659	NA	NA	NA	0.011907	NA	NA	0.02946	NA	NA	0.005446
	2	NA	NA	0.014659	NA	NA	NA	0.011907	NA	0.008236	NA	NA	0.019717
	3	NA	NA	NA	0.014659	NA	NA	NA	0.011907	NA	0.008236	0.02946	NA

Table S28: Models tested for succulence and drought tolerance (25% of specimens in hot arid areas or tropical savannah).

names	1 rate		2 rates		3 rates	
	AICs	LogLikelihood	AICs	LogLikelihood	AICs	LogLikelihood
Fully dependent	5067.765	-2525.88	-	-	-	-
Independent	5255.708	-2623.85	4644.594	-2312.3	**4553.544**	**-2258.77**
Dependent Arid Zone forward and reverse	5072.36	-2530.18	4953.013	-2466.51	4947.596	-2457.8
Dependent Arid Zone forward	5143.191	-2566.6	4817.249	-2398.62	4705.656	-2335.83
Dependent Arid Zone reverse	5256.836	-2623.42	4706.869	-2343.43	4609.723	-2287.86
Dependent Succulence forward and reverse	5232.945	-2610.47	4652.983	-2316.49	4621.655	-2294.83
Dependent Succulence forward	5256.348	-2623.17	4663.022	-2321.51	4610.573	-2288.29
Dependent Succulence reverse	5231.704	-2610.85	4635.102	-2307.55	4583.159	-2274.58
Dependent except Succulence forward	5066.001	-2526	4994.022	-2487.01	4973.486	-2471.74
Dependent except Succulence reverse	5074.357	-2530.18	4994.682	-2487.34	4936.886	-2453.44
Dependent except Arid Zone forward	5232.206	-2609.1	4726.082	-2353.04	4658.298	-2314.15
Dependent except Arid Zone reverse	5140.838	-2563.42	4817.484	-2398.74	4768.617	-2369.31
Dependent Arid Zone forward Succulence reverse	5138.812	-2563.41	4818.328	-2399.16	4722.066	-2345.03
Dependent Arid Zone reverse Succulence forward	5257.337	-2622.67	4702.995	-2341.5	4607.035	-2287.52
Dependent both forward	5145.196	-2566.6	4818.807	-2399.4	4765.132	-2366.57
Dependent both reverse	5231.122	-2609.56	4730.141	-2355.07	4626.246	-2297.12

Table S29: Rate estimates for best fitting model for succulent and drought tolerance (25% of specimens in hot arid areas or tropical savannah). 0 = not dry + not succulent, 1 = not dry + succulent, 2 = dry + not succulent, 3 = dry + succulent.

		Hidden state 1				Hidden state 2				Hidden state 3			
	State	0	1	2	3	0	1	2	3	0	1	2	3
Hidden state 1	0	NA	2.50E-05	0.25	NA	0.019428	NA	NA	NA	2.50E-05	NA	NA	NA
	1	2.50E-05	NA	NA	0.25	NA	0.019428	NA	NA	NA	2.50E-05	NA	NA
	2	0.223479	NA	NA	2.50E-05	NA	NA	0.019428	NA	NA	NA	2.50E-05	NA
	3	NA	0.223479	2.50E-05	NA	NA	NA	NA	0.019428	NA	NA	NA	2.50E-05
Hidden state 2	0	0.007316	NA	NA	NA	NA	2.50E-05	2.50E-05	NA	0.003752	NA	NA	NA
	1	NA	0.007316	NA	NA	0.000387	NA	NA	2.50E-05	NA	0.003752	NA	NA
	2	NA	NA	0.007316	NA	0.001868	NA	NA	2.50E-05	NA	NA	0.003752	NA
	3	NA	NA	NA	0.007316	NA	0.001868	0.000387	NA	NA	NA	NA	0.003752
Hidden state 3	0	0.018565	NA	NA	NA	0.019016	NA	NA	NA	NA	0.022491	0.043497	NA
	1	NA	0.018565	NA	NA	NA	0.019016	NA	NA	0.039945	NA	NA	0.043497
	2	NA	NA	0.018565	NA	NA	NA	0.019016	NA	0.0888	NA	NA	0.022491
	3	NA	NA	NA	0.018565	NA	NA	NA	0.019016	NA	0.0888	0.039945	NA

Table S30: Models tested for medullary bundles and drought tolerance (75% of specimens in arid areas).

Model	0 hidden states		1 hidden states		2 hidden states	
	AICs	LogLikelihood	AICs	LogLikelihood	AICs	LogLikelihood
Independent	802.5777	-397.289	872.1832	-426.092	**738.5405**	**-351.27**
Dependent Arid Zone forward and reverse	802.636	-395.318	899.9738	-439.987	-	-
Dependent Arid Zone forward	800.6964	-395.348	902.2311	-441.116	-	-
Dependent Arid Zone reverse	803.7184	-396.859	880.1097	-430.055	-	-
Dependent Medullary Bundles forward and reverse	804.8499	-396.425	903.8605	-441.93	-	-
Dependent Medullary Bundles forward	802.8532	-396.427	906.2786	-443.139	-	-
Dependent Medullary Bundles reverse	804.552	-397.276	903.5415	-441.771	-	-
Dependent except Medullary Bundles forward	801.7217	-393.861	900.4835	-440.242	-	-
Dependent except Medullary Bundles reverse	803.0527	-394.526	927.9843	-453.992	-	-
Dependent except Arid Zone forward	806.3845	-396.192	910.1721	-445.086	-	-
Dependent except Arid Zone reverse	803.1698	-394.585	929.67	-454.835	-	-
Dependent Arid Zone forward Medullary Bundles reverse	800.7275	-394.364	920.091	-450.045	-	-
Dependent Arid Zone reverse Medullary Bundles forward	804.3843	-396.192	916.5252	-448.263	-	-
Dependent both forward	801.3461	-394.673	910.5925	-445.296	-	-
Dependent both reverse	805.6889	-396.844	899.4296	-439.715	-	-
Full dependent	804.5553	-394.278	-	-	-	-

*Rate estimates from best fitting model shown in Figure S35.

Table S31: Models tested for medullary bundles and drought tolerance (25% of specimens in arid areas).

model	0 hidden states		1 hidden states		2 hidden states	
	AICs	LogLikelihood	AICs	LogLikelihood	AICs	LogLikelihood
Fully dependent	953.0101	-468.505	-	-	-	-
Dependent Arid Zone forward and reverse	950.8331	-469.417	899.9738	-439.987	-	-
Dependent Medullary Bundles forward and reverse	962.8257	-475.413	903.8605	-441.93	-	-
Dependent both forward	961.0682	-474.534	910.5925	-445.296	-	-
Dependent both reverse	965.4436	-476.722	899.4296	-439.715	-	-
Dependent Arid Zone forward	956.4293	-473.215	902.2311	-441.116	-	-
Dependent Medullary Bundles forward	963.0063	-476.503	906.2786	-443.139	-	-
Dependent Arid Zone reverse	963.1347	-476.567	880.1097	-430.055	-	-
Dependent Medullary Bundles reverse	961.4089	-475.704	903.5415	-441.771	-	-
Dependent except Arid Zone forward	963.5279	-474.764	910.1721	-445.086	-	-
Dependent except Arid Zone reverse	958.893	-472.447	929.67	-454.835	-	-
Dependent except Medullary Bundles forward	951.6989	-468.849	900.4835	-440.242	-	-
Dependent except Medullary Bundles reverse	952.1608	-469.08	927.9843	-453.992	-	-
Dependent Arid Zone in Medullary Bundles reverse	956.975	-472.487	920.091	-450.045	-	-
Dependent Arid Zone out Medullary Bundles forward	963.4179	-475.709	916.5252	-448.263	-	-
independent	961.9656	-476.983	872.1832	-426.092	**856.4238**	**-410.212**

Table S32: Rate estimates for best fitting model for medullary bundles and drought tolerance (25% of specimens in arid areas). 0 = not dry + not medullary bundles, 1 = not dry + medullary bundles, 2 = dry + not medullary bundles, 3 = dry + medullary bundles.

		Hidden state 1				Hidden state 2				Hidden state 3			
	State	0	1	2	3	0	1	2	3	0	1	2	3
Hidden state 1	0	NA	0.003193	0.223906	NA	0.024827	NA	NA	NA	0.001216	NA	NA	NA
	1	0.01186	NA	NA	0.223906	NA	0.024827	NA	NA	NA	0.001216	NA	NA
	2	0.25	NA	NA	0.003193	NA	NA	0.024827	NA	NA	NA	0.001216	NA
	3	NA	0.25	0.01186	NA	NA	NA	NA	0.024827	NA	NA	NA	0.001216
Hidden state 2	0	0.008261	NA	NA	NA	NA	0.002435	2.50E-05	NA	2.50E-05	NA	NA	NA
	1	NA	0.008261	NA	NA	0.144567	NA	NA	2.50E-05	NA	2.50E-05	NA	NA
	2	NA	NA	0.008261	NA	0.000437	NA	NA	0.002435	NA	NA	2.50E-05	NA
	3	NA	NA	NA	0.008261	NA	0.000437	0.144567	NA	NA	NA	NA	2.50E-05
Hidden state 3	0	2.50E-05	NA	NA	NA	2.50E-05	NA	NA	NA	NA	2.50E-05	0.005524	NA
	1	NA	2.50E-05	NA	NA	NA	2.50E-05	NA	NA	2.50E-05	NA	NA	0.005524
	2	NA	NA	2.50E-05	NA	NA	NA	2.50E-05	NA	0.01263	NA	NA	2.50E-05
	3	NA	NA	NA	2.50E-05	NA	NA	NA	2.50E-05	NA	0.01263	2.50E-05	NA

Table S33: Models tested for medullary bundles and drought tolerance (75% of specimens in hot arid areas).

Model	0 hidden states		1 hidden states		2 hidden states	
	AICs	LogLikelihood	AICs	LogLikelihood	AICs	LogLikelihood
Independent	640.1339	-316.067	592.5086	-286.254	589.6486	-276.824
Dependent Arid Zone forward and reverse	637.4164	-312.708	587.7631	-283.882	-	-
Dependent Arid Zone forward	641.8538	-315.927	592.2001	-286.1	-	-
Dependent Arid Zone reverse	638.3068	-314.153	**585.2576**	**-282.629**	-	-
Dependent Medullary Bundles forward and reverse	628.5167	-308.258	633.7726	-306.886	-	-
Dependent Medullary Bundles forward	639.1966	-314.598	598.8629	-289.431	-	-
Dependent Medullary Bundles reverse	627.6752	-308.838	592.9695	-286.485	-	-
Dependent except Medullary Bundles forward	630.534	-308.267	590.3108	-285.155	-	-
Dependent except Medullary Bundles reverse	631.8996	-308.95	613.949	-296.975	-	-
Dependent except Arid Zone forward	629.9789	-307.989	634.0196	-307.01	-	-
Dependent except Arid Zone reverse	630.4867	-308.243	634.3986	-307.199	-	-
Dependent Arid Zone forward Medullary Bundles reverse	629.367	-308.684	594.4513	-287.226	-	-
Dependent Arid Zone reverse Medullary Bundles forward	633.1337	-310.567	594.4841	-287.242	-	-
Dependent both forward	640.9305	-314.465	598.5629	-289.281	-	-
Dependent both reverse	629.3296	-308.665	587.1836	-283.592	-	-
Full dependent	630.5988	-307.299	-	-	-	-

Table S34: Rate estimates for best fitting model for medullary bundles and drought tolerance (75% of specimens in hot arid areas). 0 = not dry + not medullary bundles, 1 = not dry + medullary bundles, 2 = dry + not medullary bundles, 3 = dry + medullary bundles.

		Hidden state 1				Hidden state 2			
	State	0	1	2	3	0	1	2	3
Hidden state 1	0	NA	0.000271	0.0097	NA	0.00265	NA	NA	NA
	1	2.50E-05	NA	NA	0.0097	NA	0.00265	NA	NA
	2	0.102948	NA	NA	0.000271	NA	NA	0.00265	NA
	3	NA	0.205449	2.50E-05	NA	NA	NA	NA	0.00265
Hidden state 2	0	0.064305	NA	NA	NA	NA	0.106449	0.031289	NA
	1	NA	0.064305	NA	NA	0.146929	NA	NA	0.031289
	2	NA	NA	0.064305	NA	0.102948	NA	NA	0.106449
	3	NA	NA	NA	0.064305	NA	0.205449	0.146929	NA

Table S35: Models tested for medullary bundles and drought tolerance (25% of specimens in hot arid areas).

names	1 rate		2 rates		3 rates	
	AICs	LogLikelihood	AICs	LogLikelihood	AICs	LogLikelihood
Independent	901.0621	-446.531	850.6938	-415.347	**815.1322**	**-389.566**
Dependent Arid Zone forward and reverse	891.6775	-439.839	841.6976	-410.849	840.9	-404.45
Dependent Arid Zone forward	896.2343	-443.117	846.5732	-413.287	835.4844	-400.742
Dependent Arid Zone reverse	903.002	-446.501	831.2732	-405.637	849.3246	-407.662
Dependent Medullary Bundles forward and reverse	899.2746	-443.637	862.8796	-421.44	869.9069	-418.953
Dependent Medullary Bundles forward	901.1802	-445.59	854.9672	-417.484	830.1608	-398.08
Dependent Medullary Bundles reverse	898.4136	-444.207	861.1491	-420.575	845.4447	-405.722
Dependent except Medullary Bundles forward	893.6289	-439.814	844.2296	-412.115	849.8277	-409.914
Dependent except Medullary Bundles reverse	890.6735	-438.337	852.0661	-416.033	852.2963	-411.148
Dependent except Arid Zone forward	901.0601	-443.53	871.0939	-425.547	879.6272	-424.814
Dependent except Arid Zone reverse	899.3362	-442.668	876.6799	-428.34	873.9743	-421.987
Dependent Arid Zone forward Medullary Bundles reverse	898.2232	-443.112	874.5011	-427.251	834.6847	-401.342
Dependent Arid Zone reverse Medullary Bundles forward	901.7981	-444.899	843.7805	-411.89	842.66	-405.33
Dependent both forward	897.0383	-442.519	851.9134	-415.957	844.9353	-406.468
Dependent both reverse	899.8022	-443.901	875.4634	-427.732	835.3899	-401.695
Full dependent	892.627	-438.314	-	-	-	-

Table S36: Rate estimates for best fitting model for medullary bundles and drought tolerance (25% specimens in hot arid areas). 0 = not dry + not medullary bundles, 1 = not dry + medullary bundles, 2 = dry + not medullary bundles, 3 = dry + medullary bundles.

		Hidden state 1				Hidden state 2				Hidden state 3			
	State	0	1	2	3	0	1	2	3	0	1	2	3
Hidden state 1	0	NA	0.002111	0.139259	NA	0.03886	NA	NA	NA	0.008623	NA	NA	NA
	1	0.009036	NA	NA	0.139259	NA	0.03886	NA	NA	NA	0.008623	NA	NA
	2	0.25	NA	NA	0.002111	NA	NA	0.03886	NA	NA	NA	0.008623	NA
	3	NA	0.25	0.009036	NA	NA	NA	NA	0.03886	NA	NA	NA	0.008623
Hidden state 2	0	0.012449	NA	NA	NA	NA	0.000251	2.50E-05	NA	2.50E-05	NA	NA	NA
	1	NA	0.012449	NA	NA	2.50E-05	NA	NA	2.50E-05	NA	2.50E-05	NA	NA
	2	NA	NA	0.012449	NA	0.00059	NA	NA	0.000251	NA	NA	2.50E-05	NA
	3	NA	NA	NA	0.012449	NA	0.00059	2.50E-05	NA	NA	NA	NA	2.50E-05
Hidden state 3	0	2.50E-05	NA	NA	NA	2.50E-05	NA	NA	NA	NA	0.068904	2.50E-05	NA
	1	NA	2.50E-05	NA	NA	NA	2.50E-05	NA	NA	0.25	NA	NA	2.50E-05
	2	NA	NA	2.50E-05	NA	NA	NA	2.50E-05	NA	0.033883	NA	NA	0.068904
	3	NA	NA	NA	2.50E-05	NA	NA	NA	2.50E-05	NA	0.033883	0.25	NA

Table S37: Models tested for medullary bundles and drought tolerance (75% of specimens in hot arid areas or tropical savannah).

Model	0 hidden states		1 hidden states		2 hidden states	
	AICs	LogLikelihood	AICs	LogLikelihood	AICs	LogLikelihood
Full dependent	837.9539	-410.977	-	-	-	-
Independent	846.0823	-419.041	795.0572	-387.529	781.0967	-372.548
Dependent Arid Zone forward and reverse	840.9115	-414.456	790.8565	-385.428	796.0977	-382.049
Dependent Arid Zone forward	846.0128	-418.006	791.6468	-385.823	790.1563	-378.078
Dependent Arid Zone reverse	847.2469	-418.623	795.0371	-387.519	**772.104**	**-369.052**
Dependent Medullary Bundles forward and reverse	838.3189	-413.159	817.9507	-398.975	825.7716	-396.886
Dependent Medullary Bundles forward	846.5338	-418.267	802.3795	-391.19	786.2758	-376.138
Dependent Medullary Bundles reverse	836.8854	-413.443	790.9308	-385.465	785.4748	-375.737
Dependent except Medullary Bundles forward	837.2971	-411.649	790.4937	-385.247	795.9352	-382.968
Dependent except Medullary Bundles reverse	838.7811	-412.391	798.7061	-389.353	800.2112	-385.106
Dependent except Arid Zone forward	840.3095	-413.155	820.2311	-400.116	828.3792	-399.19
Dependent except Arid Zone reverse	838.3091	-412.155	832.5799	-406.29	834.3928	-402.196
Dependent Arid Zone forward Medullary Bundles reverse	836.5792	-412.29	799.5711	-389.786	836.5102	-402.255
Dependent Arid Zone reverse Medullary Bundles forward	846.3809	-417.191	801.5327	-390.766	784.8459	-376.423
Dependent both forward	846.6943	-417.347	797.5203	-388.76	799.9789	-383.989
Dependent both reverse	838.8432	-413.422	820.0181	-400.009	783.1349	-375.568

Table S38: Rate estimates for best fitting model for medullary bundles and drought tolerance (75% of specimens in hot arid areas or tropical savannah). 0 = not dry + not medullary bundles, 1 = not dry + medullary bundles, 2 = dry + not medullary bundles, 3 = dry + medullary bundles.

		Hidden state 1				Hidden state 2				Hidden state 3			
	State	0	1	2	3	0	1	2	3	0	1	2	3
Hidden state 1	0	NA	0.055973	0.137906	NA	2.50E-05	NA	NA	NA	0.007108	NA	NA	NA
	1	2.50E-05	NA	NA	0.137906	NA	2.50E-05	NA	NA	NA	0.007108	NA	NA
	2	0.106025	NA	NA	0.055973	NA	NA	2.50E-05	NA	NA	NA	0.007108	NA
	3	NA	0.236821	2.50E-05	NA	NA	NA	NA	2.50E-05	NA	NA	NA	0.007108
Hidden state 2	0	0.000891	NA	NA	NA	NA	2.50E-05	0.055658	NA	2.50E-05	NA	NA	NA
	1	NA	0.000891	NA	NA	2.50E-05	NA	NA	0.055658	NA	2.50E-05	NA	NA
	2	NA	NA	0.000891	NA	0.106025	NA	NA	2.50E-05	NA	NA	2.50E-05	NA
	3	NA	NA	NA	0.000891	NA	0.236821	2.50E-05	NA	NA	NA	NA	2.50E-05
Hidden state 3	0	2.50E-05	NA	NA	NA	0.005844	NA	NA	NA	NA	0.012416	0.005819	NA
	1	NA	2.50E-05	NA	NA	NA	0.005844	NA	NA	0.167699	NA	NA	0.005819
	2	NA	NA	2.50E-05	NA	NA	NA	0.005844	NA	0.106025	NA	NA	0.012416
	3	NA	NA	NA	2.50E-05	NA	NA	NA	0.005844	NA	0.236821	0.167699	NA

Table S39: Models tested for medullary bundles and drought tolerance (25% of specimens in hot arid areas or tropical savannah).

names	1 rate		2 rates		3 rates	
	AICs	LogLikelihood	AICs	LogLikelihood	AICs	LogLikelihood
Dependent	912.7607	-448.38	-	-	-	-
Independent	923.3688	-457.684	850.4718	-415.236	**826.5097**	**-395.255**
Dependent Arid Zone forward and reverse	912.6278	-450.314	862.1966	-421.098	-	-
Dependent Arid Zone forward	921.1382	-455.569	878.1643	-429.082	-	-
Dependent Arid Zone reverse	923.372	-456.686	857.5556	-418.778	-	-
Dependent Medullary Bundles forward and reverse	926.6799	-457.34	874.3894	-427.195	-	-
Dependent Medullary Bundles forward	924.6892	-457.345	863.9063	-421.953	-	-
Dependent Medullary Bundles reverse	925.3682	-457.684	851.2196	-415.61	-	-
Dependent except Medullary Bundles forward	912.3845	-449.192	861.4984	-420.749	-	-
Dependent except Medullary Bundles reverse	913.0556	-449.528	869.9639	-424.982	-	-
Dependent except Arid Zone forward	925.6982	-455.849	888.8837	-434.442	-	-
Dependent except Arid Zone reverse	923.1423	-454.571	892.4041	-436.202	-	-
Dependent Arid Zone forward Medullary Bundles reverse	921.4645	-454.732	889.4615	-434.731	-	-
Dependent Arid Zone reverse Medullary Bundles forward	923.7031	-455.852	870.8755	-425.438	-	-
Dependent both forward	922.9028	-455.451	878.6231	-429.312	-	-
Dependent both reverse	925.3279	-456.664	884.7492	-432.375	-	-

Table S40: Rate estimates for best fitting model for medullary bundles and drought tolerance (25% of specimens in hot arid areas or tropical savannah). 0 = not dry + not medullary bundles, 1 = not dry + medullary bundles, 2 = dry + not medullary bundles, 3 = dry + medullary bundles.

		Hidden state 1				Hidden state 2				Hidden state 3			
	State	0	1	2	3	0	1	2	3	0	1	2	3
Hidden state 1	0	NA	2.50E-05	0.230905	NA	0.045102	NA	NA	NA	0.003292	NA	NA	NA
	1	2.50E-05	NA	NA	0.230905	NA	0.045102	NA	NA	NA	0.003292	NA	NA
	2	0.25	NA	NA	2.50E-05	NA	NA	0.045102	NA	NA	NA	0.003292	NA
	3	NA	0.25	2.50E-05	NA	NA	NA	NA	0.045102	NA	NA	NA	0.003292
Hidden state 2	0	0.008826	NA	NA	NA	NA	0.000582	2.50E-05	NA	0.002284	NA	NA	NA
	1	NA	0.008826	NA	NA	2.50E-05	NA	NA	2.50E-05	NA	0.002284	NA	NA
	2	NA	NA	0.008826	NA	2.50E-05	NA	NA	0.000582	NA	NA	0.002284	NA
	3	NA	NA	NA	0.008826	NA	2.50E-05	2.50E-05	NA	NA	NA	NA	0.002284
Hidden state 3	0	0.060353	NA	NA	NA	2.50E-05	NA	NA	NA	NA	0.082859	2.50E-05	NA
	1	NA	0.060353	NA	NA	NA	2.50E-05	NA	NA	0.147096	NA	NA	2.50E-05
	2	NA	NA	0.060353	NA	NA	NA	2.50E-05	NA	0.013393	NA	NA	0.082859
	3	NA	NA	NA	0.060353	NA	NA	NA	2.50E-05	NA	0.013393	0.147096	NA

Table S41: Models tested for succulence and betalains.

Model	0 hidden states		1 hidden state		2 hidden states	
	AICs	LogLikelihood	AICs	LogLikelihood	AICs	LogLikelihood
Dependent	614.9005	-301.45	-	-	-	-
Dependent both forward	624.8732	-307.437	565.5603	-274.78	528.5464	-251.273
Dependent both forward zero	-	-	562.6186	-273.309	550.1112	-262.056
Dependent succulence forward	623.0777	-307.539	565.0923	-274.546	527.573	-249.787
Dependent succulence forward zero	-	-	562.6268	-274.313	558.7598	-267.38
Dependent betalain forward	626.2076	-309.104	526.8096	-255.405	513.6601	-242.83
Dependent betalain forward zero	-	-	546.4875	-265.244	515.1014	-243.551
Dependent succulence	612.9266	-301.463	588.9833	-286.492	598.143	-286.072
Dependent succulence zero	-	-	586.378	-286.189	-	-
Dependent succulence reverse	613.1192	-302.56	567.8384	-275.919	563.0531	-267.527
Dependent succulence reverse zero	-	-	578.2374	-282.119	551.9747	-263.987
Dependent except succulence forward	614.753	-302.377	579.7417	-281.871	577.3563	-275.678
Dependent except succulence forward zero	-	-	571.6777	-277.839	568.5335	-271.267
Independent	626.2076	-309.104	524.4482	-254.224	**508.5001**	**-239.25**
Independent zero	-	-	552.5438	-269.272	517.3941	-245.697

*Rate estimates from best fitting model shown in Figure S36.

Table S42: Models tested for medullary bundles and betalains.

Model	0 hidden states		1 hidden state		2 hidden states	
	AICs	LogLikelihood	AICs	LogLikelihood	AICs	LogLikelihood
Dependent	229.1629	-108.582	-	-	-	-
Dependent zero	-	-	221.6059	-102.803	-	-
Dependent both forward	242.7275	-116.364	216.8045	-100.402	220.3572	-97.1786
Dependent both forward zero	-	-	216.4281	-100.214	216.9866	-95.4933
Dependent Medullary Bundles forward	253.1002	-122.55	225.2224	-104.611	221.6952	-96.8476
Dependent Medullary Bundles forward zero	-	-	229.2388	-107.619	215.1488	-95.5744
Dependent betalain forward	241.0199	-116.51	215.0939	-99.547	220.8872	-96.4436
Dependent betalain forward zero	-	-	209.582	-96.791	215.7339	-93.867
Dependent Medullary Bundles	234.1077	-112.054	221.6517	-102.826	230.3573	-102.179
Dependent Medullary Bundles zero	-	-	219.4944	-102.747	-	-
Dependent Medullary Bundles reverse	237.0244	-114.512	221.776	-102.888	204.0966	-88.0483
Dependent Medullary Bundles reverse zero	-	-	206.7943	-96.3971	**199.9393**	**-87.9696**
Dependent except Medullary Bundles forward	242.8253	-114.413	210.7724	-97.3862	219.1991	-96.5995
Dependent except Medullary Bundles forward zero	-	-	210.9865	-97.4932	201.1428	-87.5714
Independent	250.9523	-122.476	223.5166	-103.758	228.4865	-99.2432
Independent zero	-	-	217.4576	-101.729	215.3045	-94.6523

*Rate estimates from best fitting model shown in Figure S37.

Table S43: Models tested for medullary bundles and succulence.

names	0 hidden states		1 hidden state		2 hidden states	
	AICs	LogLikelihood	AICs	LogLikelihood	AICs	LogLikelihood
Independent	501.076	-246.538	440.9798	-210.49	**419.8301**	**-191.915**
Dependent Medullary Bundles forward and reverse	478.9869	-233.493	458.5259	-219.263	470.5259	-219.263
Dependent Medullary Bundles forward	503.0252	-246.513	460.2621	-220.131	441.2806	-203.64
Dependent Medullary Bundles reverse	477.4481	-233.724	440.8843	-210.442	431.6287	-198.814
Dependent Succulence forward and reverse	498.3229	-243.161	429.6175	-204.809	434.7717	-201.386
Dependent Succulence forward	498.5157	-244.258	434.0422	-207.021	436.0853	-201.043
Dependent Succulence reverse	477.131	-231.566	437.9587	-208.979	431.6963	-198.848
Dependent except succulence forward	478.0587	-232.029	464.4591	-222.23	466.5821	-218.291
Dependent except succulence reverse	477.131	-231.566	458.02	-219.01	466.4269	-218.213
Dependent except Medullary Bundles forward	503.0252	-246.513	444.3591	-212.18	454.3669	-212.183
Dependent except Medullary Bundles reverse	477.4481	-233.724	449.6996	-214.85	447.3801	-208.69
Dependent Medullary Bundles forward Succulence reverse	502.9282	-245.464	461.5594	-220.78	439.2806	-203.64
Dependent Medullary Bundles reverse Succulence forward	475.5316	-231.766	441.7666	-210.883	434.4822	-201.241
Dependent both forward	500.4393	-244.22	441.9632	-210.982	435.7748	-201.887
Dependent both reverse	476.5096	-232.255	465.6953	-222.848	437.9043	-202.952

*Rate estimates from best fitting model shown in Figure S38.

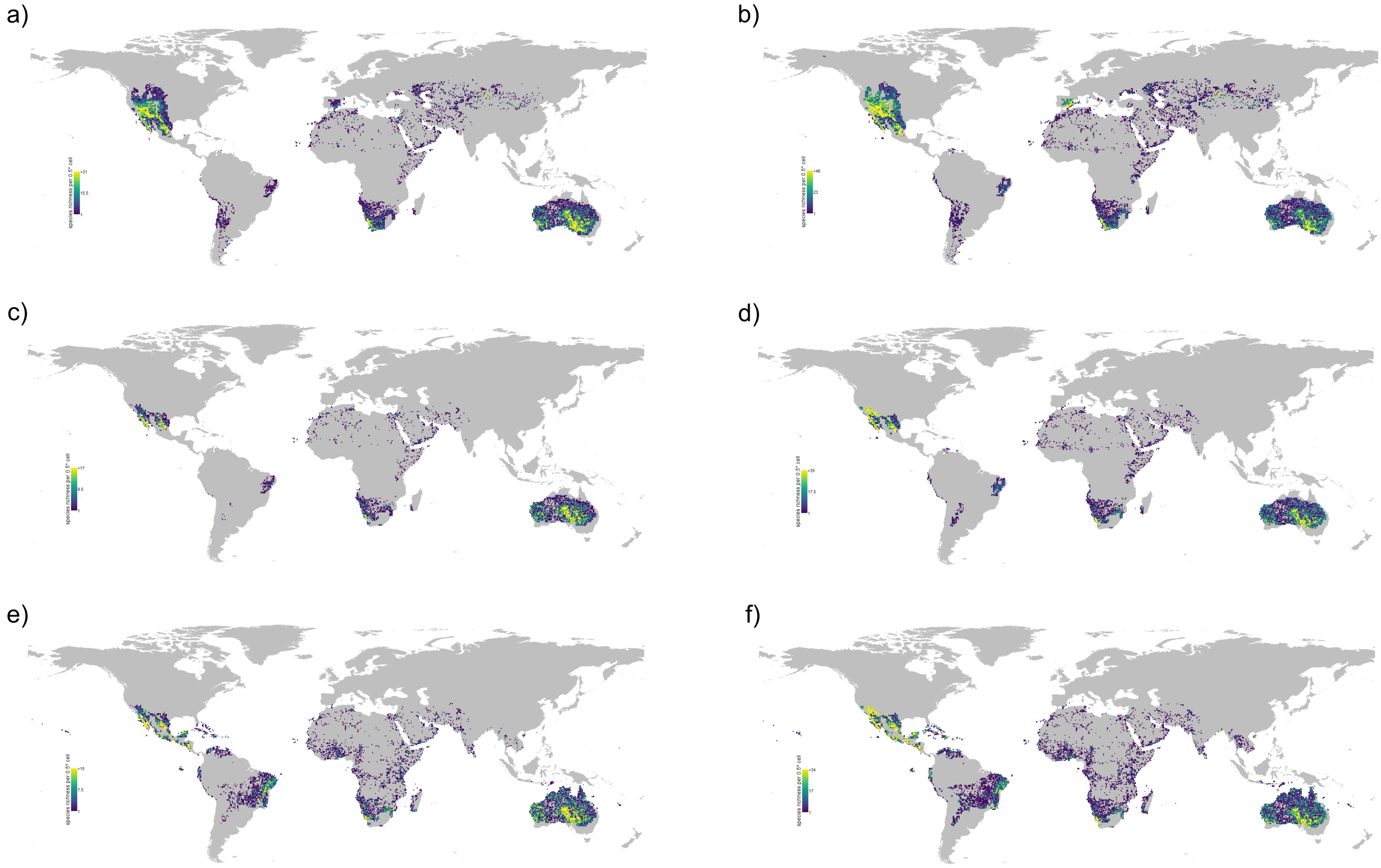

Figure S1. Distribution maps for drought tolerant species in Caryophyllales. a) refers to the main definition i.e. species for which at least 75% of specimens are in arid areas from the Köppen climate categories. b) species for which at least 25% of specimens are in arid areas from the Köppen climate categories. c) species for which 75% of specimens are in hot arid areas from the Köppen climate categories. d) species for which 25% of specimens are in hot arid areas from the Köppen climate categories. e) species for which 75% of specimens are in hot arid areas or hot savannah from the Köppen climate categories. f) species for which 25% of specimens are in hot arid areas or hot savannah from the Köppen climate categories.

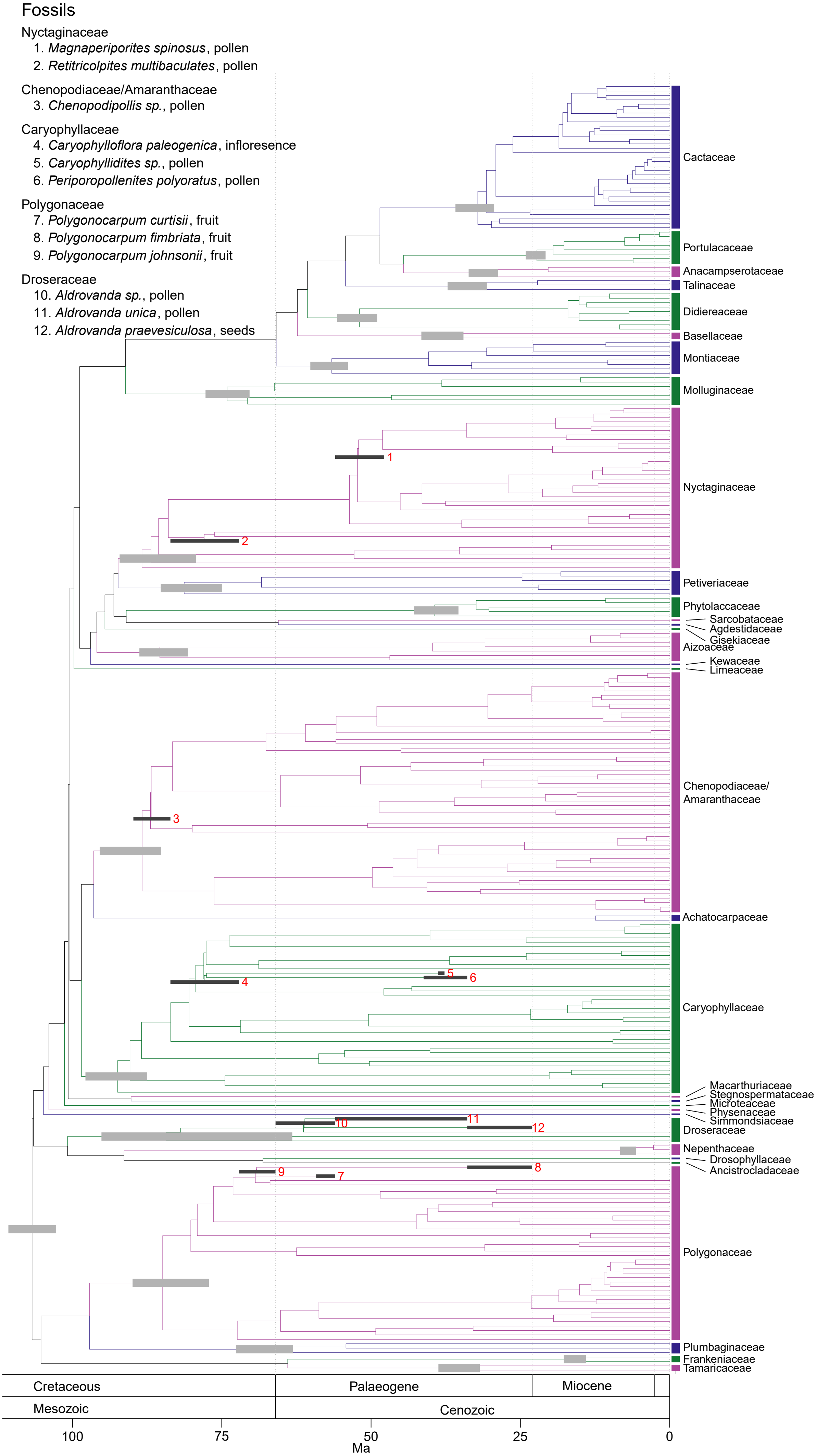

Figure S2. Time-calibrated phylogenetic tree for Caryophyllales constrained to the ASTRAL topology of Walker et al. (2018). Equivalent to Figure 3.

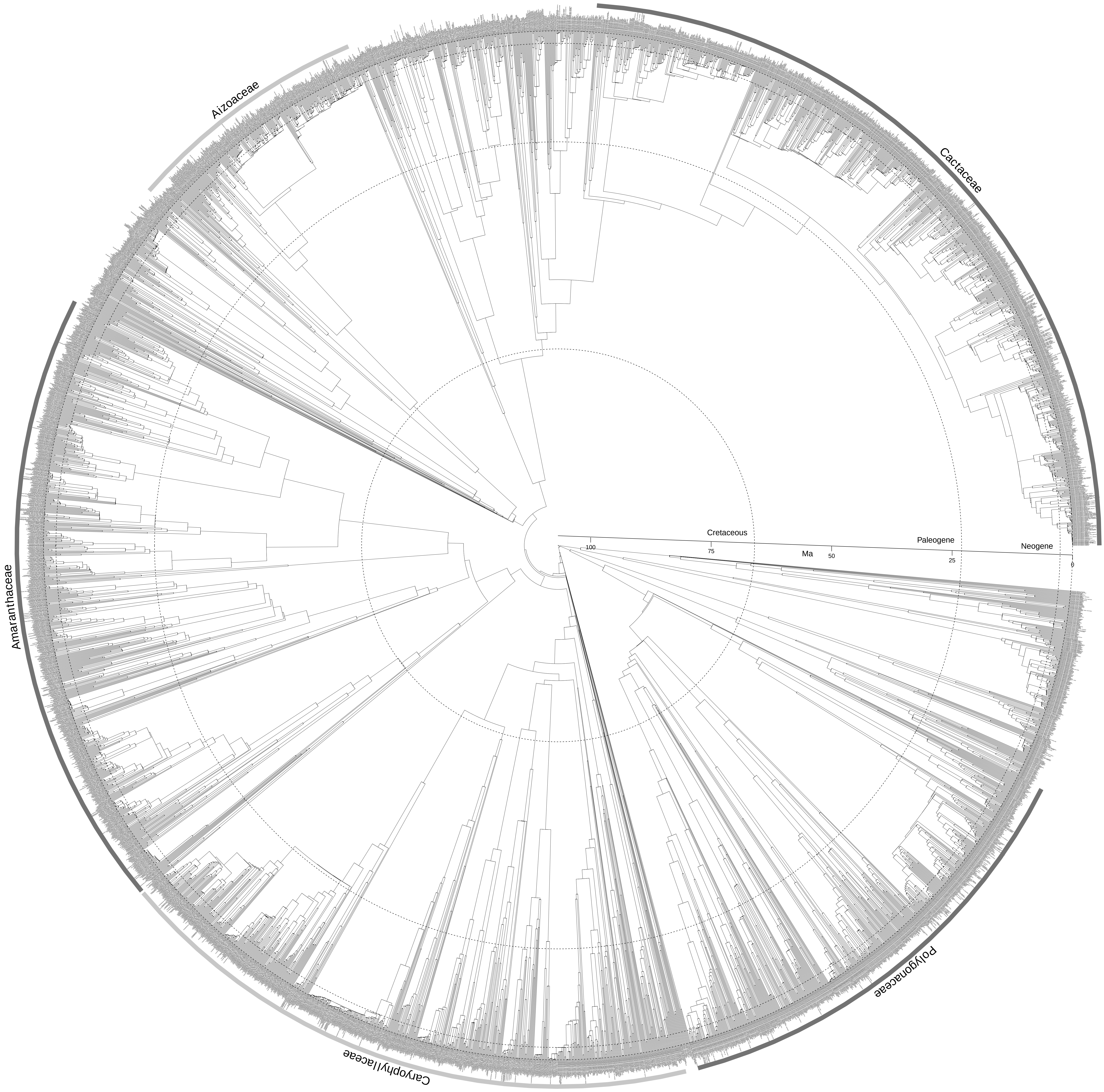

Figure S3. Time-calibrated species-level phylogenetic tree constrained to the RAxML topology of Walker et al. (2018). Tip labels shown.

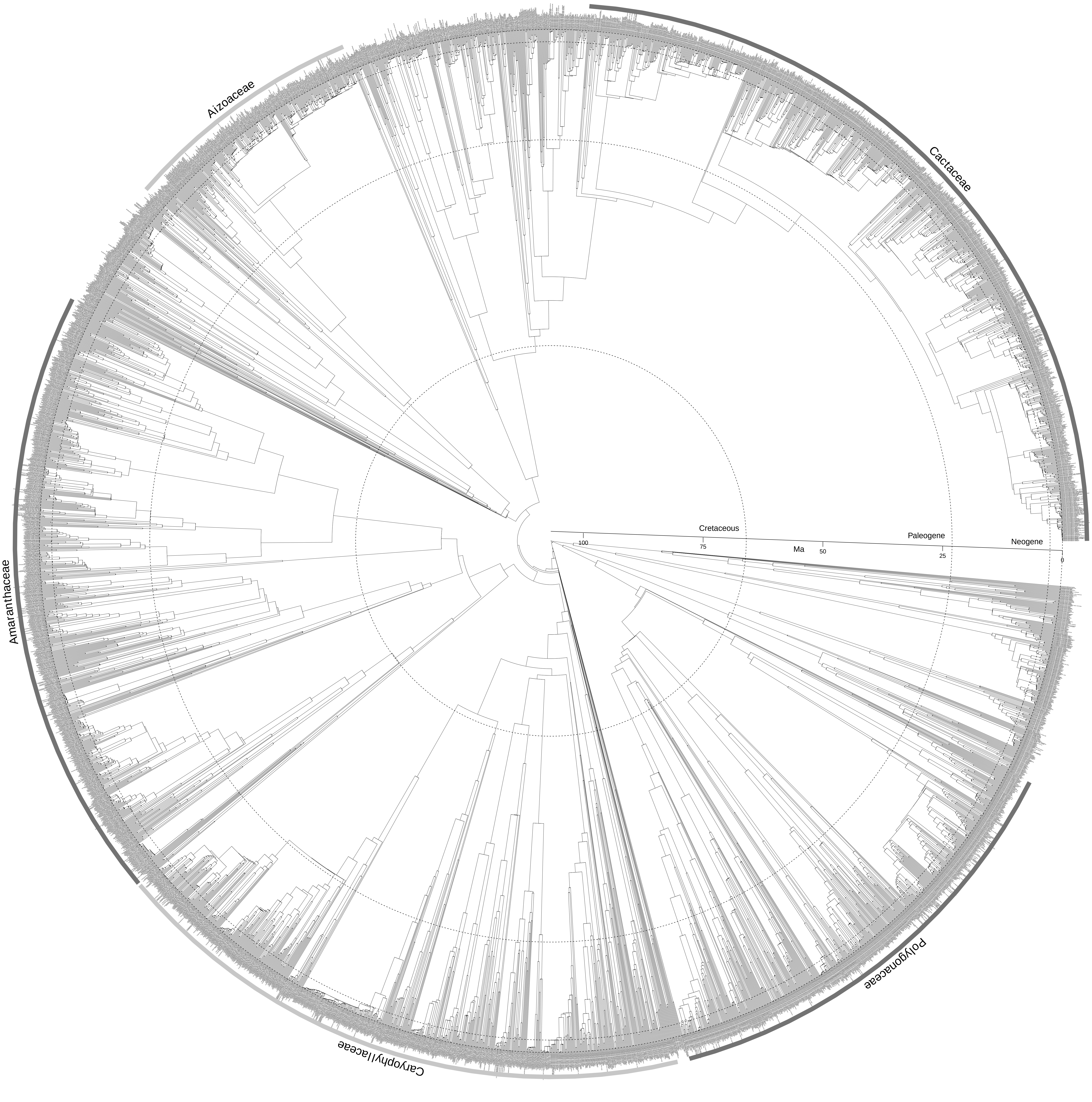

Figure S4. Time-calibrated species-level phylogenetic tree constrained to the ASTRAL topology of Walker et al. (2018). Tip labels shown.

a)

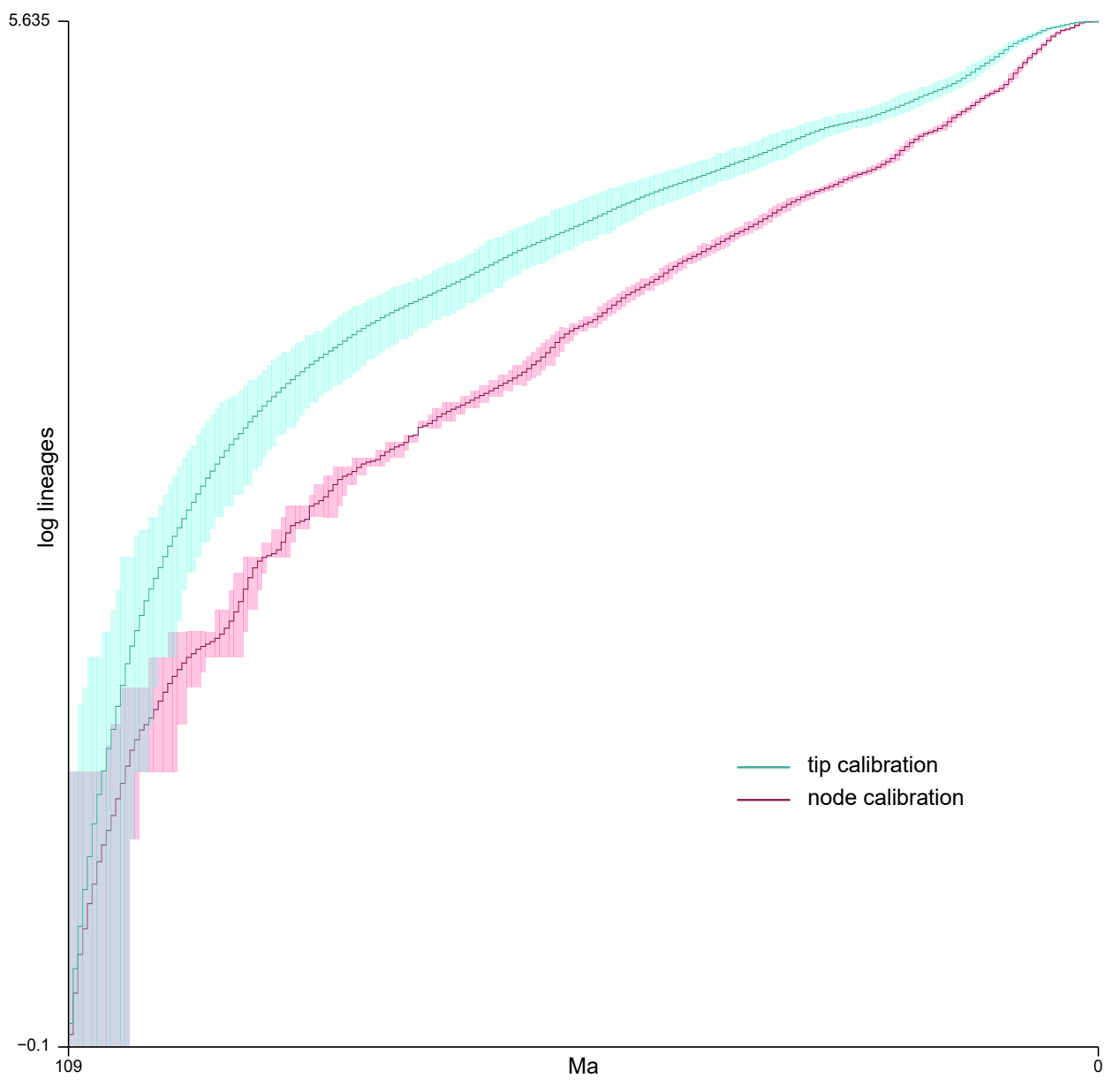

b)

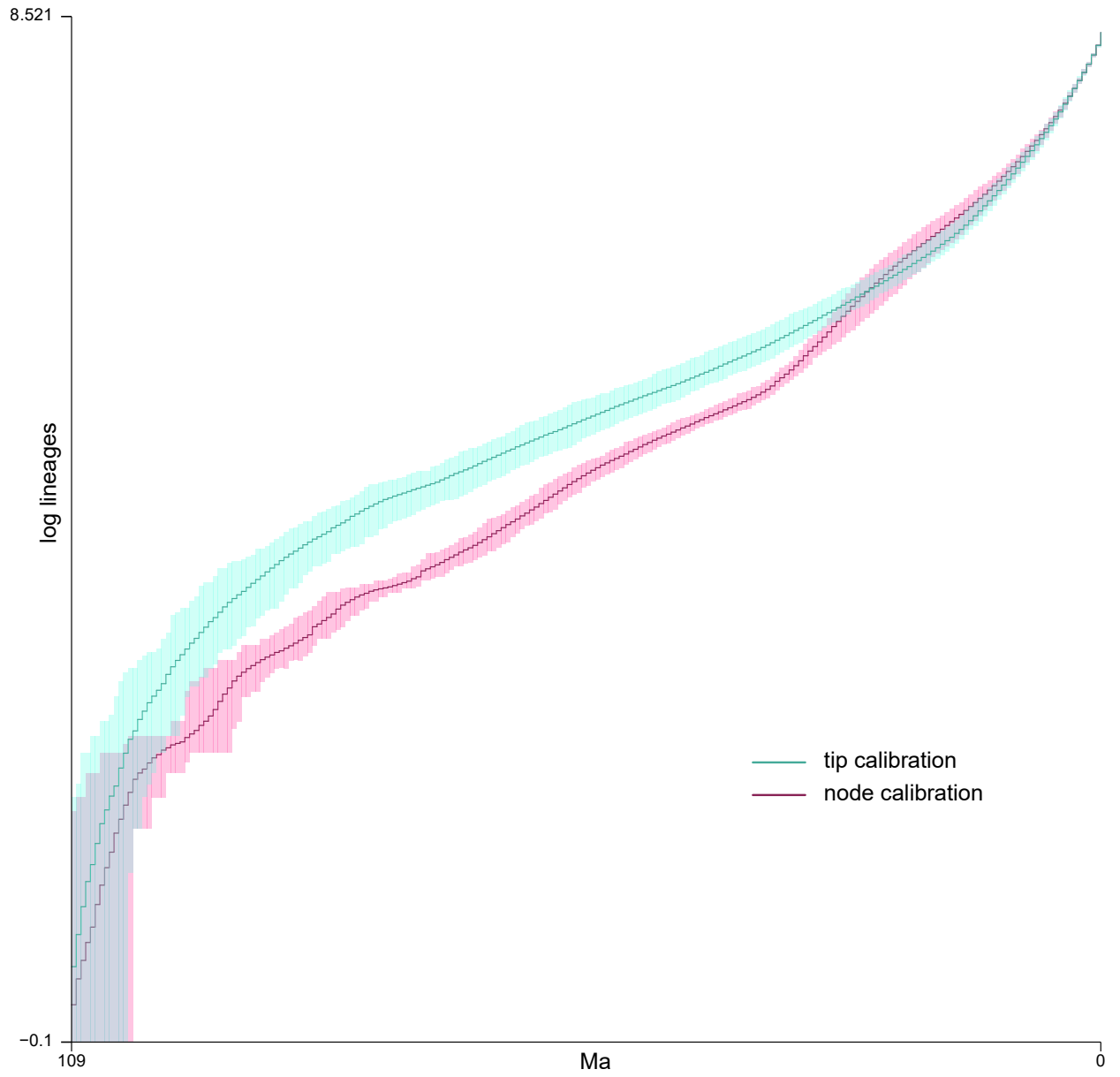

c)

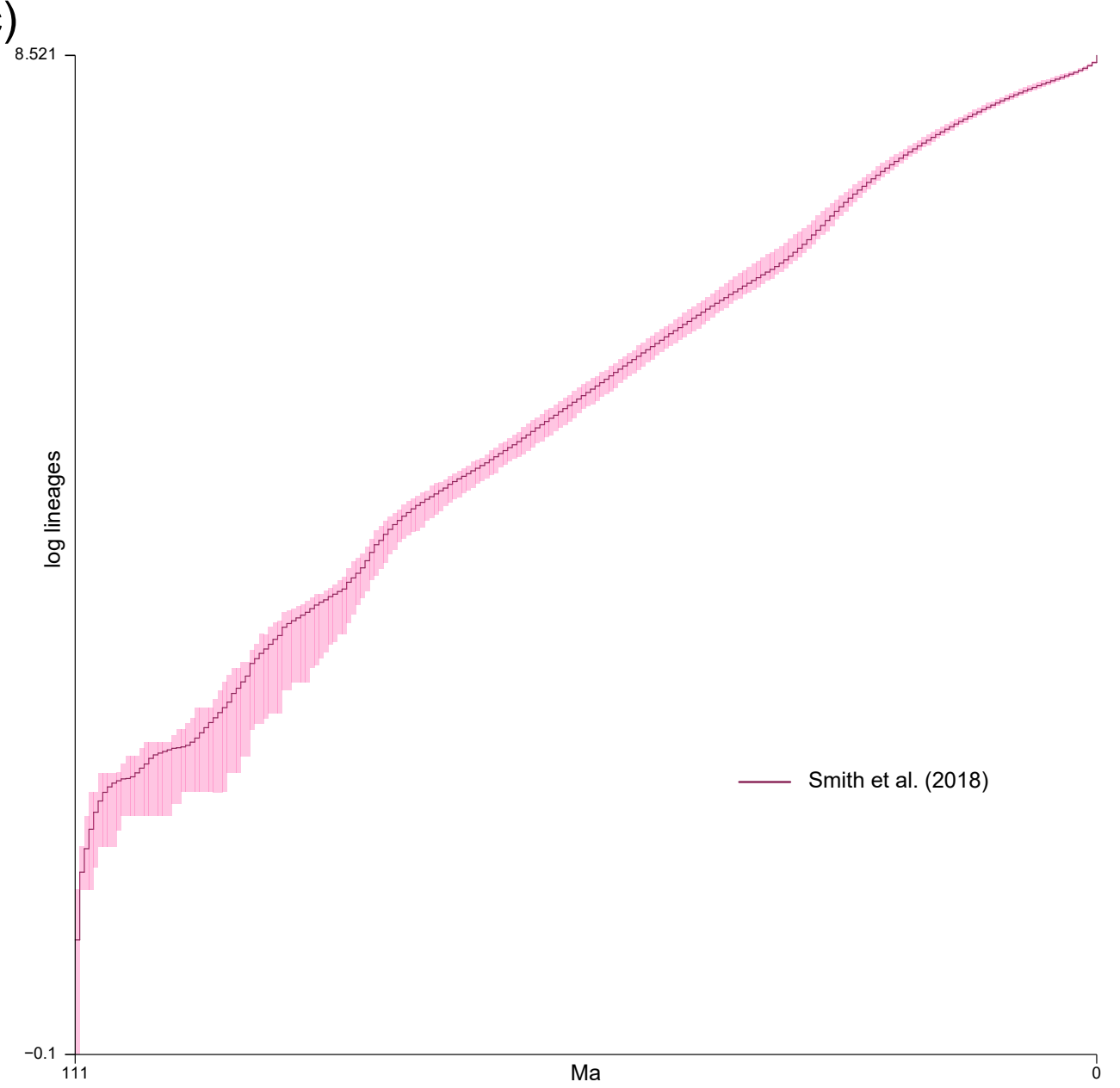

Figure S5. Lineages through time plots for different dated phylogenetic trees. Line shows the mean number of lineages across all bootstraps at a given point in time, shaded area refers to the 95% confidence interval for the number of lineages at that time. These plots are intended to summarise differences in age estimates in each dated phylogenetic tree. a) Comparison of node-dating and tip-dating divergence time estimates in the transcriptomic dataset. b) Comparison of node-dating and tip-dating divergence time estimates in the species-level dataset. c) Equivalent plot for the phylogenetic tree estimated in Smith et al. (2018).

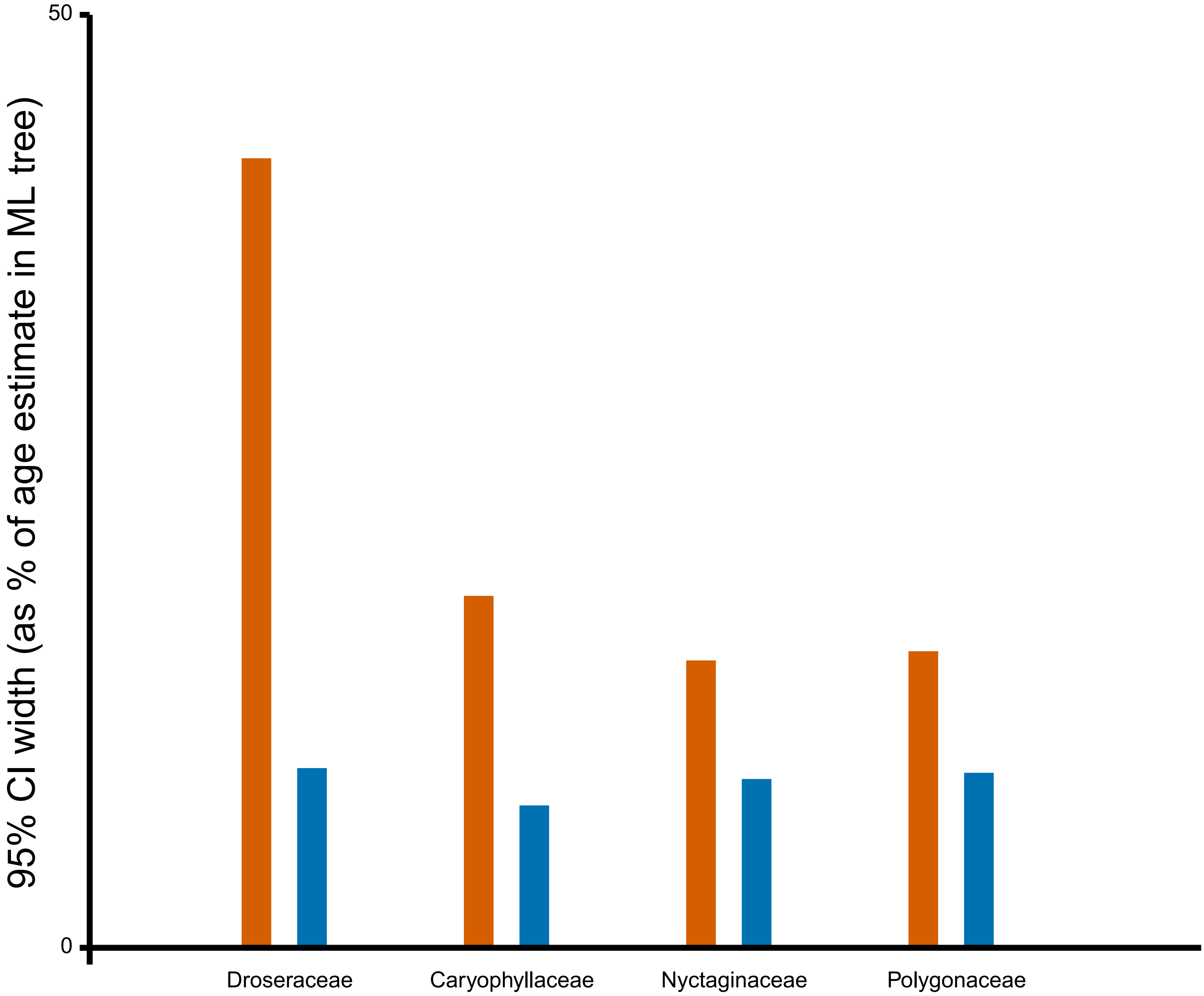

Figure S6. Comparison of 95% CI widths for family crown age estimates when all fossils are included (orange bar) compared to when all fossils for that family are excluded (blue bar).

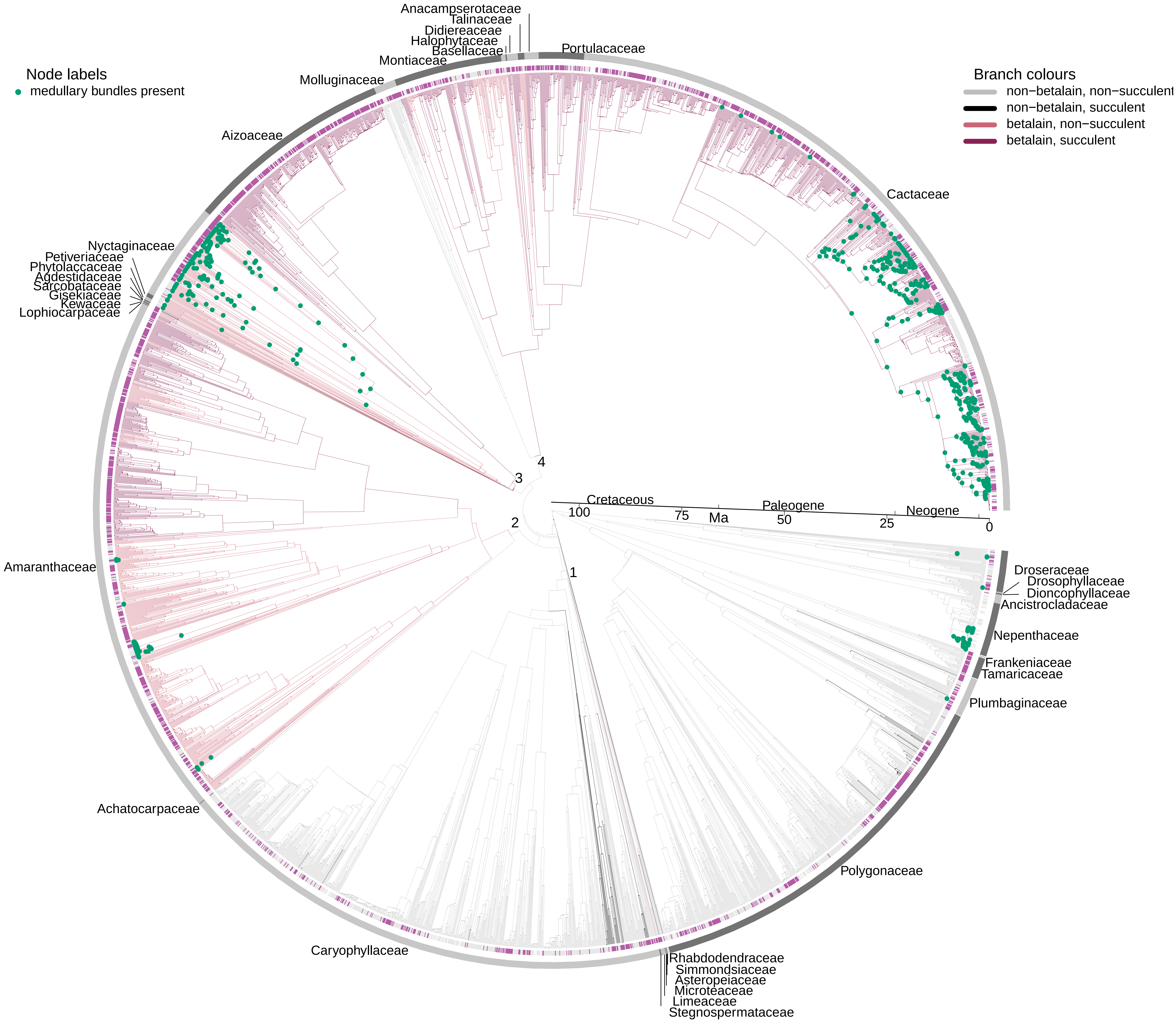

Figure S7. Equivalent to Figure 4. Drought tolerant species defined as those with at least 25% of specimens in arid climates as per Köppen climate categories.

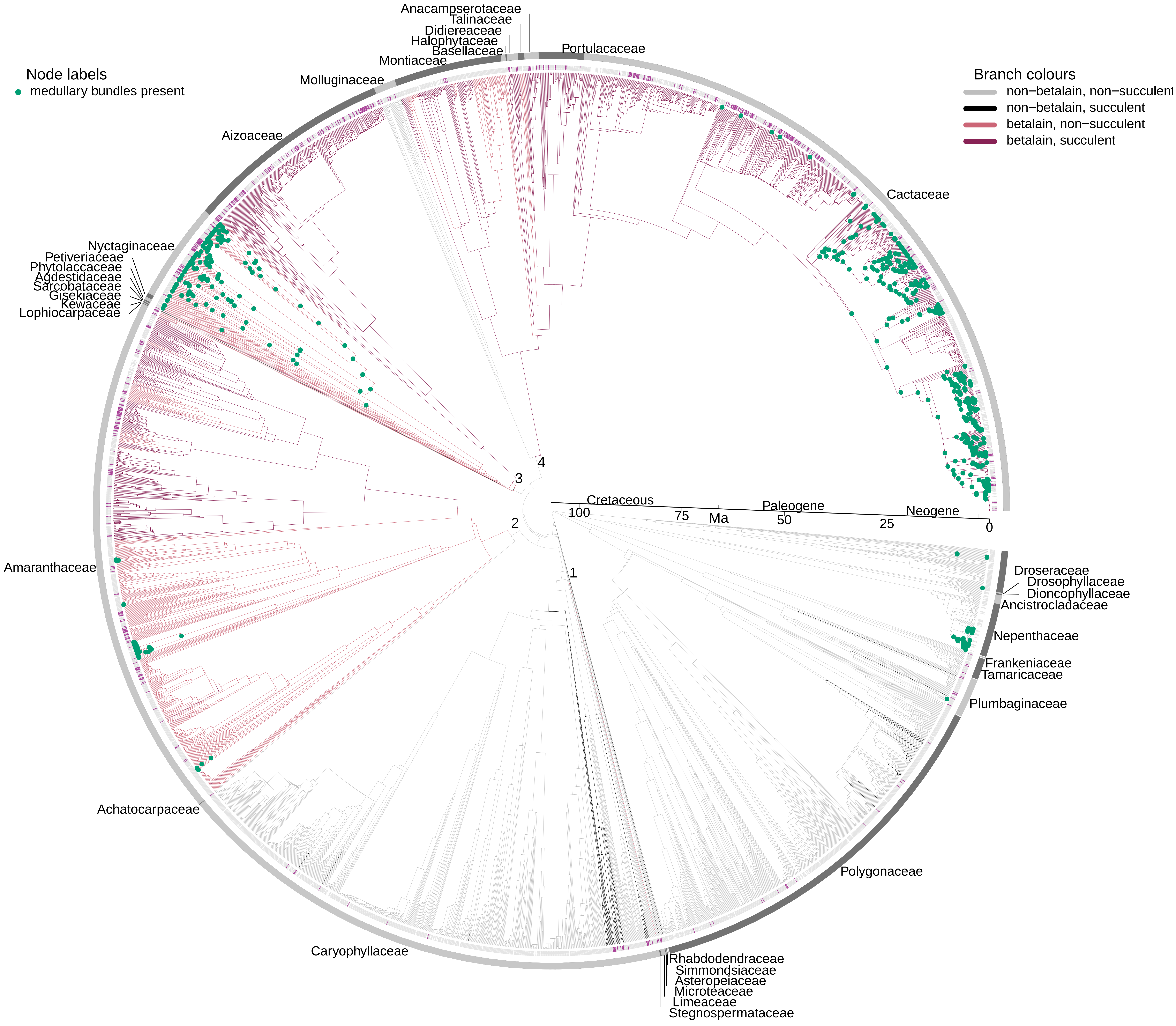

Figure S8. Equivalent to Figure 4. Drought tolerant species defined as those with at least 75% of specimens in hot arid climates as per Köppen climate categories.

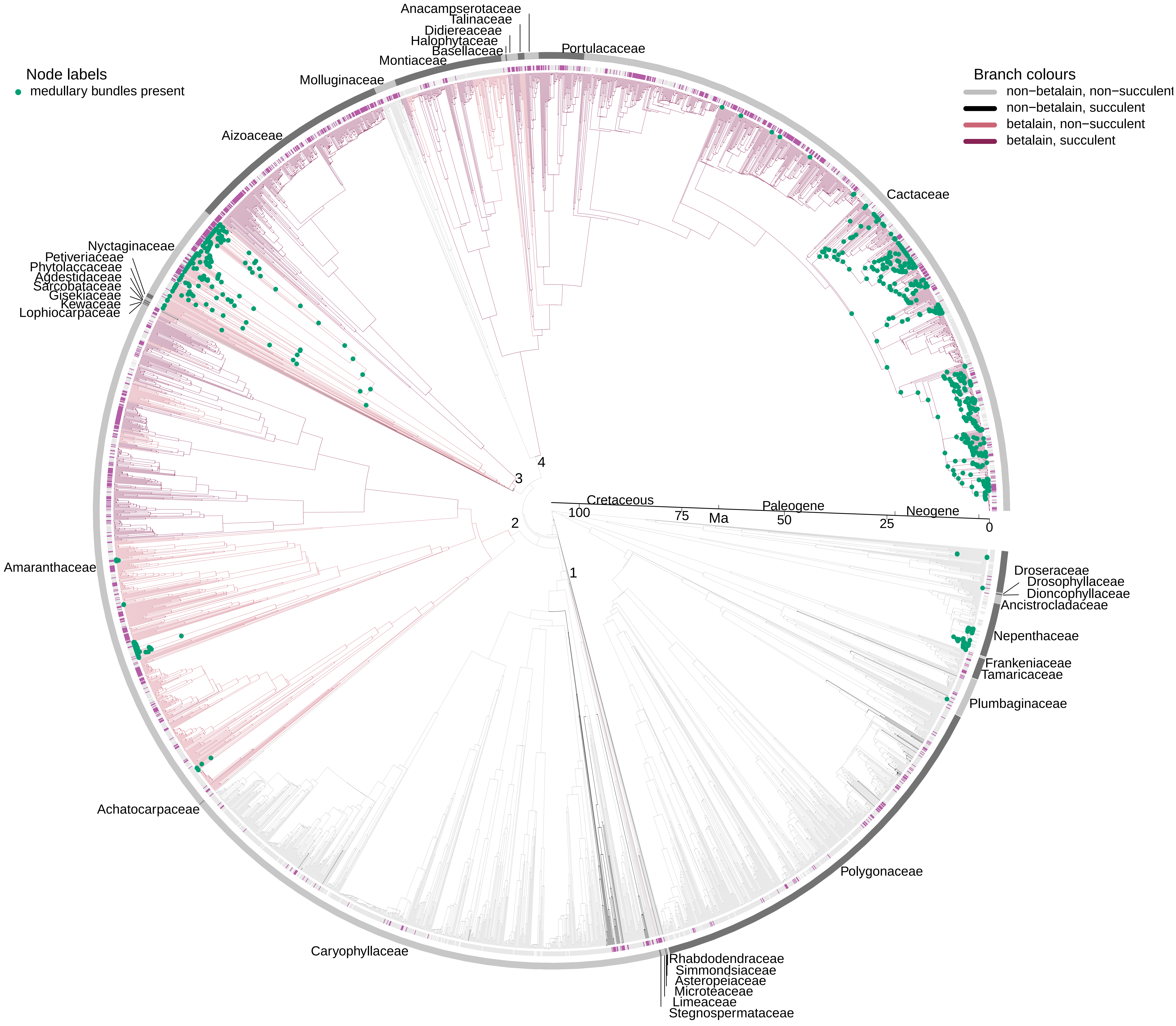

Figure S9. Equivalent to Figure 4. Drought tolerant species defined as those with at least 25% of specimens in hot arid climates as per Köppen climate categories.

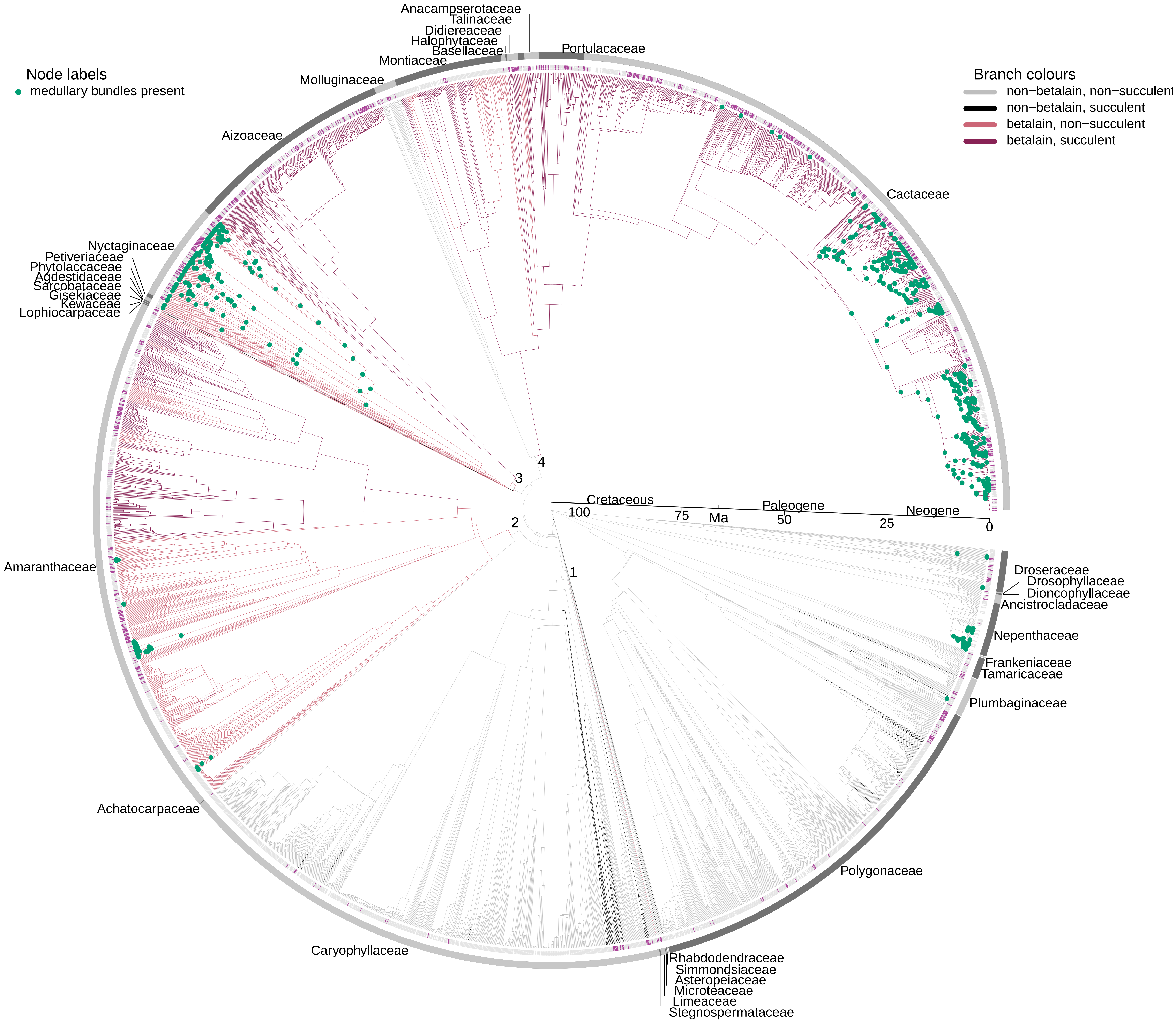

Figure S10. Equivalent to Figure 4. Drought tolerant species defined as those with at least 75% of specimens in hot arid climates or hot savannah as per Köppen climate categories.

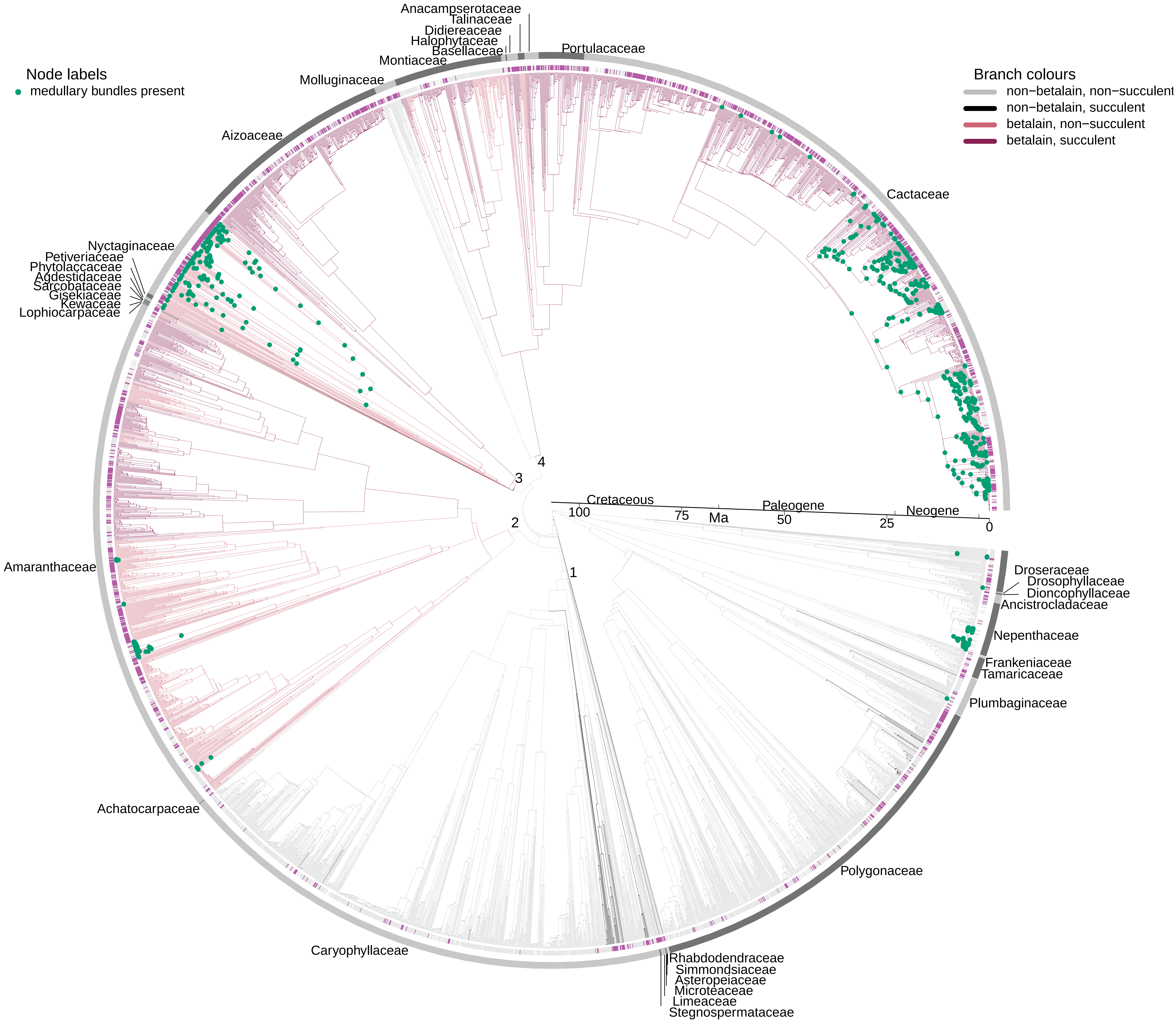

Figure S11. Equivalent to Figure 4. Drought tolerant species defined as those with at least 25% of specimens in hot arid climates or hot savannah as per Köppen climate categories.

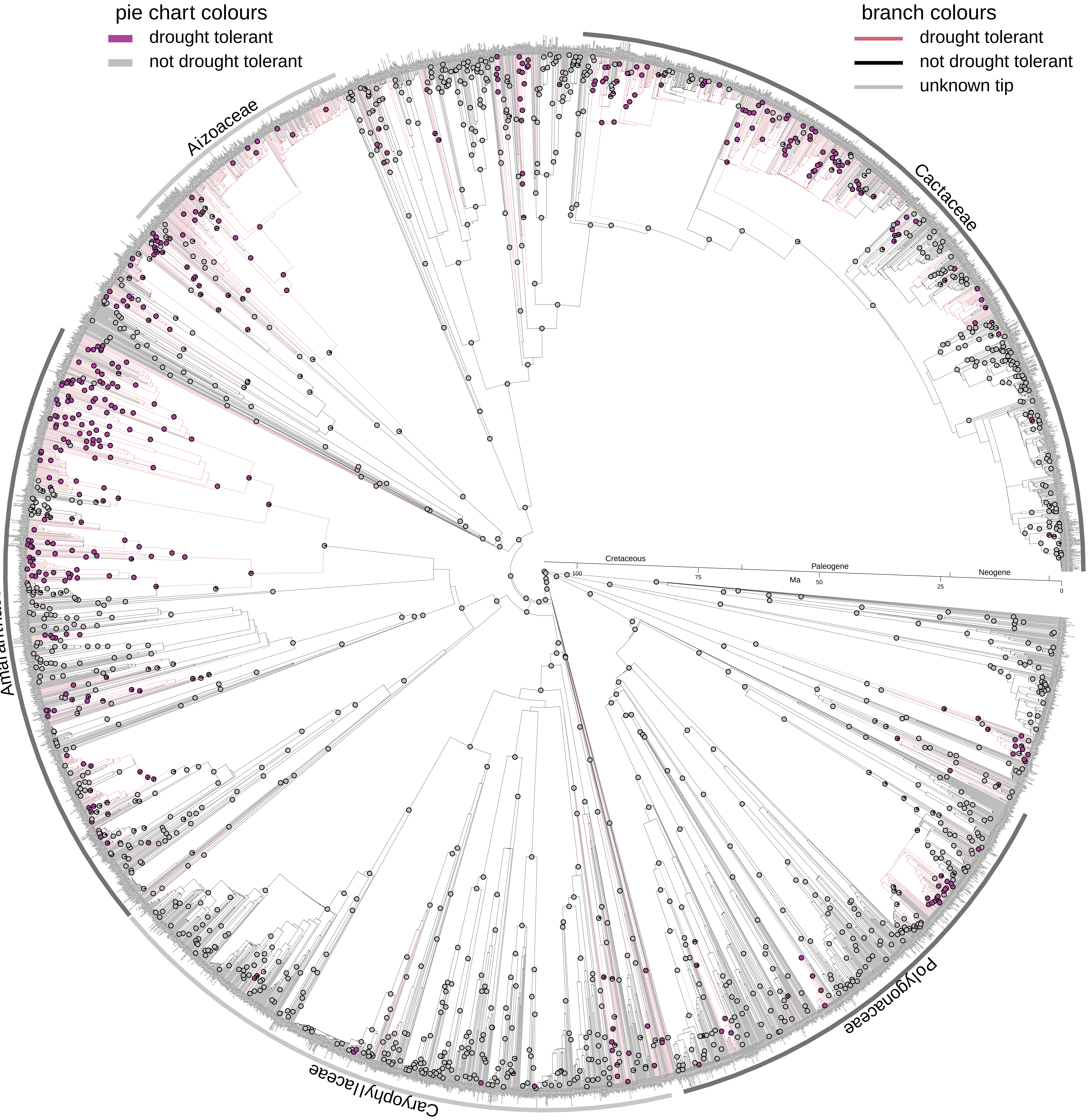

Figure S12. Reconstruction of drought tolerance with the best fitting model (ARD), where species are defined as drought tolerant if at least 75% of specimens are in arid climates as per Köppen climate categories.

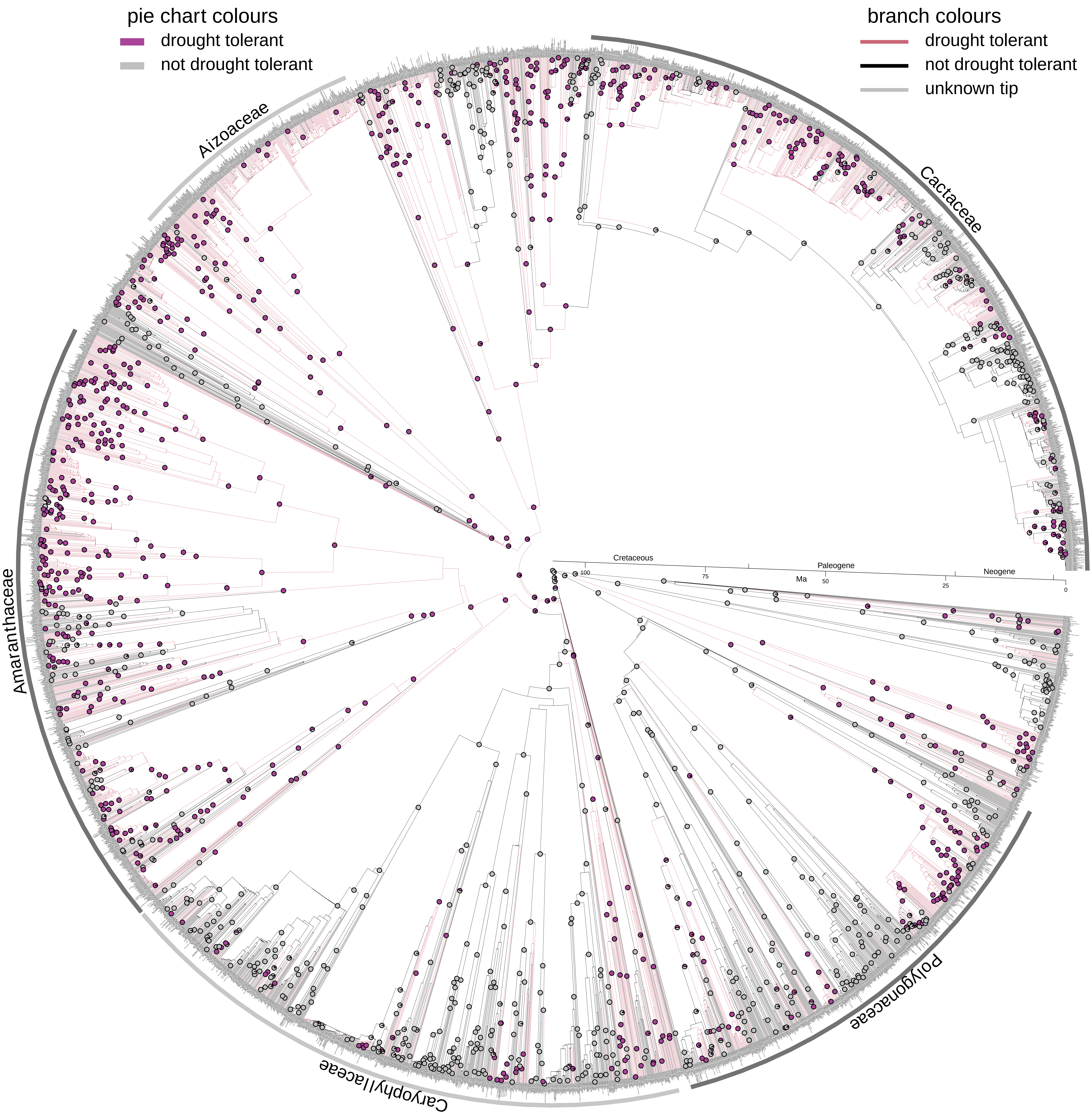

Figure S13. Reconstruction of drought tolerance with the best fitting model (ER), where species are defined as drought tolerant if at least 25% of specimens are in arid climates as per Köppen climate categories.

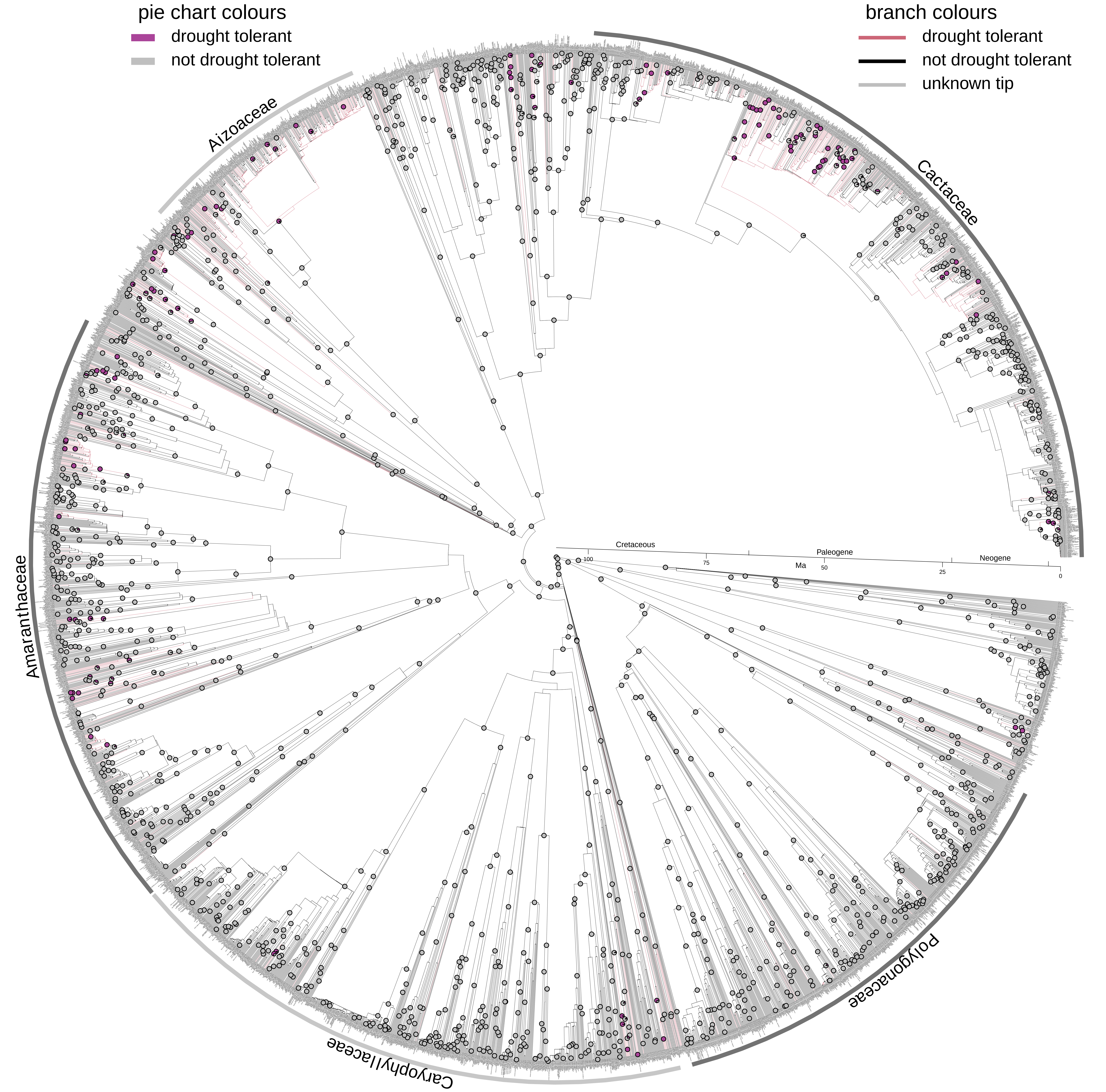

Figure S14. Reconstruction of drought tolerance with the best fitting model (ARD), where species are defined as drought tolerant if at least 75% of specimens are in hot arid climates as per Köppen climate categories.

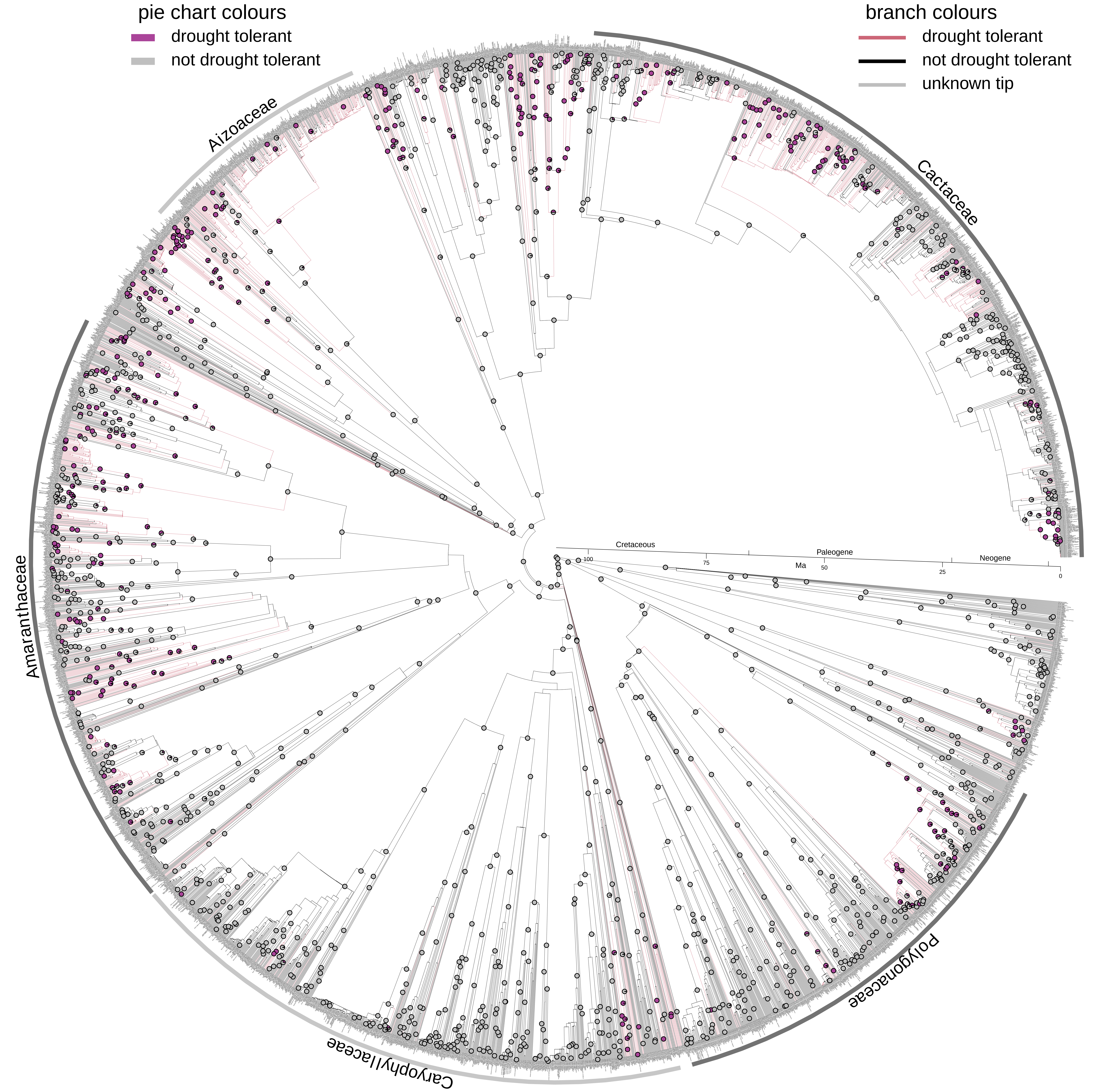

Figure S15. Reconstruction of drought tolerance with the best fitting model (ARD), where species are defined as drought tolerant if at least 25% of specimens are in hot arid climates as per Köppen climate categories.

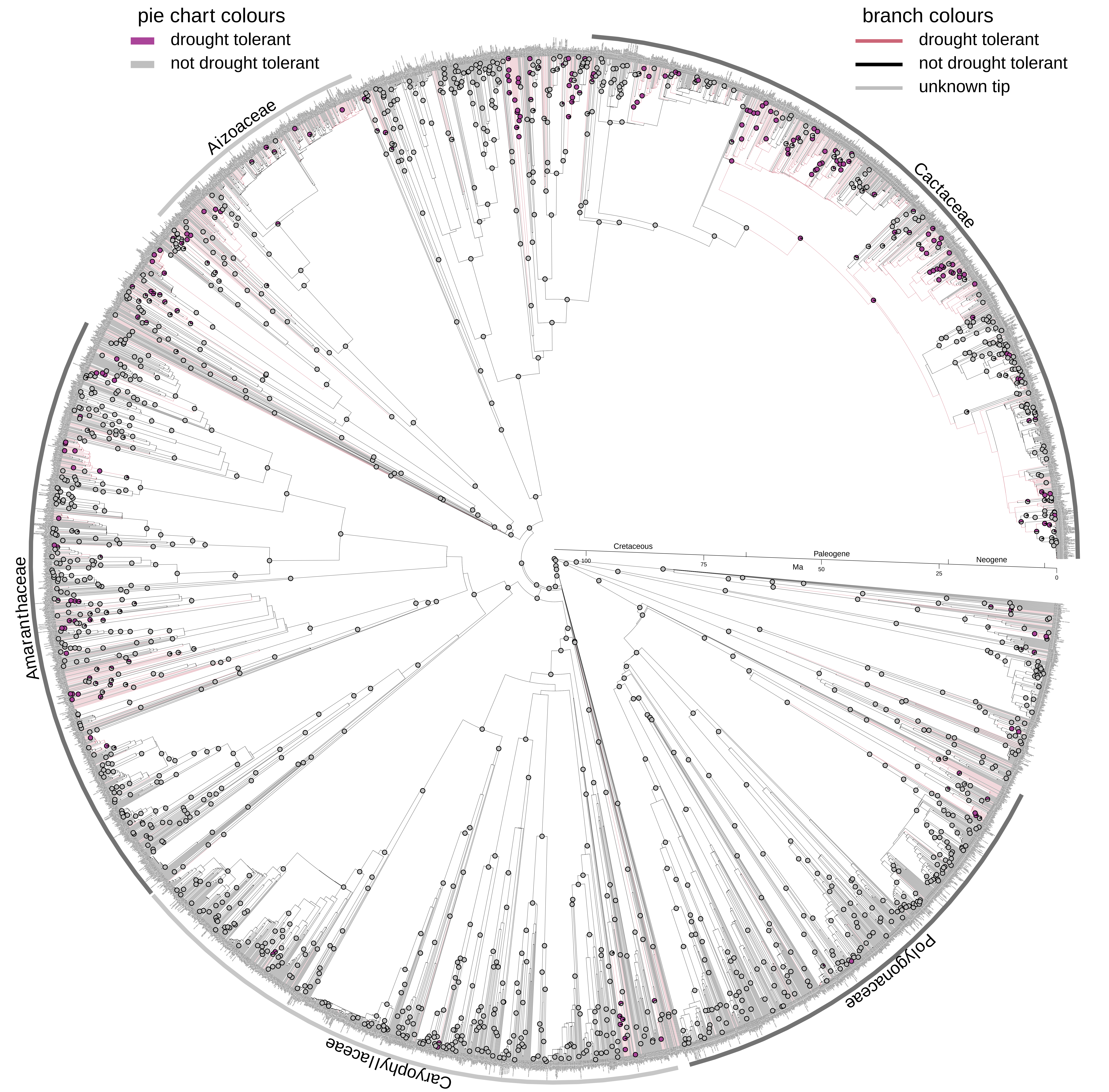

Figure S16. Reconstruction of drought tolerance with the best fitting model (ARD), where species are defined as drought tolerant if at least 75% of specimens are in hot arid or hot savannah climates as per Köppen climate categories.

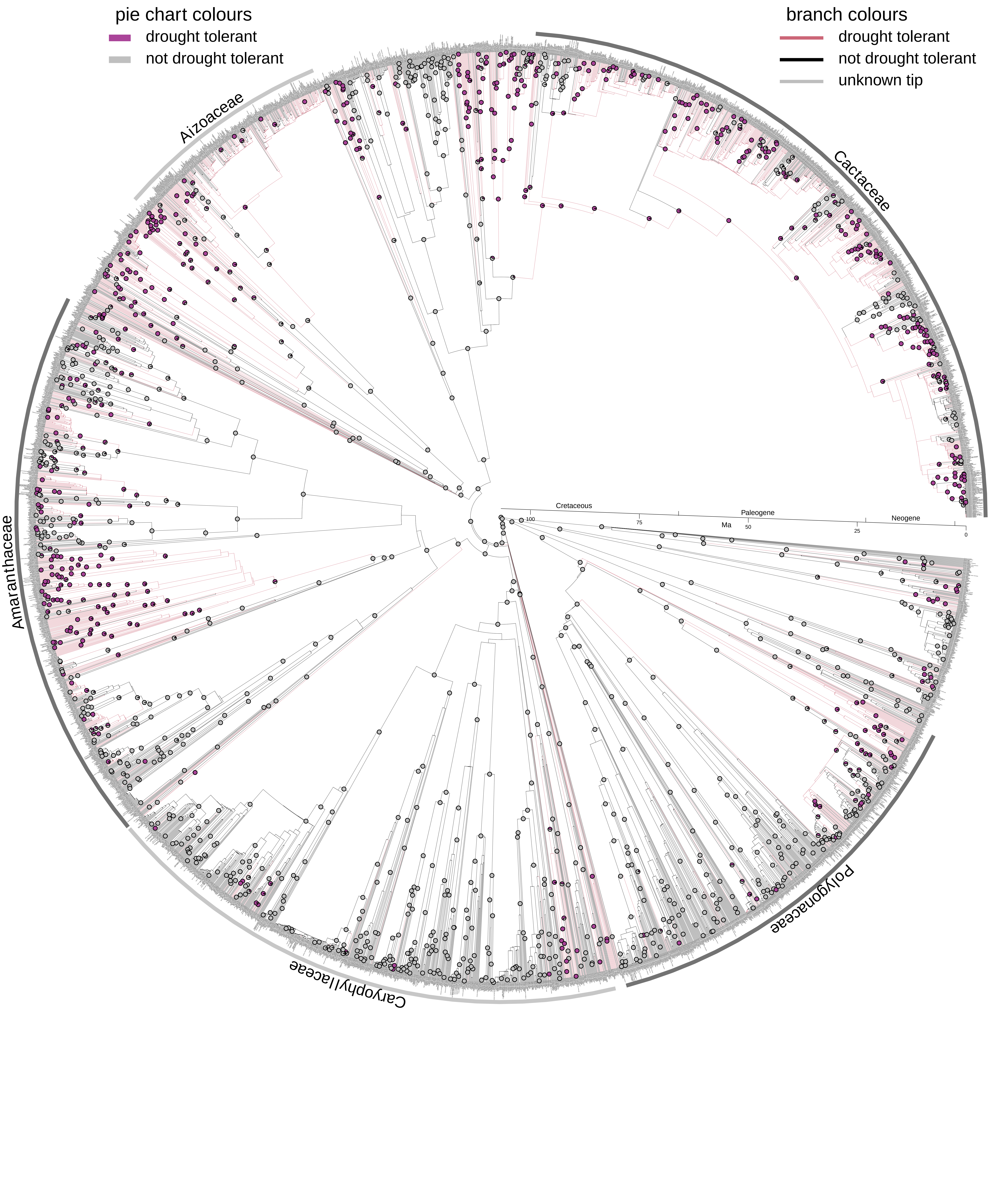

Figure S17. Reconstruction of drought tolerance with the best fitting model (ARD), where species are defined as drought tolerant if at least 25% of specimens are in hot arid or hot savannah climates as per Köppen climate categories.

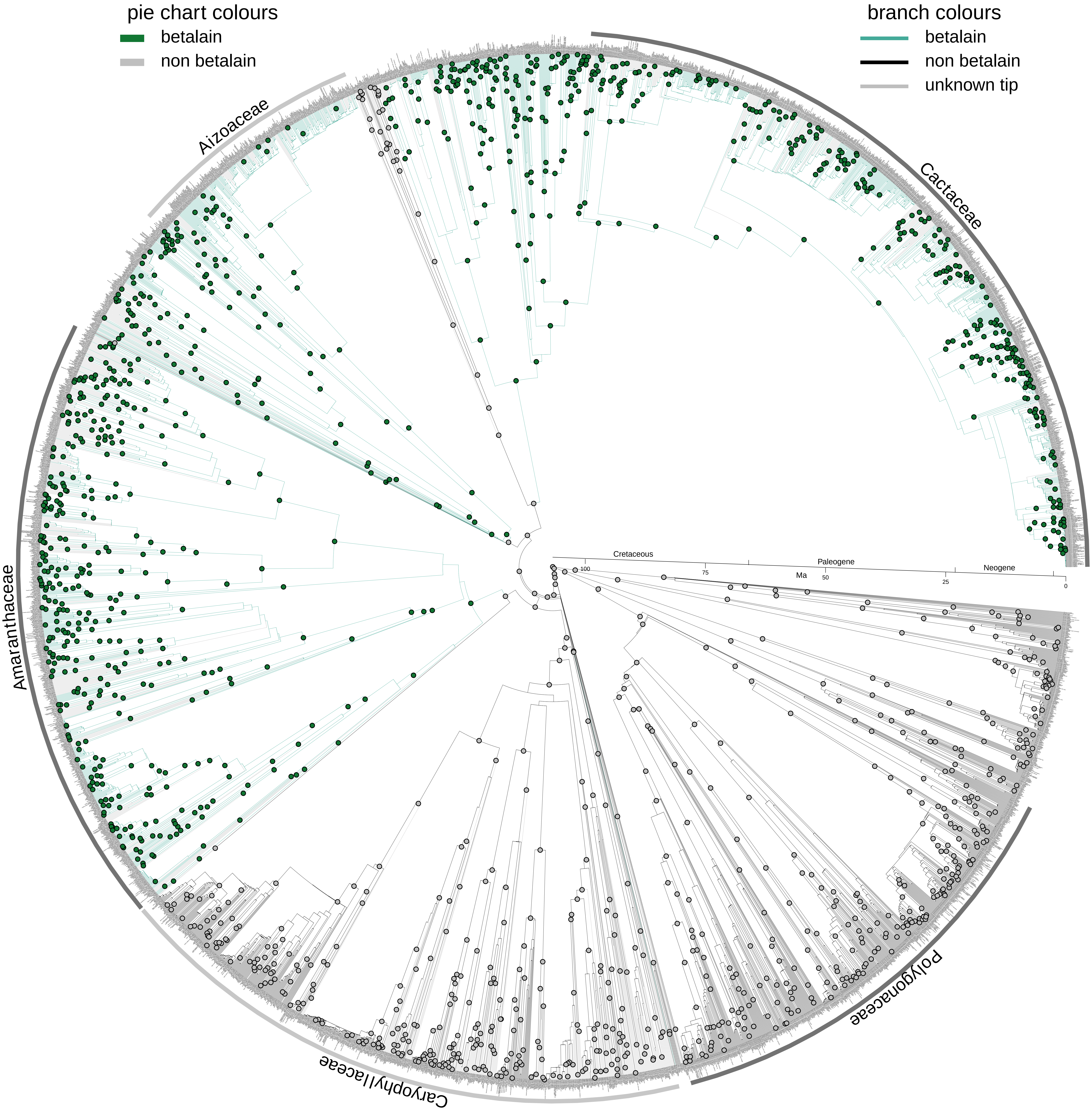

Figure S18. Reconstruction of evolution of betalain pigmentation on the species-level phylogenetic tree constrained to the RAxML topology of Walker et al. (2018) and with an irreversible model. Pie charts refer to node states reconstructed across all bootstrap trees.

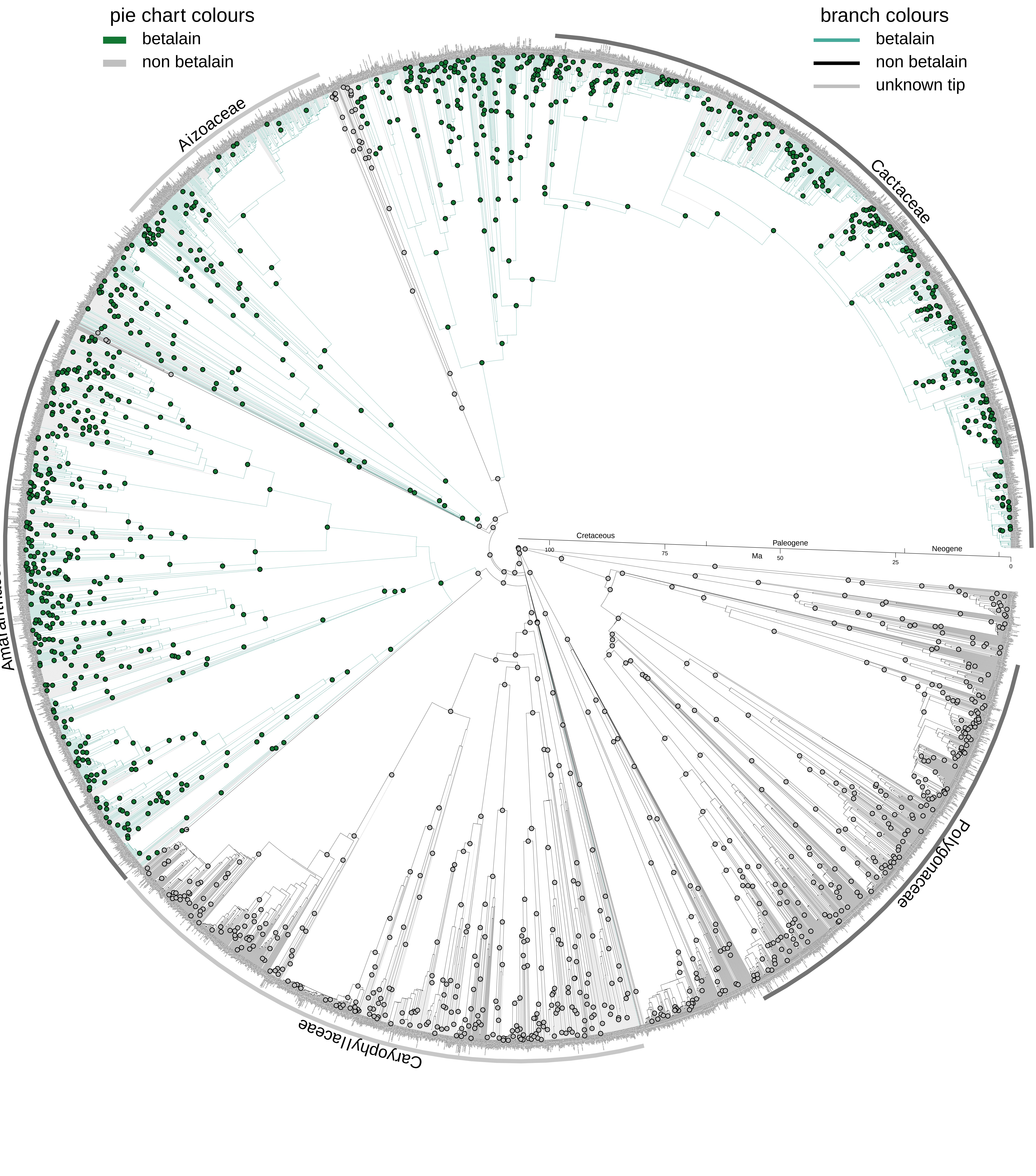

Figure S19. Reconstruction of evolution of betalain pigmentation on the species-level phylogenetic tree constrained to the ASTRAL topology of Walker et al. (2018) and with an irreversible model. Pie charts refer to node states reconstructed across all bootstrap trees.

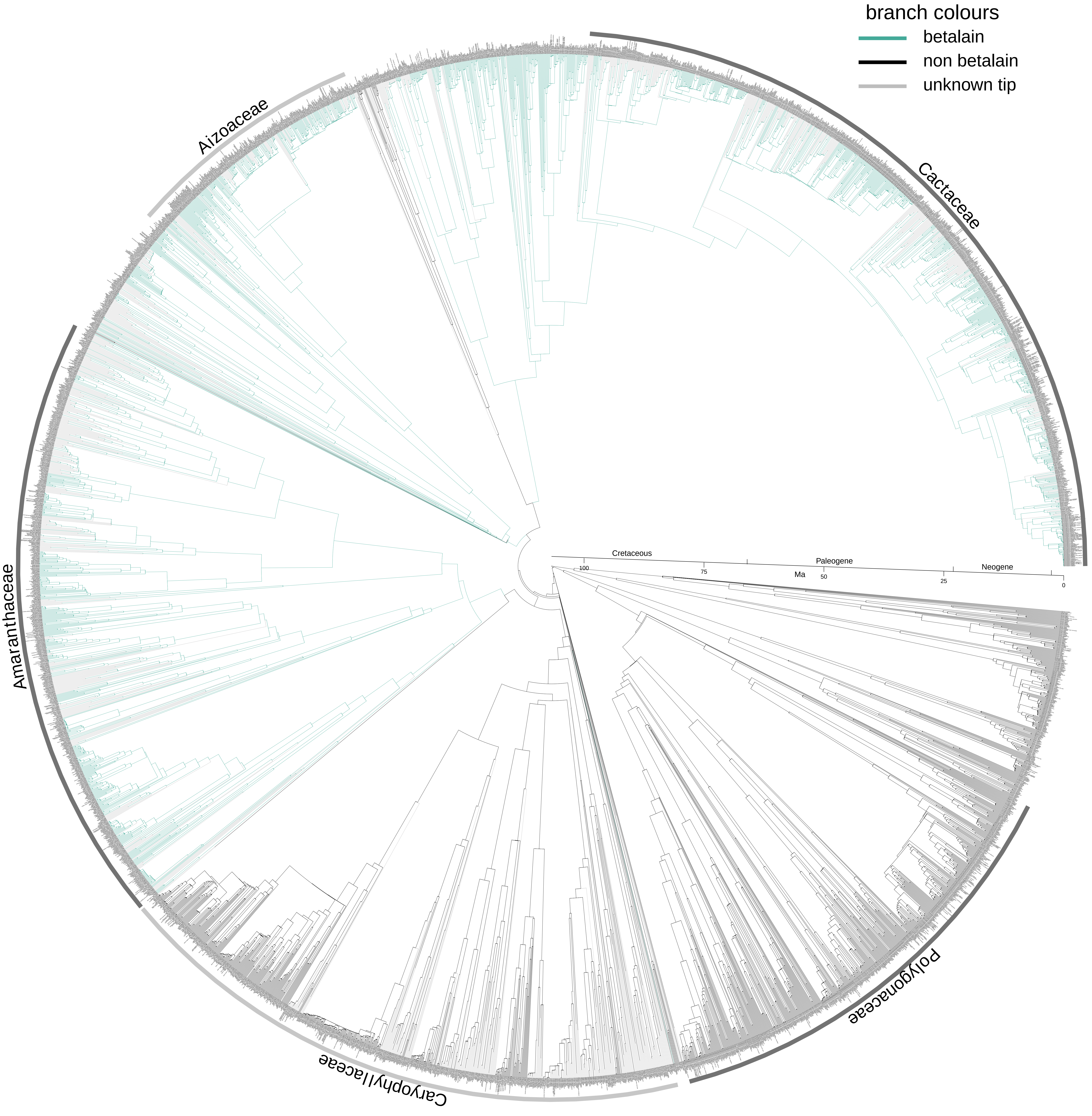

Figure S20. Reconstruction of evolution of betalain pigmentation on the species-level phylogenetic tree constrained to the RAxML topology of Walker et al. (2018) and with an ER model.

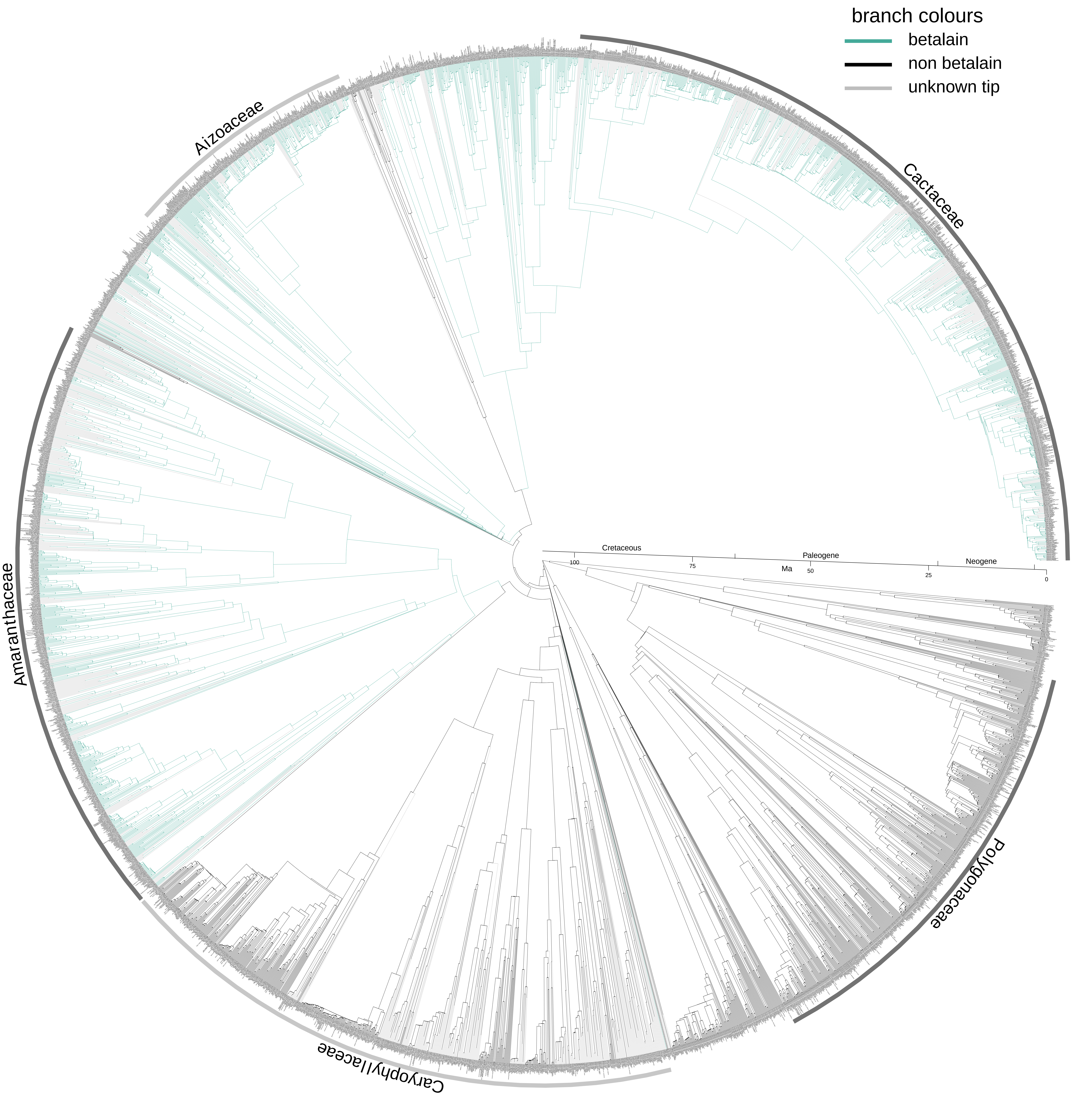

Figure S21. Reconstruction of evolution of betalain pigmentation on the species-level phylogenetic tree constrained to the ASTRAL topology of Walker et al. (2018) and with an ER model.

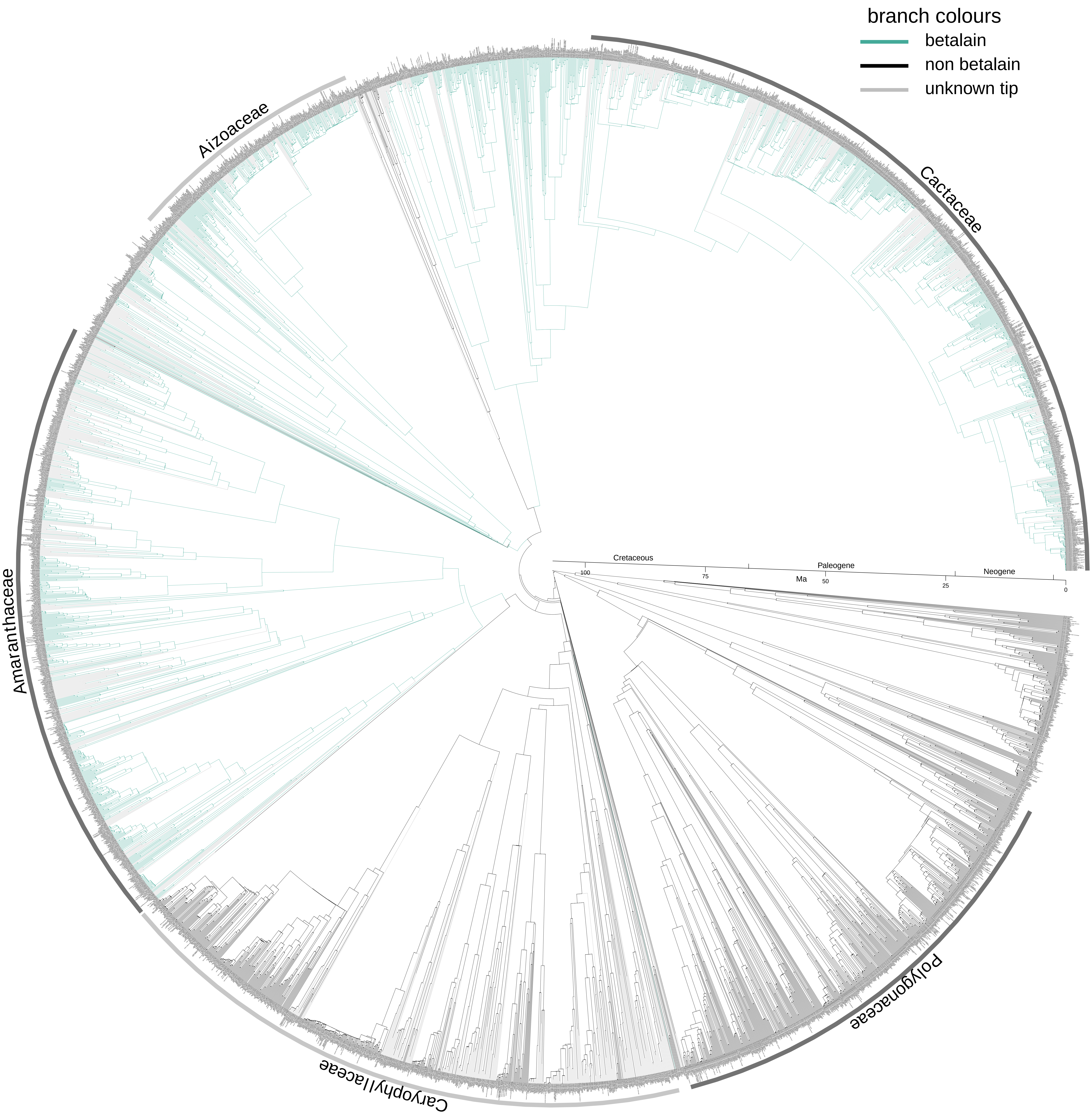

Figure S22. Reconstruction of evolution of betalain pigmentation on the species-level phylogenetic tree constrained to the RAxML topology of Walker et al. (2018) and with an ARD model.

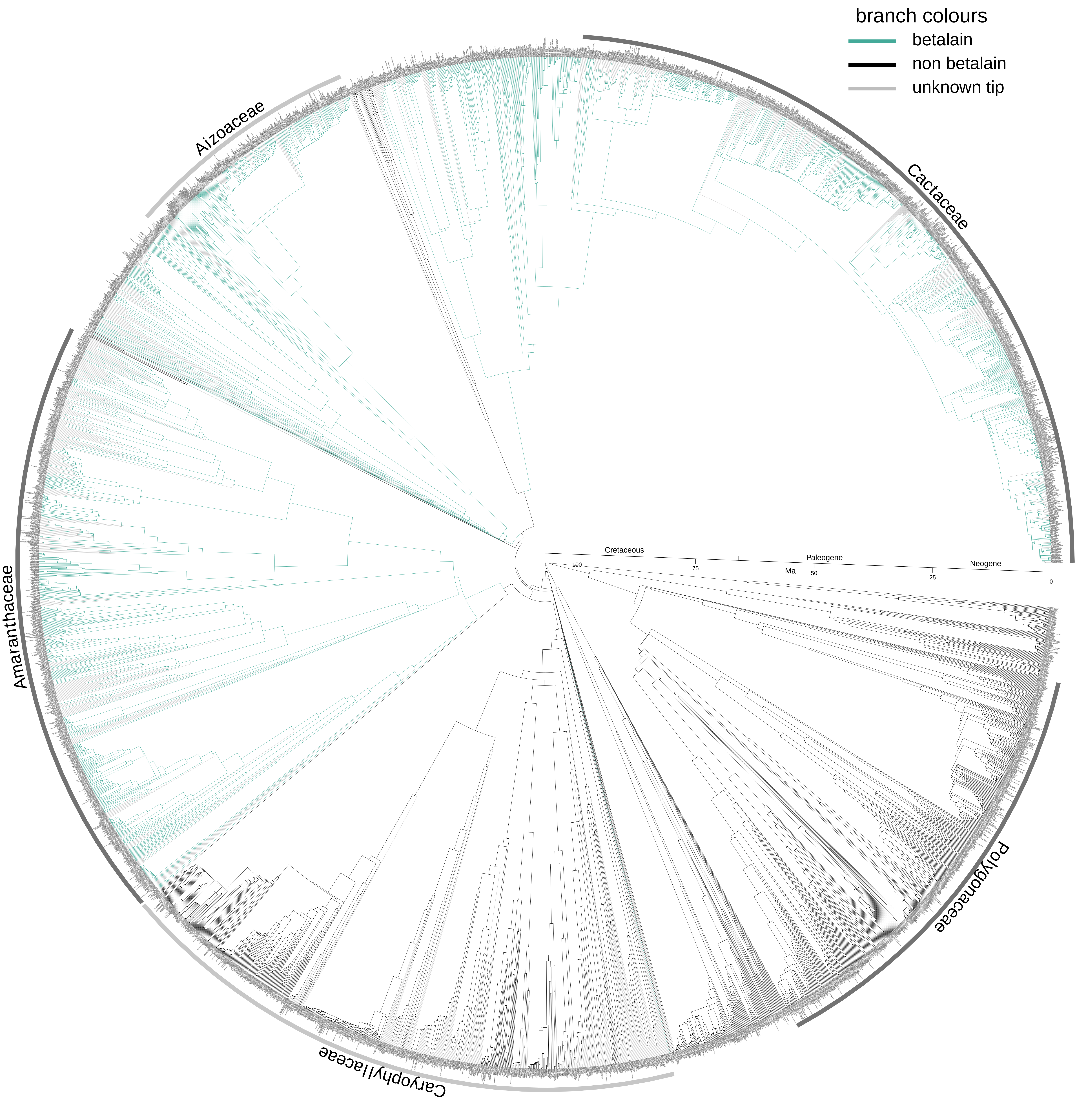

Figure S23. Reconstruction of evolution of betalain pigmentation on the species-level phylogenetic tree constrained to the ASTRAL topology of Walker et al. (2018) and with an ARD model.

0 = not drought tolerant + no betalains, 1 = not drought tolerant + betalains
2 = drought tolerant + no betalains, 3 = drought tolerant + betalains

		hidden state 1				hidden state 2				hidden state 3				hidden state 4			
		0	1	2	3	0	1	2	3	0	1	2	3	0	1	2	3
hidden state 1	0		0.01881	0.25		0.00049				0.02269				0.00164			
	1				0.25		0.00049				0.02269				0.00164		
	2	0.08712			0.01881			0.00049				0.02269				0.00164	
	3		0.08712						0.00049				0.02269				0.00164
hidden state 2	0	2e-05						0.01618		0.00133				2e-05			
	1		2e-05						0.01618		0.00133				2e-05		
	2			2e-05		0.25						0.00133				2e-05	
	3				2e-05		0.25						0.00133				2e-05
hidden state 3	0	2e-05				0.00719						0.1446		0.01044			
	1		2e-05				0.00719						0.1446		0.01044		
	2			2e-05				0.00719		0.25						0.01044	
	3				2e-05				0.00719		0.25						0.01044
hidden state 4	0	2e-05				2e-05				0.00326						0.00037	
	1		2e-05				2e-05				0.00326						0.00037
	2			2e-05				2e-05				0.00326		0.01106			
	3				2e-05				2e-05				0.00326		0.01106		

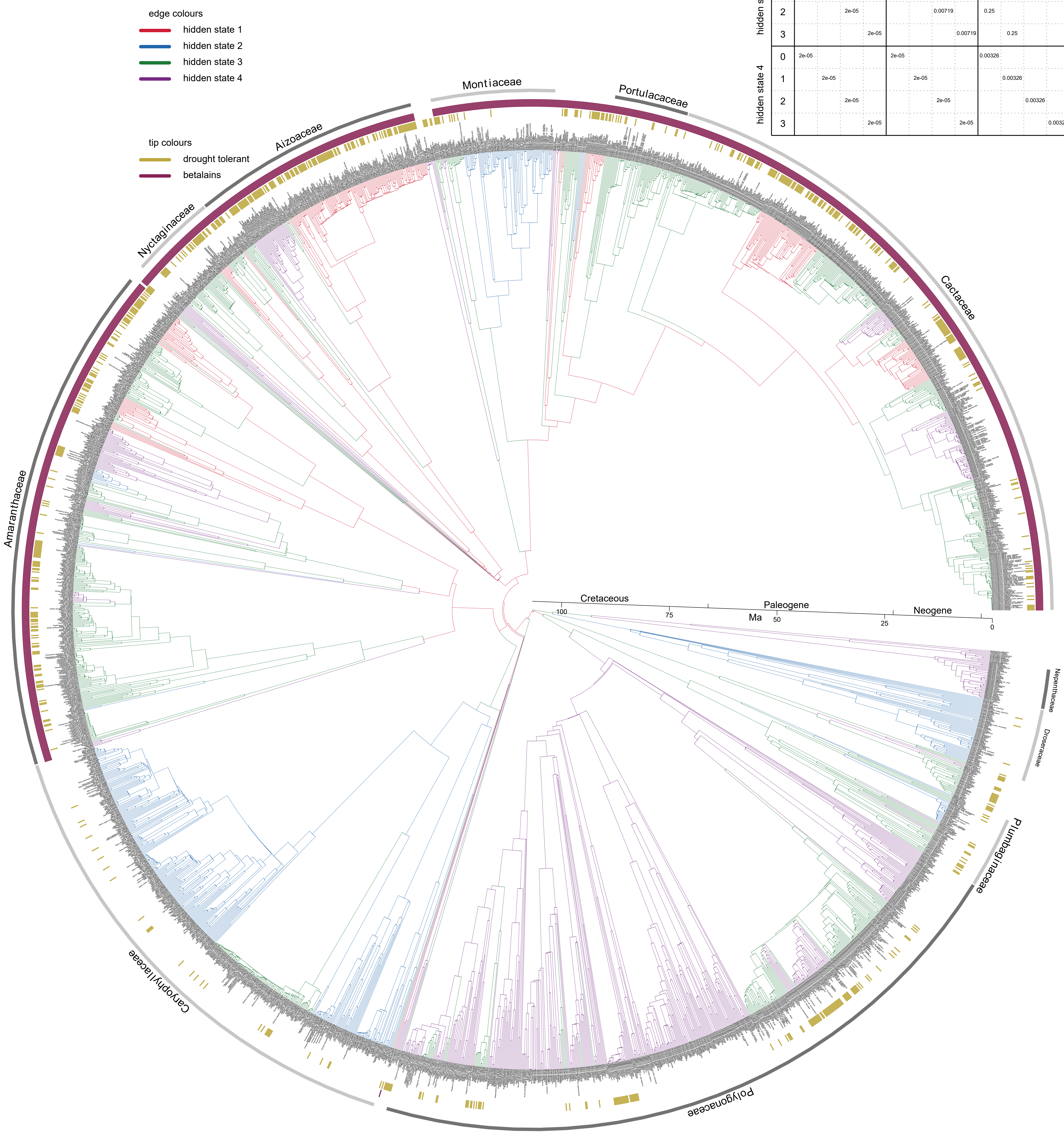

Figure S24. Best fitting model for the evolution of betalain pigmentation and drought tolerance (defined as at least 75% of specimens in arid climates as per Köppen climate categories). Branch colours show which branches are evolving under a given hidden state, matrix shows the rates for each hidden state.

0 = not drought tolerant + no betalains, 1 = not drought tolerant + betalains
2 = drought tolerant + no betalains, 3 = drought tolerant + betalains

		hidden state 1				hidden state 2				hidden state 3				hidden state 4			
		0	1	2	3	0	1	2	3	0	1	2	3	0	1	2	3
hidden state 1	0		0.00118	0.25		2e-05				0.01943				0.01285			
	1				0.25		2e-05				0.01943				0.01285		
	2	0.25			0.00118			2e-05				0.01943				0.01285	
	3		0.25						2e-05				0.01943				0.01285
hidden state 2	0	0.00811						0.00269		2e-05				0.00716			
	1		0.00811						0.00269		2e-05				0.00716		
	2			0.00811		0.06739						2e-05				0.00716	
	3				0.00811		0.06739						2e-05				0.00716
hidden state 3	0	0.06494				2e-05						2e-05		2e-05			
	1		0.06494				2e-05						2e-05		2e-05		
	2			0.06494				2e-05		0.00338						2e-05	
	3				0.06494				2e-05		0.00338						2e-05
hidden state 4	0	2e-05				2e-05				0.01969						2e-05	
	1		2e-05				2e-05				0.01969						2e-05
	2			2e-05				2e-05				0.01969		2e-05			
	3				2e-05				2e-05				0.01969		2e-05		

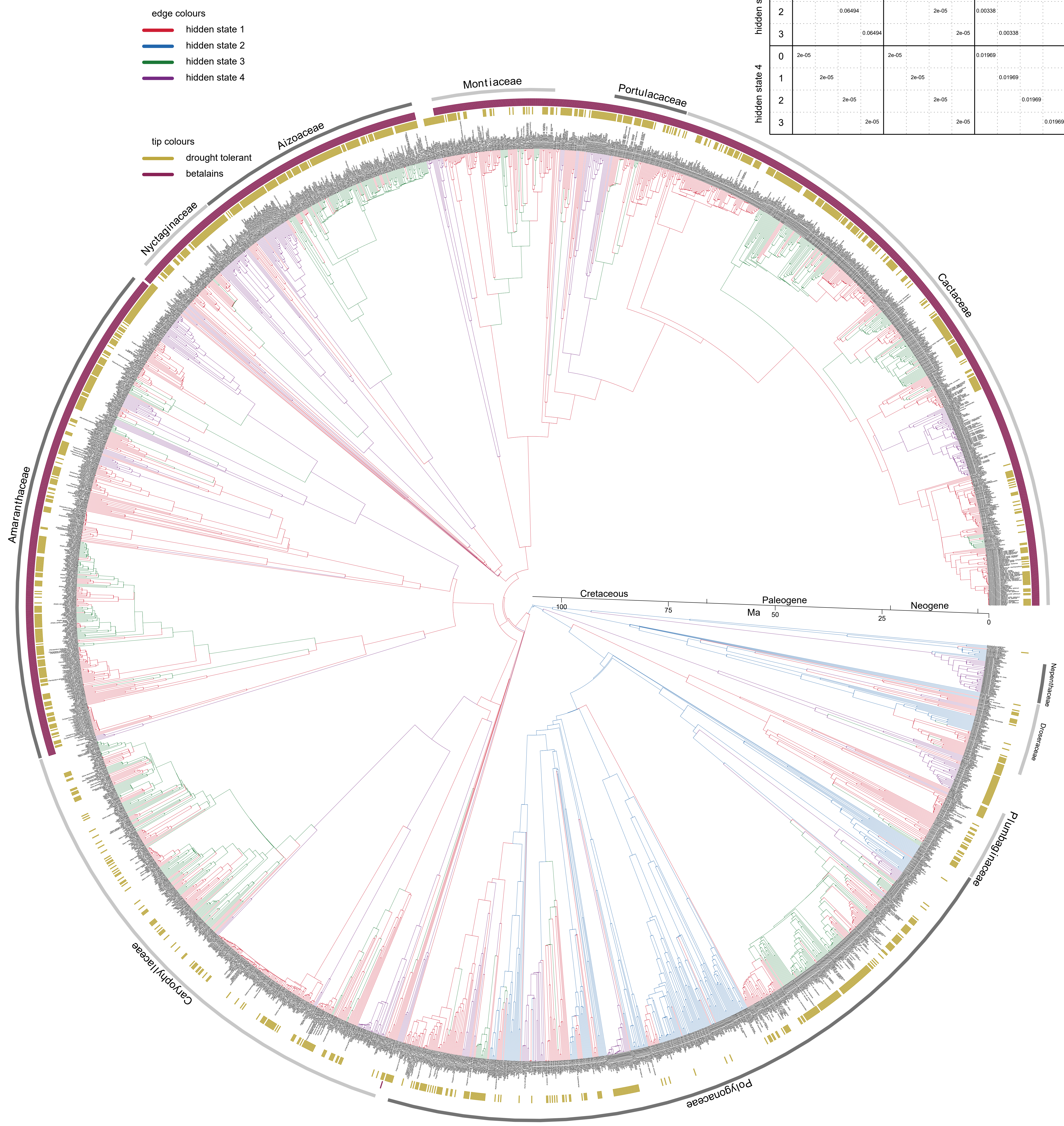

Figure S25. Best fitting model for the evolution of betalain pigmentation and drought tolerance (defined as at least 25% of specimens in arid climates as per Köppen climate categories). Branch colours show which branches are evolving under a given hidden state, matrix shows the rates for each hidden state.

0 = not drought tolerant + no betalains, 1 = not drought tolerant + betalains
2 = drought tolerant + no betalains, 3 = drought tolerant + betalains

		hidden state 1				hidden state 2				hidden state 3			
		0	1	2	3	0	1	2	3	0	1	2	3
hidden state 1	0		0.00874	0.14208		0.00355				0.01487			
	1				0.14208		0.00355				0.01487		
	2	0.2337			0.00874			0.00355				0.01487	
	3		0.2337						0.00355				0.01487
hidden state 2	0	2e-05						2e-05		2e-05			
	1		2e-05						2e-05		2e-05		
	2			2e-05		0.25						2e-05	
	3				2e-05		0.25						2e-05
hidden state 3	0	2e-05				0.00808						0.02374	
	1		2e-05				0.00808						0.02374
	2			2e-05				0.00808		0.25			
	3				2e-05				0.00808		0.25		

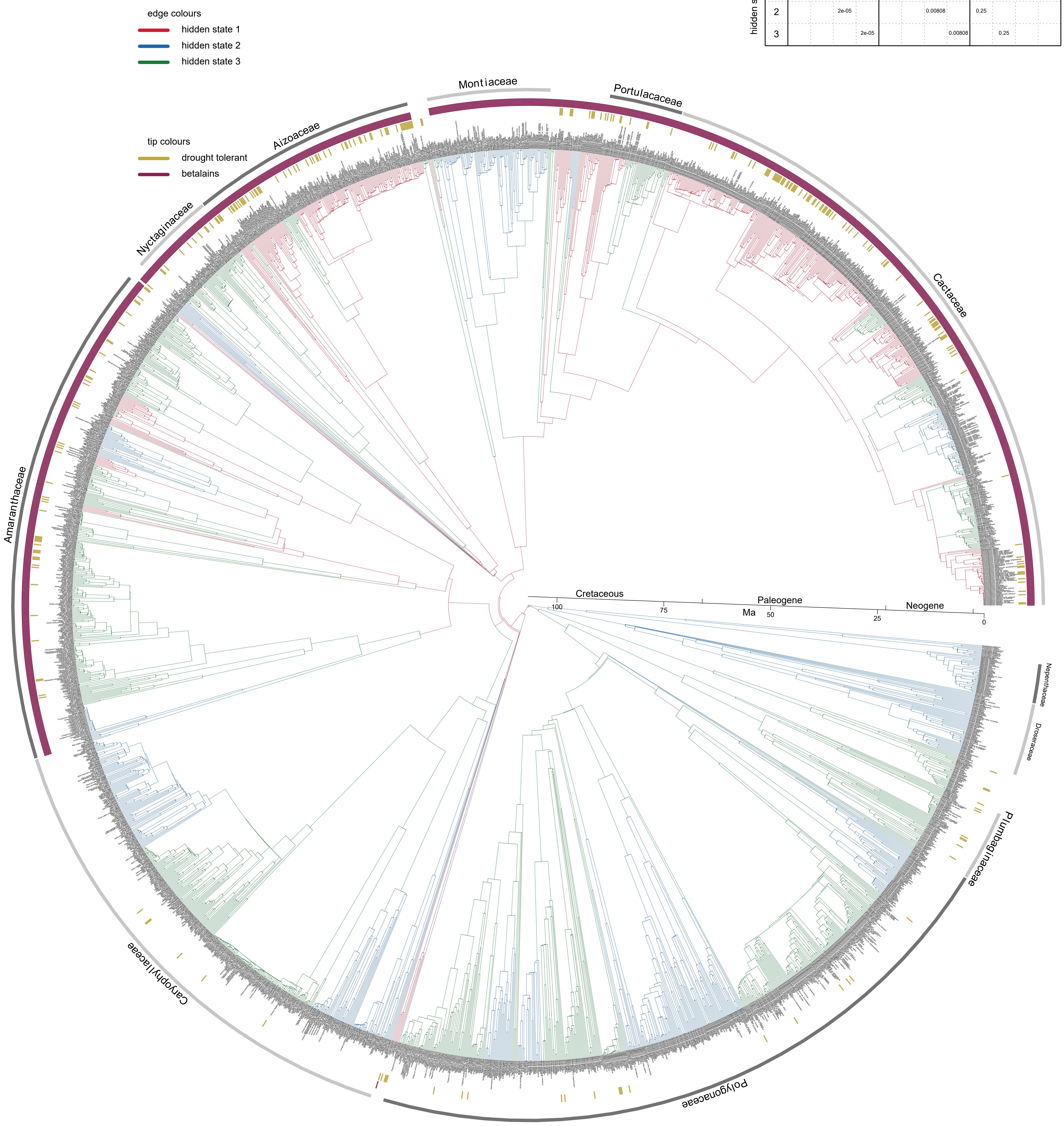

Figure S26. Best fitting model for the evolution of betalain pigmentation and drought tolerance (defined as at least 75% of specimens in hot arid climates as per Köppen climate categories). Branch colours show which branches are evolving under a given hidden state, matrix shows the rates for each hidden state.

0 = not drought tolerant + no betalains, 1 = not drought tolerant + betalains
2 = drought tolerant + no betalains, 3 = drought tolerant + betalains

		hidden state 1				hidden state 2				hidden state 3			
		0	1	2	3	0	1	2	3	0	1	2	3
hidden state 1	0		0.01649	0.17167		0.01096				0.00864			
	1				0.17167		0.01096				0.00864		
	2	0.25			0.01649			0.01096				0.00864	
	3		0.25						0.01096				0.00864
hidden state 2	0	2e-05						2e-05		0.00891			
	1		2e-05						2e-05		0.00891		
	2			2e-05		0.00801						0.00891	
	3				2e-05		0.00801						0.00891
hidden state 3	0	2e-05				0.00269						0.05204	
	1		2e-05				0.00269						0.05204
	2			2e-05				0.00269		0.25			
	3				2e-05				0.00269		0.25		

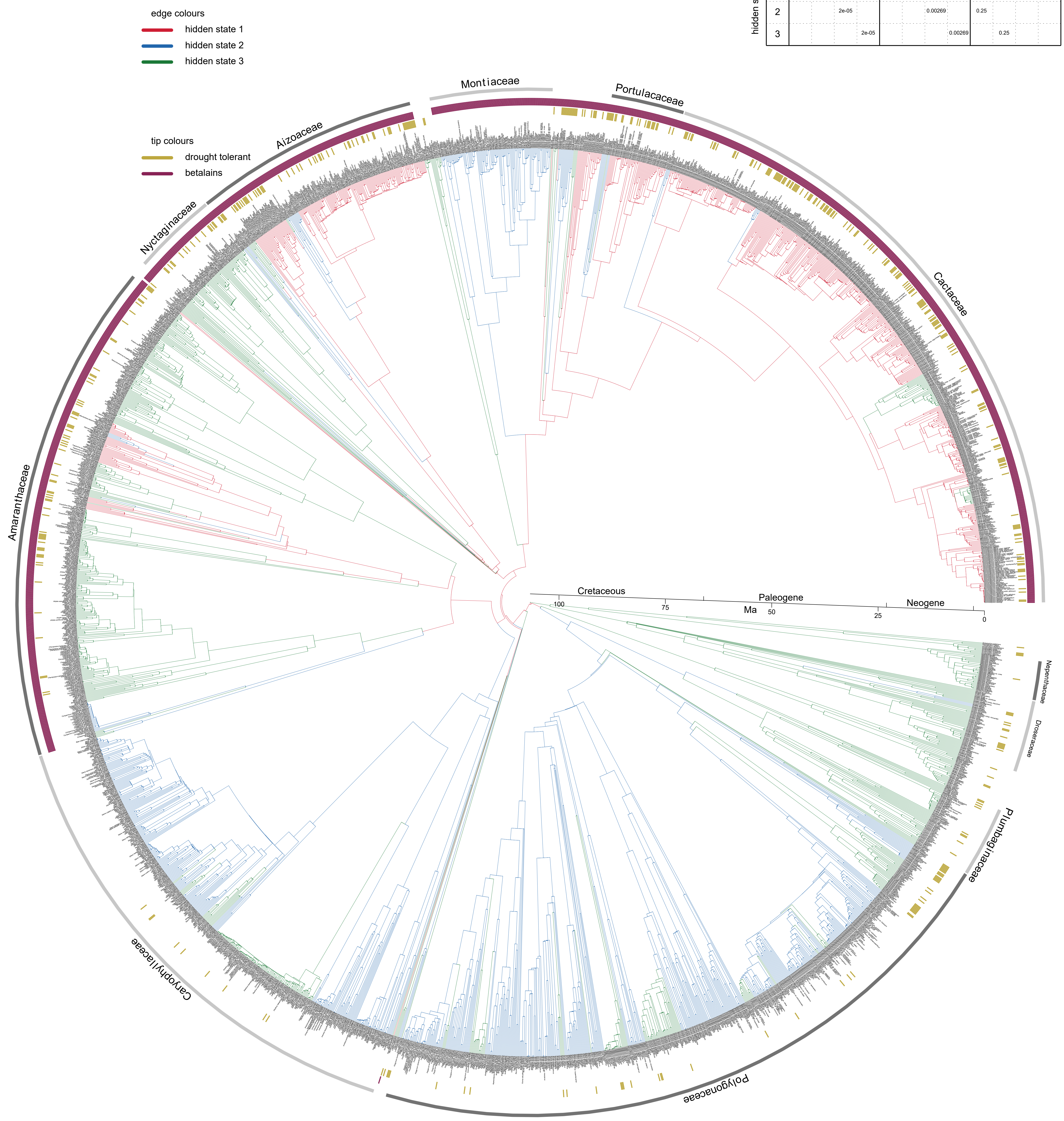

Figure S27. Best fitting model for the evolution of betalain pigmentation and drought tolerance (defined as at least 75% of specimens in hot arid climates or hot savannah as per Köppen climate categories). Branch colours show which branches are evolving under a given hidden state, matrix shows the rates for each hidden state.

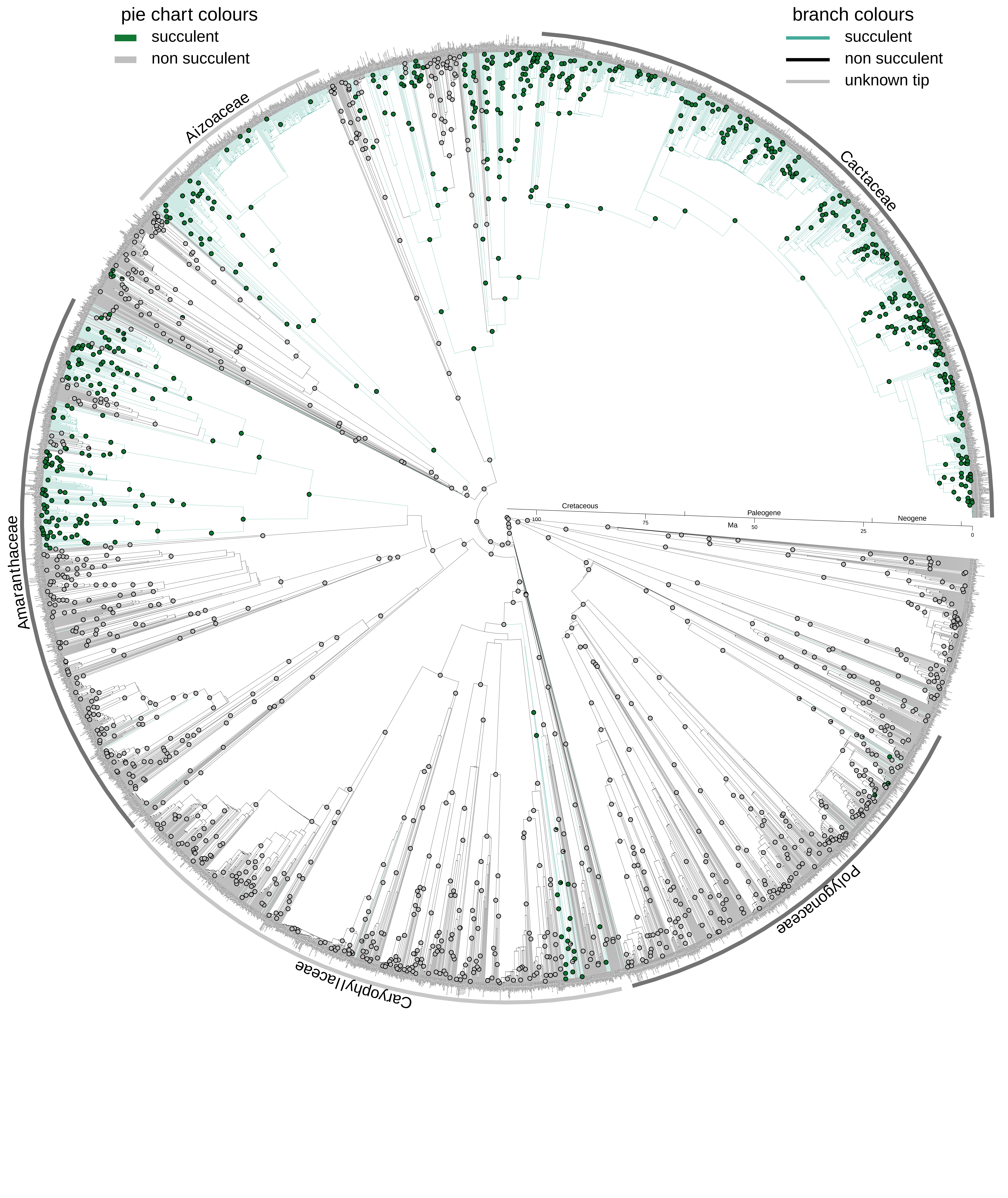

Figure S28. Reconstruction of evolution of succulence on the species-level phylogenetic tree constrained to the RAxML topology of Walker et al. (2018) and using the best fitting model (ARD). Pie charts refer to node states reconstructed across all bootstrap trees.

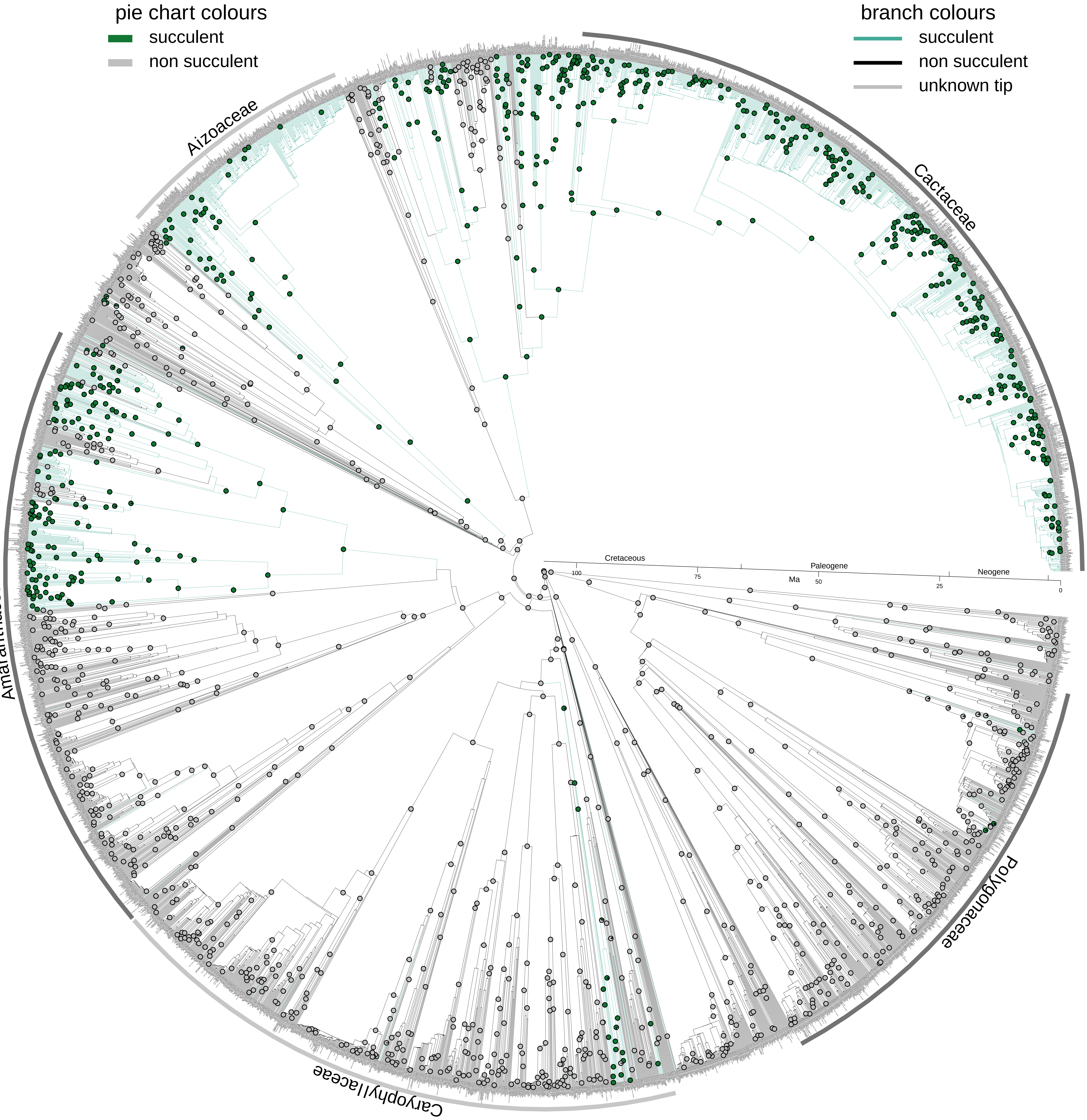

Figure S29. Reconstruction of evolution of succulence on the species-level phylogenetic tree constrained to the ASTRAL topology of Walker et al. (2018) and using the best fitting model (ARD). Pie charts refer to node states reconstructed across all bootstrap trees.

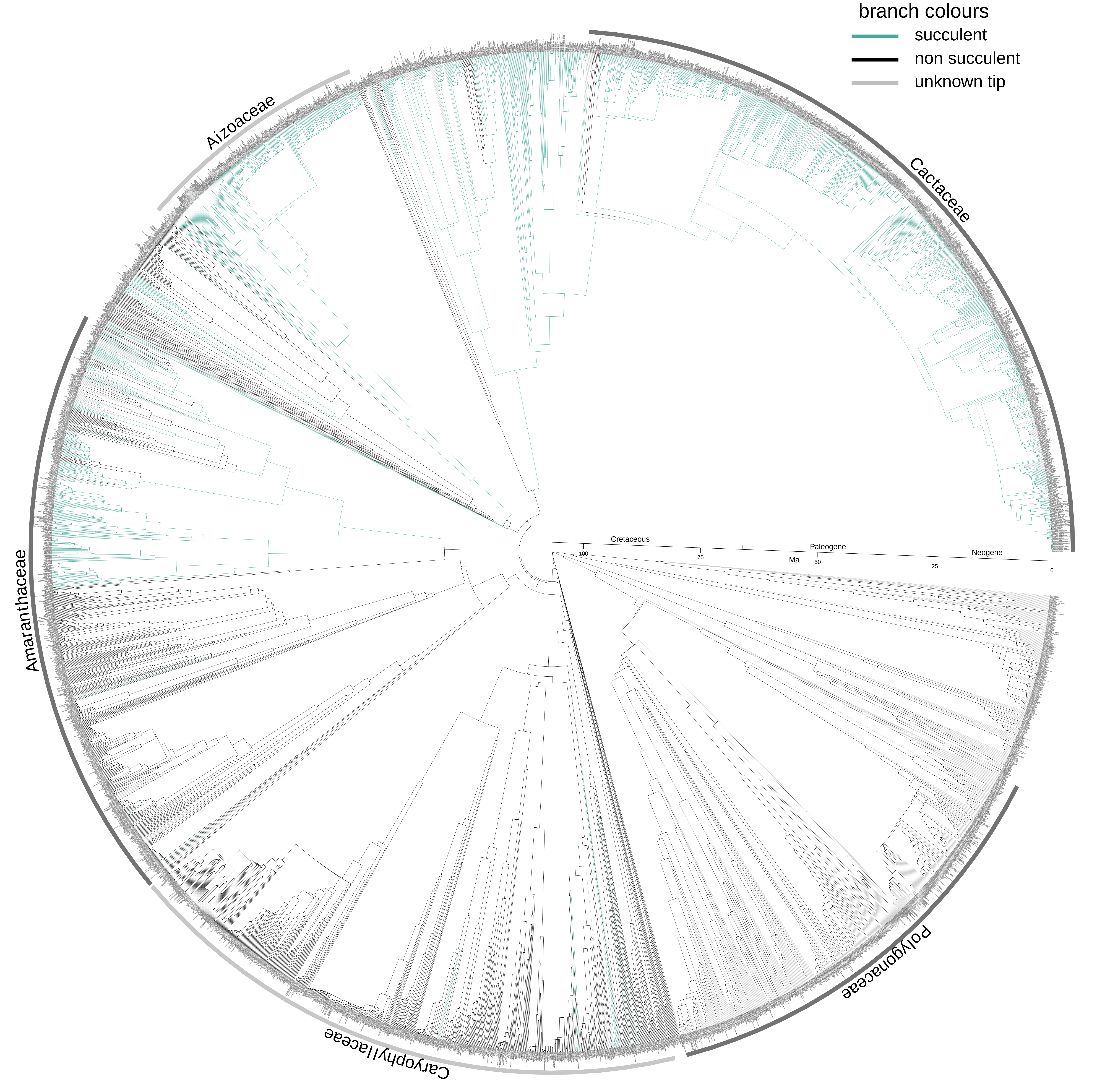

Figure S30. Reconstruction of evolution of succulence on the species-level phylogenetic tree constrained to the RAxML topology of Walker et al. (2018), using the relaxed definition of succulence from Vásquez-Cruz et al. (2025) and using the best fitting model (ER)

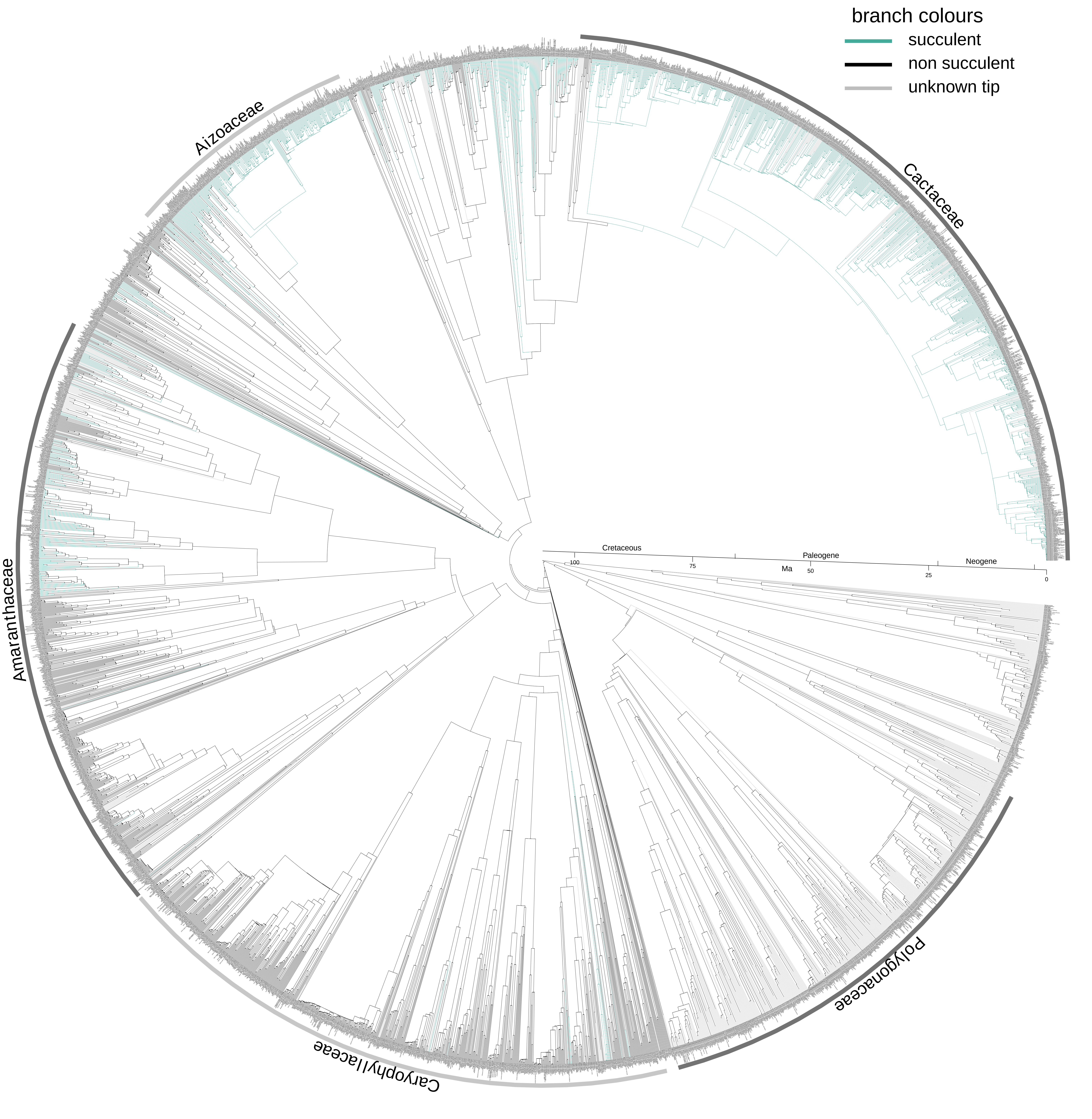

Figure S31. Reconstruction of evolution of succulence on the species-level phylogenetic tree constrained to the RAxML topology of Walker et al. (2018), using the strict definition of succulence from Vásquez-Cruz et al. (2025) and using the best fitting model (ER)

0 = not drought tolerant + not succulent, 1 = not drought tolerant + succulent
2 = drought tolerant + not succulent, 3 = drought tolerant + succulent

		hidden state 1				hidden state 2				hidden state 3			
		0	1	2	3	0	1	2	3	0	1	2	3
hidden state 1	0		2e-05	0.20159		0.01066				0.02464			
	1	0.00124			0.20159		0.01066				0.02464		
	2	0.25			2e-05			0.01066				0.02464	
	3		0.25	2e-05					0.01066				0.02464
hidden state 2	0	0.00645					2e-05	0.00088		2e-05			
	1		0.00645			0.00124			0.00088		2e-05		
	2			0.00645		0.0226			2e-05			2e-05	
	3				0.00645		0.0226	2e-05					2e-05
hidden state 3	0	0.04135				0.00552					0.12696	0.02393	
	1		0.04135				0.00552			0.00124			0.02393
	2			0.04135				0.00552		2e-05			0.12696
	3				0.04135				0.00552		2e-05	2e-05	

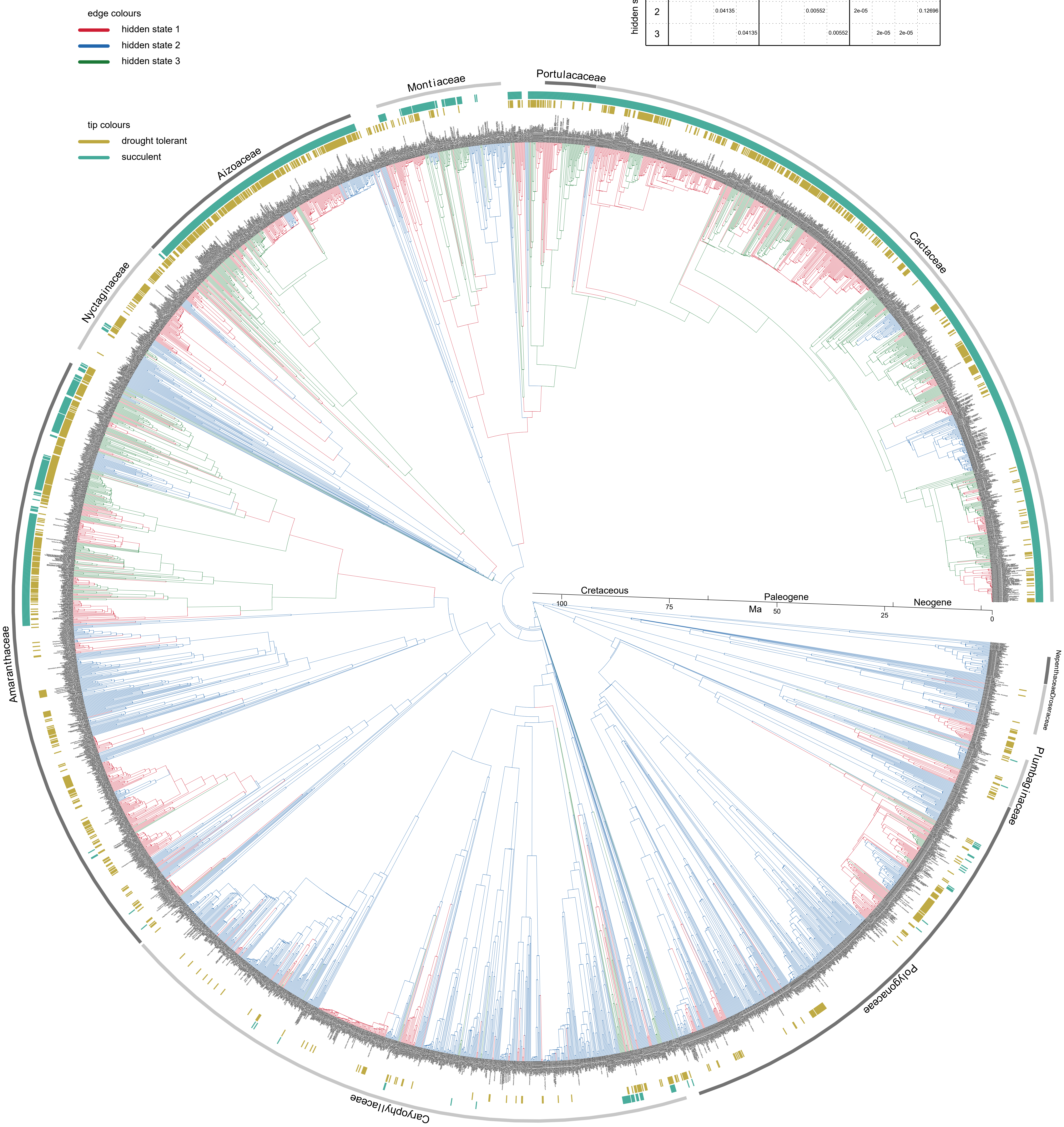

Figure S32. Best fitting model for the evolution of succulence and drought tolerance (defined as at least 75% of specimens in arid climates as per Köppen climate categories). Branch colours show which branches are evolving under a given hidden state, matrix shows the rates for each hidden state.

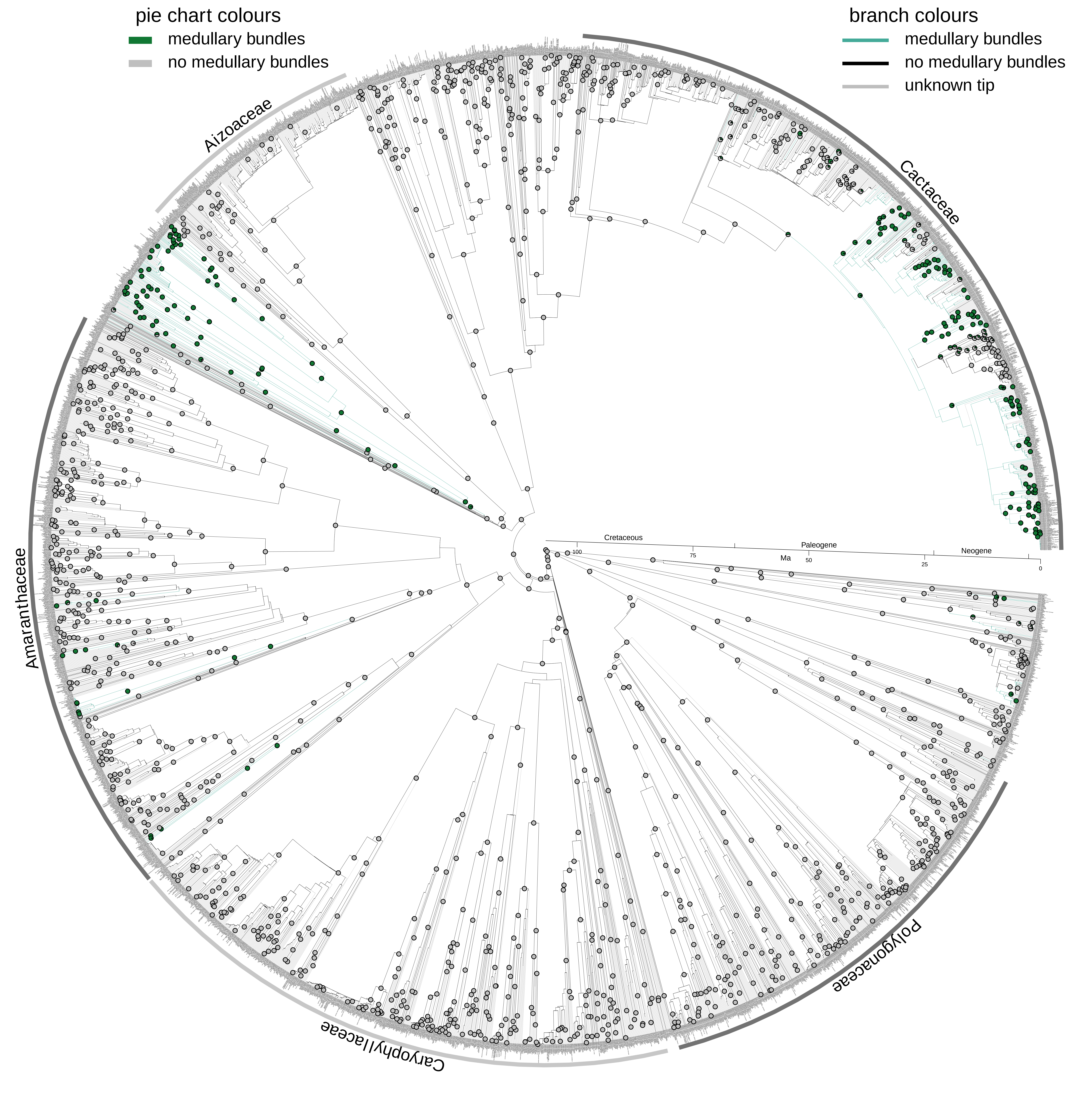

Figure S33. Reconstruction of the evolution of medullary bundles on the species-level phylogenetic tree constrained to the RAxML topology of Walker et al. (2018), and using the best fitting model (ARD). Pie charts refer to node states reconstructed across all bootstrap trees.

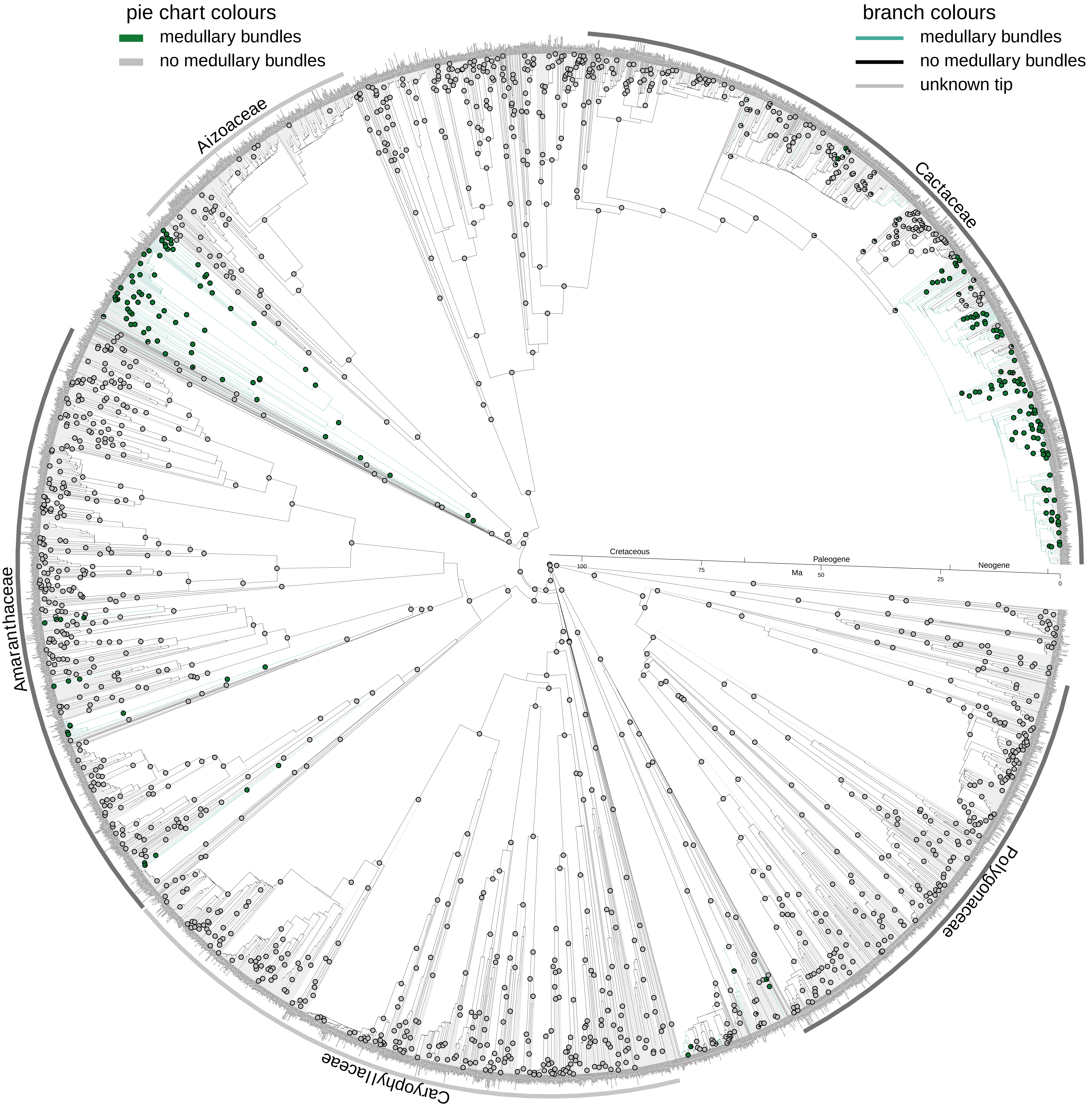

Figure S34. Reconstruction of the evolution of medullary bundles on the species-level phylogenetic tree constrained to the ASTRAL topology of Walker et al. (2018), and using the best fitting model (ARD). Pie charts refer to node states reconstructed across all bootstrap trees.

0 = not drought tolerant + no medullary bundles, 1 = not drought tolerant + medullary bundles
2 = drought tolerant + not medullary bundles, 3 = drought tolerant + medullary bundles

		hidden state 1				hidden state 2				hidden state 3			
		0	1	2	3	0	1	2	3	0	1	2	3
hidden state 1	0		2e-05	0.00944		2e-05				0.01593			
	1	0.01399			0.00944		2e-05				0.01593		
	2	0.1825			2e-05			2e-05				0.01593	
	3		0.1825	0.01399					2e-05				0.01593
hidden state 2	0	2e-05					2e-05	0.0413		2e-05			
	1		2e-05			2e-05			0.0413		2e-05		
	2			2e-05		0.04746			2e-05			2e-05	
	3				2e-05		0.04746	2e-05					2e-05
hidden state 3	0	0.03844				0.01362					0.05092	2e-05	
	1		0.03844				0.01362			0.25			2e-05
	2			0.03844				0.01362		0.00509			0.05092
	3				0.03844				0.01362		0.00509	0.25	

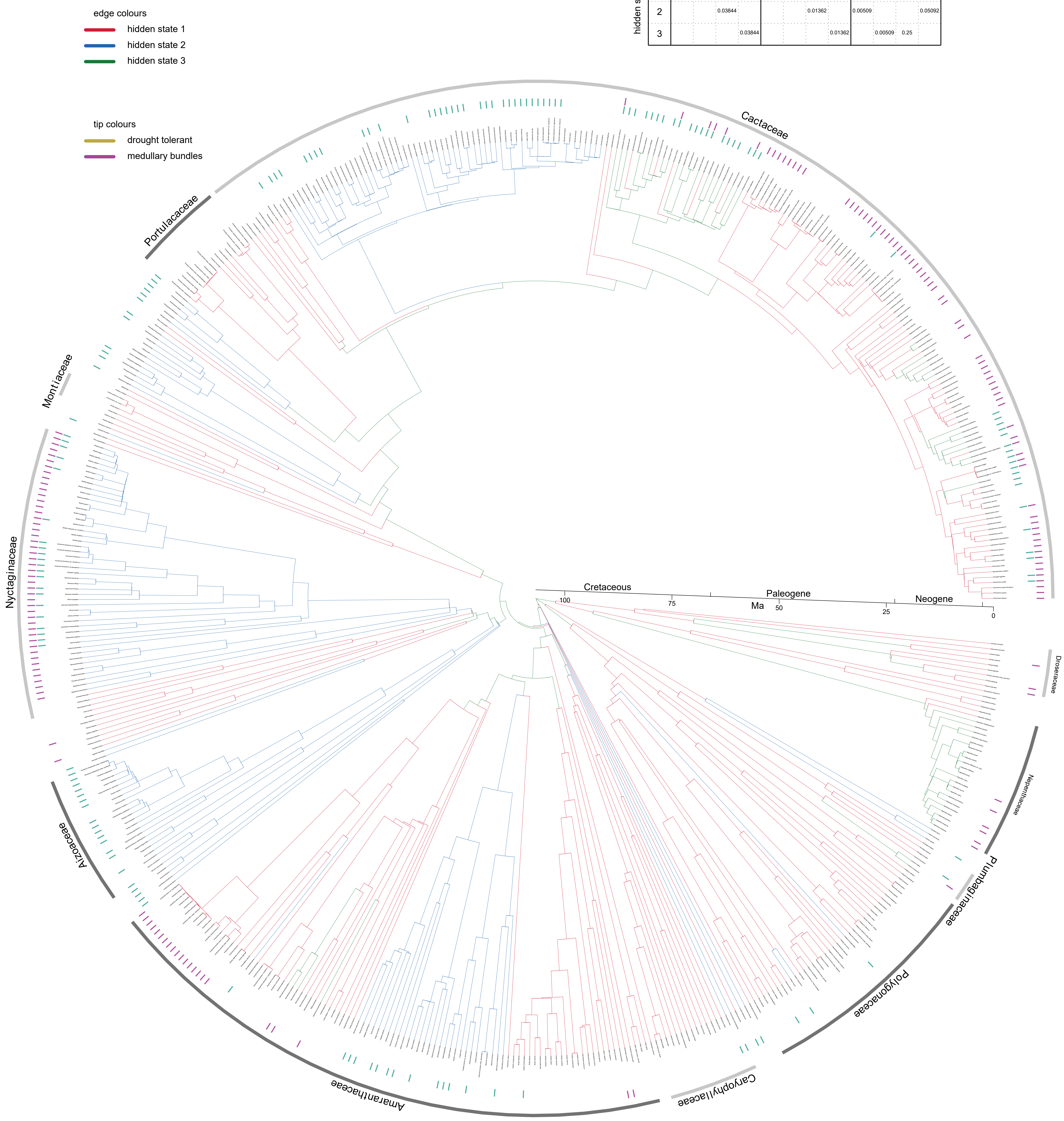

Figure S35. Best fitting model for the evolution of medullary bundles and drought tolerance (defined as at least 75% of specimens in arid climates as per Köppen climate categories). Branch colours show which branches are evolving under a given hidden state, matrix shows the rates for each hidden state.

0 = no succulent + no betalains, 1 = no succulent + betalains, 2 = succulent + no betalains, 3 = succulent + betalains

		hidden state 1				hidden state 2				hidden state 3			
		0	1	2	3	0	1	2	3	0	1	2	3
hidden state 1	0		2e-05	2e-05		0.00033				2e-05			
	1				2e-05		0.00033				2e-05		
	2	2e-05			2e-05			0.00033				2e-05	
	3		2e-05						0.00033				2e-05
hidden state 2	0	0.02482					2e-05	0.02817		2e-05			
	1		0.02482						0.02817		2e-05		
	2			0.02482		0.25			2e-05			2e-05	
	3				0.02482		0.25						2e-05
hidden state 3	0	0.00805				0.0318					0.02413	0.25	
	1		0.00805				0.0318						0.25
	2			0.00805				0.0318		0.02773			0.02413
	3				0.00805				0.0318		0.02773		

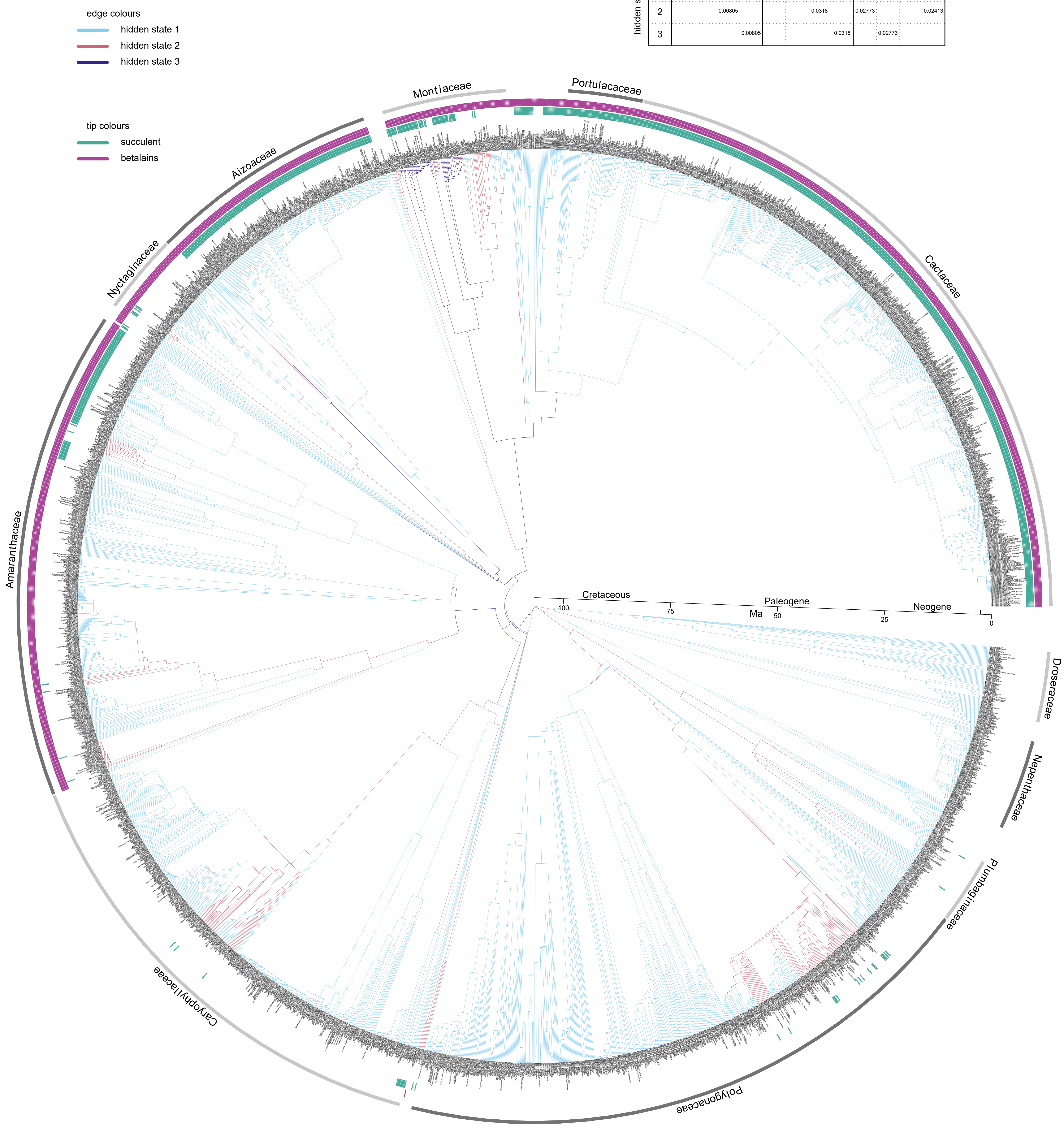

Figure S36. Best fitting model for the evolution of succulence and betalains.

0 = no bundles + no betalains, 1 = no bundles + betalains, 2 = bundles + no betalains, 3 = bundles + betalains

		hidden state 1				hidden state 2				hidden state 3			
		0	1	2	3	0	1	2	3	0	1	2	3
hidden state 1	0		0.04309	2e-05		0.00762				0.07983			
	1				2e-05		0.00762				0.07983		
	2	0.25			0.04309			0.00762				0.07983	
	3		2e-05						0.00762				0.07983
hidden state 2	0	2e-05						0.05439		2e-05			
	1		2e-05						0.05439		2e-05		
	2			2e-05		0.25						2e-05	
	3				2e-05		2e-05						2e-05
hidden state 3	0	2e-05				0.00226						2e-05	
	1		2e-05				0.00226						2e-05
	2			2e-05				0.00226		0.25			
	3				2e-05				0.00226		2e-05		

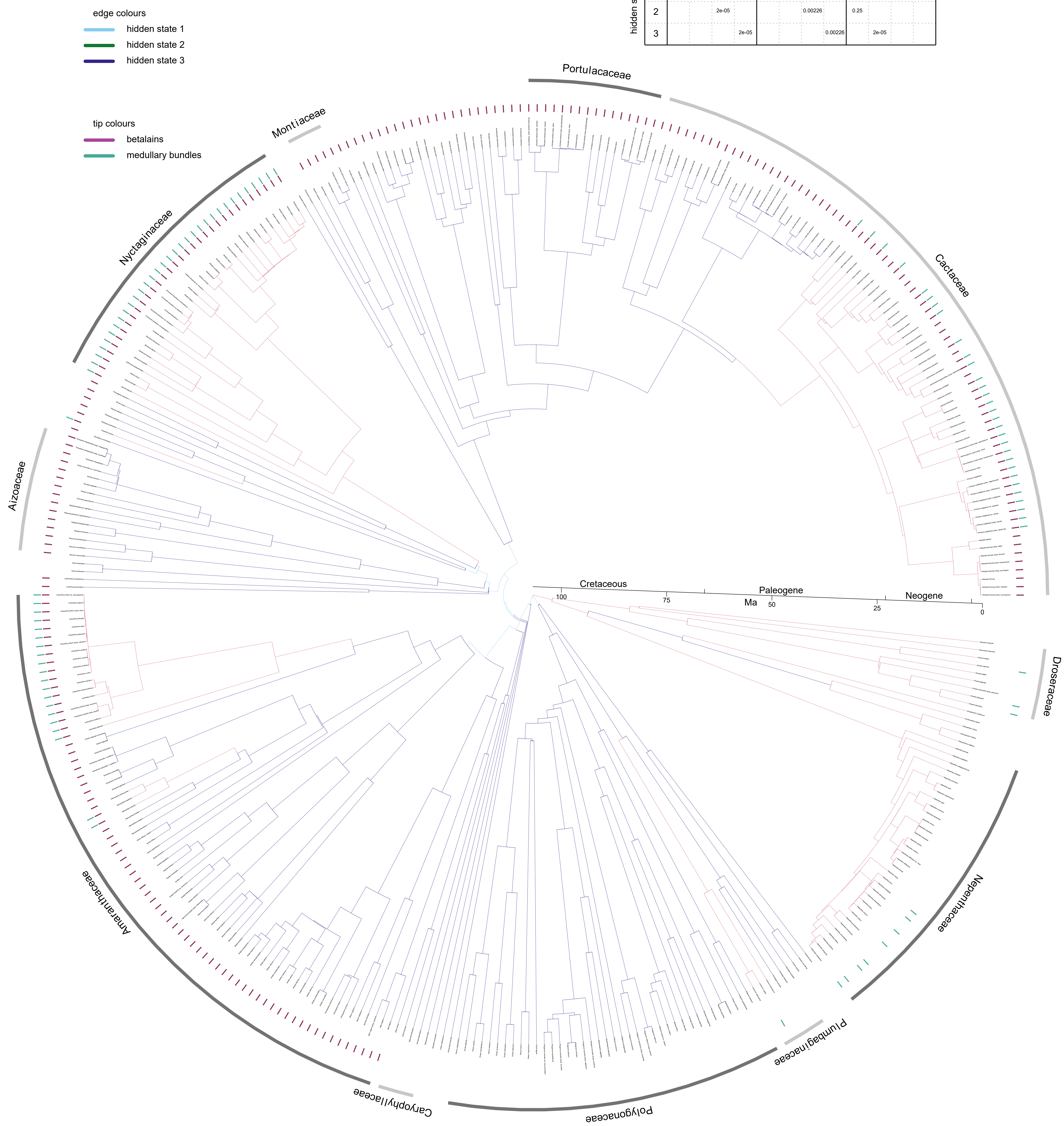

Figure S37. Best fitting model for the evolution of medullary bundles and betalains.

0 = no bundles + not succulent, 1 = no bundles + succulent, 2 = bundles + not succulent, 3 = bundles + succulent

		hidden state 1				hidden state 2				hidden state 3			
		0	1	2	3	0	1	2	3	0	1	2	3
hidden state 1	0		0.03427	0.00047		0.01018				0.00273			
	1	2e-05			0.00047		0.01018				0.00273		
	2	0.03997			0.03427			0.01018				0.00273	
	3		0.03997	2e-05					0.01018				0.00273
hidden state 2	0	2e-05					2e-05	2e-05		2e-05			
	1		2e-05			2e-05			2e-05		2e-05		
	2			2e-05		2e-05			2e-05			2e-05	
	3				2e-05		2e-05	2e-05					2e-05
hidden state 3	0	0.00541				0.00916					2e-05	0.05491	
	1		0.00541				0.00916			2e-05			0.05491
	2			0.00541				0.00916		0.25			2e-05
	3				0.00541				0.00916		0.25	2e-05	

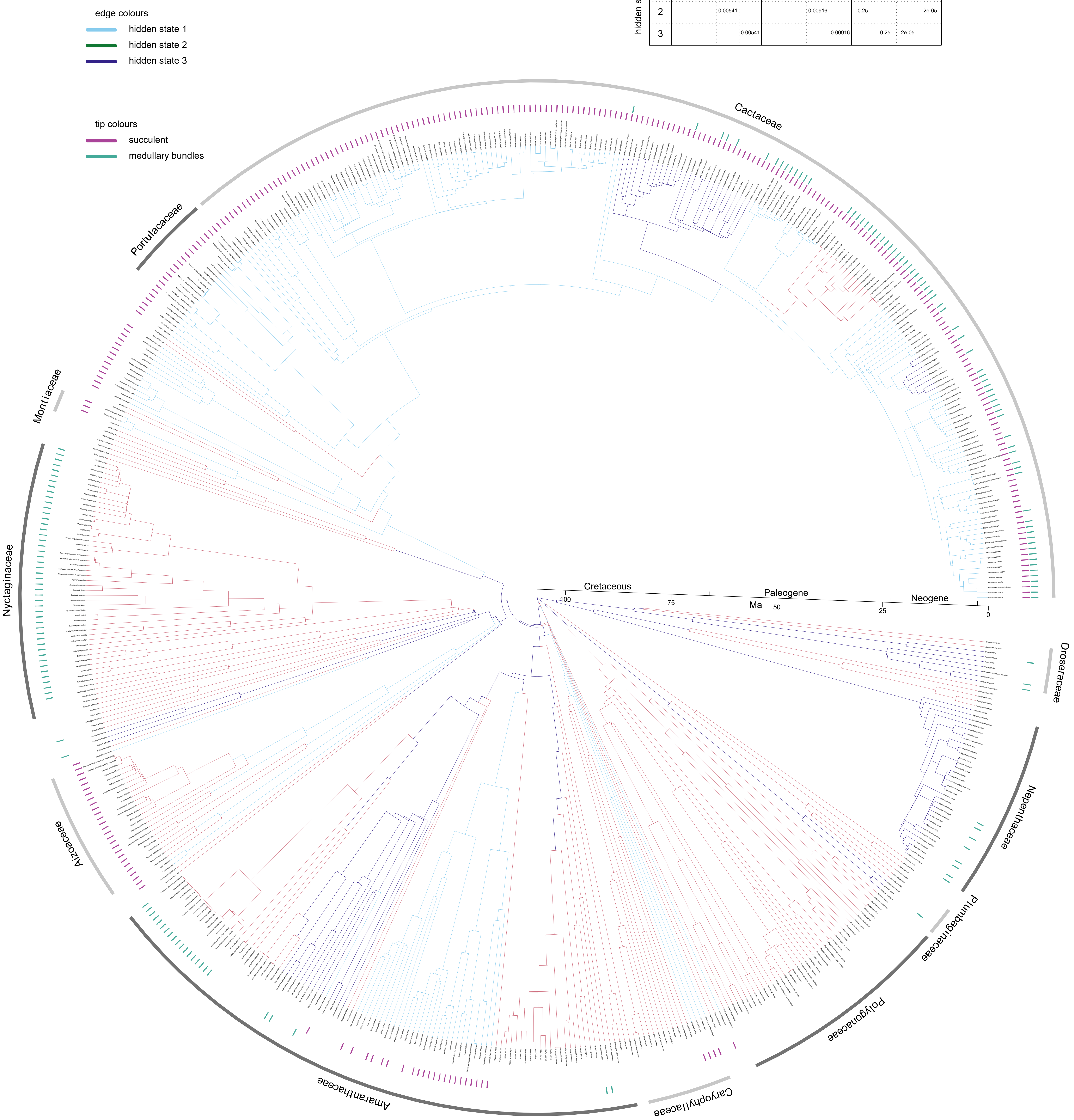

Figure S38. Best fitting model for the evolution of medullary bundles and succulence.

References

Arakaki M, Christin PA, Nyffler R, Lendel A, Eggli U, Ogburn RM, Spriggs E, Moore MJ, Edwards EJ. 2011. Proc Natl Acad Sci USA. 108:8379-8384.

Beucher F. 1975. Etude palynologique de formations nrog~nes et quaternaires du Sahara Nord-Occidental. Centre Rech. Zones Arides, Srrie Grologie no. 20. Paris, C.N.R.S. Thesis Univ. Paris.

Gao R, Zhao C, Qiao X, Zheng Y, Yan F, Wan C. 1999. Cretaceous oil strata palynology from Songliao Basin, Geological Publishing House, Beijing.

Gonzalez-Guzman AE. 1967. A palynological study on the Upper Los Cuervos and Mirador formations (Lower and Middle Eocene, Tibti area, Colombia). Thesis. E. J. Brill, Leiden. Pages 1-68.

Krutzsch W. 1970. Die stratigraphisch verwertbaren Sporen-und Pollenformen des mitteleuropiiischen Alttertiärs. Jahrb Geol Bot. 3:309-379.

Magallón S, Gómez-Acevedo S, Sánchez-Reyes LL, Hernández-Hernández T. 2015. A metacalibrated time-tree documents the early rise of flowering plant phylogenetic diversity. New Phyt. 207:437-453.

Manchester SR. and O'Leary E. 2010. Distribution and identification of fin-winged fruits. Bot Rev. 76:1–82.

Muller J. 1981. Fossil pollen records of extant angiosperms. Bot Rev. 47:1–142.

Smith SA, Brown JW, Yang Y, Bruenn R, Drummond CP, Brockington SF, Walker JF, Last N, Douglas NA, Moore MJ. 2018. Disparity, diversity, and duplications in the Caryophyllales. New Phyt. 217:836-854.

Song Z, Wang WM, Huang F. 2004. Fossil pollen records of extant angiosperms in China. Bot Rev. 70:425-458.

Song Z, Zheng Y, Lind, Ye P, Wang C, Zhou S. 1981. Cretaceous-Tertiary palynological assemblages from Jiangsu. Geological Publishing House, Beijing (in Chinese; English abstract).

Takahashi M. 1997. Fossil spores and pollen grains of Cretaceous (Upper Campanian) from Sakhalin, Russia. Journal of Plant Research. 110:283-298.
